# Origin and evolution of acrocentric chromosomes in human and great apes

**DOI:** 10.64898/2025.12.22.696095

**Published:** 2025-12-23

**Authors:** Steven J. Solar, Prajna Hebbar, Leonardo Gomes de Lima, Alex Sweeten, Arang Rhie, Tamara Potapova, Luciana de Gennaro, Andrea Guarracino, Juhyun Kim, Brandon D. Pickett, Benedict Paten, Melissa A. Wilson, Sergey Koren, Erik Garrison, Evan E. Eichler, Mario Ventura, Jennifer L. Gerton, Adam M. Phillippy

**Affiliations:** 1 Genome Informatics Section, Center for Genomics and Data Science Research, National Human Genome Research Institute, National Institutes of Health, Bethesda, MD, USA; 2 UC Santa Cruz Genomics Institute, University of California, Santa Cruz, Santa Cruz, CA, USA; 3 Stowers Institute for Medical Research, Kansas City, MO, USA; 4 Department of Biosciences, Biotechnology and Environment, University of Bari Aldo Moro, Bari, Italy; 5 Department of Genetics, Genomics and Informatics, University of Tennessee Health Science Center, Memphis, TN, USA; 6 Comparative Genomics and Reproductive Health Section, Center for Genomics and Data Science Research, National Human Genome Research Institute, National Institutes of Health, Bethesda, MD, USA; 7 Department of Genome Sciences, University of Washington School of Medicine, Seattle, WA, USA; 8 Howard Hughes Medical Institute, University of Washington, Seattle, WA, USA

## Abstract

The short arms of human acrocentric chromosomes are characterized by nucleolar organizer regions essential for ribosome biogenesis, but their highly repetitive nature has hindered genomic analysis. Leveraging the recently completed genomes of all major ape lineages, we identified recurrent features of their acrocentrics, including enriched repeat classes, centromere repositioning by whole-arm inversion, interchromosomal sequence exchange, and birth-and-death evolution of multiple gene families. Together, these processes have enabled the repeated amplification and diversification of the *FRG1* gene family over 25 million years of ape evolution, and, in gorilla, the formation and amplification of a novel *IGSF3*-*GGT* fusion gene under positive selection. Similar evolutionary events also explain the distribution of segmental duplications and heterochromatin in the modern human genome, predisposing it to karyotypic abnormalities such as Robertsonian translocations. Our findings highlight acrocentric chromosomes as key drivers of evolution in the great apes, with implications for speciation, adaptation, and clinical genomics.

## Introduction

The evolutionary history of the apes is well established, with gibbons diverging before the great ape common ancestor, followed by splits from orangutan, gorilla, and chimp^1^. This tree was first determined using molecular and cytogenetic techniques, including chromosome painting, banding, and fluorescence *in situ* hybridization (FISH) to identify relationships between segments of human and non-human ape chromosomes^2–4^. It was similarly established that mammalian karyotype evolution occurs primarily through the rearrangement of syntenic blocks of DNA, in the form of chromosome fusion, fission, translocation, or inversion^5^, and that these large-scale structural changes can facilitate adaptation and speciation^6^. Conveniently, most great ape chromosomes are fully syntenic with their human homologs, with the exception of a telomeric fusion of ancestral chromosomes 2A and 2B in human^7,8^ and a balanced translocation of human homologs 5 and 17 in gorilla^9^. Within chromosomes, inversions are the most common large-scale structural variants between the great apes^10,11^, but until recently, we lacked the complete genome sequences needed to fully characterize these events, especially within the short arms of the acrocentric chromosomes.

With the recent sequencing of complete, telomere-to-telomere (T2T) reference genomes for human and all major ape lineages^10,12–14^, it is now possible to investigate the structural evolution of ape karyotypes at the sequence level, including the 12.5–27.3% of bases within each genome that fail to align to the other apes^10^. Here we focus on the complete, T2T genomes of six great ape and one gibbon species: *Homo sapiens* (human, HSA), *Pan paniscus* (bonobo, PPA), *Pan troglodytes* (chimp, PTR), *Gorilla gorilla gorilla* (gorilla, GGO), *Pongo abelii* (Sumatran orangutan, PAB), *Pongo pygmaeus* (Bornean orangutan, PPY), and *Symphalangus syndactylus* (siamang, SSY). Advances in long-read sequencing^15,16^ and genome assembly^17,18^ have unlocked previously dark regions of the genome including segmental duplications^19^, amplified gene families^20^, chromosomal fusions^21^, and satellite tandem repeats^22–24^. Such repeats are commonly enriched on the short arms of acrocentric chromosomes, and because of their tendency to recombine, are common sources of structural variation in the genome.

The short arms of acrocentric chromosomes often contain a nucleolar organizer region (NOR) comprising a tandem array of 45S ribosomal DNA (rDNA) repeats, which are transcribed to make the ribosomal RNA (rRNA) genes and around which the nucleolus forms during interphase^25,26^. Because of this relationship between acrocentric chromosomes and NORs, the two concepts are often conflated, but not all acrocentric chromosomes contain a NOR. Here we use the traditional cytogenetic definition of an acrocentric chromosome based solely on the asymmetric size of the arms^27^, by which human Chromosomes 13, 14, 15, 21, 22, and Y are classified as acrocentric^28^. We use NOR+ and NOR− to indicate the typical presence of a NOR on a chromosome, inferred by the presence of a well-formed rDNA array, but note that variable NOR loss or silencing can occur within individuals^29^. ChrY is often omitted from the list of acrocentrics since it is consistently NOR− in human, but it is NOR+ in orangutans^30^ and shares many other features of acrocentric chromosomes, such as satellite content^31^.

The short arms of acrocentric chromosomes are generally gene-poor, heterochromatic, and enriched for segmental duplications and satellite repeats. Surprisingly, despite being different chromosomes, the short arms of human acrocentric chromosomes share a median sequence identity of 99% with each other^12^. This high degree of similarity is a hallmark of concerted evolution^32^, first observed within the highly homogenized sequence of rDNAs^33^, but now understood to involve other regions of the short arms as well^34^. Termed pseudo-homolog regions (PHRs), this high degree of interchromosomal sequence similarity is maintained by various forms of recombination that homogenize the PHRs faster than they can diverge by mutation in a process known as molecular drive^35^. Interchromosomal recombination on the short arms may be promoted by their co-localization at the nucleolus and unique composition including rDNA and SST1 repeats^36^. Here, we use the words “exchange” and “recombination” interchangeably to mean the transfer of sequence information between loci by any means, including allelic and non-allelic crossing over, gene conversion, template switching, sister chromatid exchange, etc.

Given their propensity to recombine, it is not surprising that the number and identity of acrocentric chromosomes vary widely across the apes. Among the genomes included here, both orangutan species contain up to 10 NOR+ acrocentric chromosomes per haplotype, including Chromosome Y, while siamang contains as few as one^10,37,38^. Human, chimp, and gorilla each have a unique set of NOR+ acrocentrics. Robertsonian chromosomes resulting from the fusion of two acrocentric chromosomes are the most common karyotypic abnormality in humans (1 in 800 births)^21^, and the nonrandom segregation of Robertsonians by meiotic drive has been linked to speciation in mammals^39^. However, aside from human Chromosome 2, no other fusions have been fixed in the great apes^40^. Instead, pericentric and paracentric inversions appear to be the primary driver of karyotype evolution in the great apes by relocating centromeres^41^ and driving speciation through suppressed meiotic recombination in heterokaryotypes^11^.

In this study, we survey the newly complete sequence of great ape acrocentric chromosomes and revisit their evolutionary history. Our analyses uncover common mechanisms of evolution across all great ape species, including (1) acrocentric-specific repeat classes, (2) recurrent acrocentric to (sub)metacentric conversion by short-arm inversion, (3) recombination between heterologous short arms, and (4) PHR-mediated amplification and diversification of genes by birth-and-death evolution. These shared processes suggest that acrocentric chromosomes have played key roles in ape genome function, speciation, and adaptation throughout their history.

## Results

### Organization of the acrocentric chromosomes

**Figure 1.**
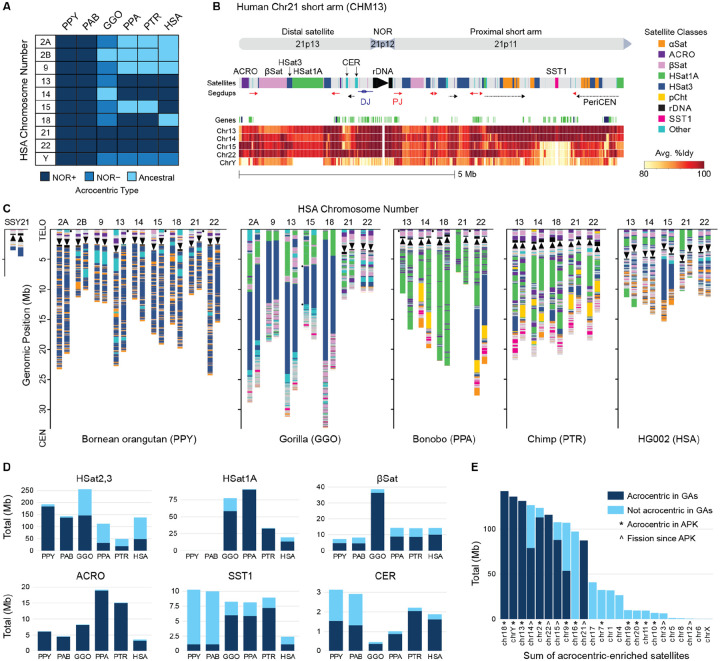
Short arms of the great ape acrocentric chromosomes. **(A)** NOR status of the 10 ancestral great ape acrocentric chromosomes in each species studied here. Ancestral refers to chromosomes that once were, but are no longer, acrocentric and have lost their NOR. **(B)** Example structure of a human Chr21 short arm from T2T-CHM13. Cytogenetic labels are given on top, followed by annotation of the satellite and segmental duplications, gene predictions (green: noncoding, blue: coding), and pairwise identity heatmap. The identity heatmap shows, for each window of Chr21, the highest identity of a window in the other acrocentrics (not necessarily at the same position). This replicates Figure 4 from^12^, but using the updated methods of this study. The rDNA array has been artificially shortened at the visible break in the heatmap. **(C)** Satellite annotations for all diploid pairs of acrocentric chromosomes. Maternal haplotypes are on the left, except for PPY and PTR where the parent of origin is unknown and the primary haplotype is on the left. Small dots indicate missing telomeres or remaining gaps in the assemblies. **(D)** Total bases in each species for six different satellite classes, partitioned by bases on acrocentric chromosomes (dark blue) or non-acrocentrics (light blue). **(E)** Total bases for the same six acrocentric-associated satellites summed over each homologous human chromosome in the great apes (GA) and stacked by that chromosome’s acrocentric status in each species. Ancestral primate karyotype (APK) predictions are from^42^.

Orangutans appear closest to the ancestral hominoid karyotype, for which human homologs HSA 2A, 2B, 9, 13, 14, 15, 18, 21, 22, and Y are predicted to have been acrocentric^42^. The complete genomes of both Sumatran and Bornean orangutans confirm that these chromosomes are all NOR+ (with the exception of occasional short-arm truncations), and all other acrocentrics in the great apes are homologous to one of these 10 chromosomes. Except for ChrY, NORs are only found on the short arms of acrocentric chromosomes. Thus, for each species we annotate these 10 chromosomes as NOR+ acrocentric, NOR− acrocentric, or ancestrally acrocentric (NOR−), and refer to them based on their human homolog (Figure 1A). Only the orangutans have maintained 10 NOR+ acrocentrics, with all other great apes having lost multiple NORs. HSA 21 and 22 have remained as NOR+ acrocentric chromosomes in all great apes, and in gorilla, these are the only two chromosomes that remain NOR+. Siamang has NORs only on ChrY and SSY21 (which is homologous to a piece of HSA3), but gibbon genomes have undergone at least eight chromosome fusions and are not representative of the ancestral ape karyotype^5,43^.

A typical short arm of a human acrocentric chromosome is arranged around the NOR with a smaller distal region towards the telomere and a larger proximal region towards the centromere (Figure 1B). Compared to the rest of the genome, the short arms are strongly enriched for satellite repeats, with 76% covered by satellites versus 7% for the rest of the autosomes in the human HG002 genome^14^. This includes human satellite 1 (HSat1A and HSat1B), human satellite 3 (HSat3), beta satellite (βSat), alpha satellite (αSat), Walu, ACRO, and SST1 repeats^12^. Note that human satellites 2 and 3 both derive from the same pentameric sequence (GGAAT), and we will refer to them jointly as HSat3 when not making a human-specific distinction^44^. Interspersed between this patchwork of satellites, and comprising 98% of the non-satellite sequences, are >100 kb stretches of some of the largest and most similar segmentally duplicated regions in the human genome^19^. Inter-chromosomal similarity varies across the short arm, and is highest in and around the rDNA arrays and other PHRs, such as the duplication associated with Robertsonian translocations on chromosomes 13, 14, and 21^21^.

Self and pairwise alignments of T2T-CHM13 revealed three general classes of segmentally duplicated sequence on the human short arms: distal junction (DJ), proximal junction (PJ), and pericentromeric sequence. The previously described DJ is a ~400 kb conserved region upstream of the rDNA array that typically appears as a single copy per chromosome and contains a >200 kb palindrome that encodes a long noncoding RNA associated with nucleolar organization^45^. In contrast, the PJ and pericentromeric sequences are variable in copy number, with the PJ widely duplicated on both sides of the NOR, and the pericentric duplications localized near the centromere. In human, these segmental duplicated sequences contain mostly non-coding or pseudogene annotations, including multiple expressed pseudogenes of *FRG1* (FSHD region gene 1)^12^, but also the protein-coding gene *TPTE* (Transmembrane Phosphatase with TEnsin homology) in the pericentric regions of Chr13 and Chr21^46^.

The other great ape acrocentrics follow a similar pattern of interspersed segmental duplications and an enrichment of satellites on the acrocentrics, but with varied content and organization (Figure 1C). On average, the great ape autosomal short arms are 87% satellite, with gorilla as the lowest outlier at 67% satellite. Like human, all rDNA arrays are uniformly oriented within a species (but not necessarily between species) and located towards the distal end of the chromosome, positioned an average of 1–3 Mb from the telomere and 7.5–20 Mb from the centromere. Notably, the uniform orientation and distal positioning of rDNA arrays within a genome would allow for recombination without the formation of a Robertsonian chromosome. In human, gorilla, and orangutan, rDNA transcription proceeds in the direction of the centromere, while in chimp and bonobo it is towards the telomere^45^. These inversions in chimp and bonobo include the DJ, keeping it always upstream of rDNA transcription, and implying a close functional link with the NOR^10^.

The orangutan proximal short arms stand out for their lack of HSat1A (0% of bases), unique tandem arrays of L1PA3 retrotransposons, and domination by HSat3 (49% PAB, 59% PPY) and monomeric αSat satellites (19% PAB, 16% PPY) (Supplementary Figs. S1–S2). In contrast, human, gorilla, chimp, and bonobo show a more varied composition of structures and satellites, including an invasion of HSat1A, increased enrichment of SST1 and βSat, and multi-megabase pericentric segmental duplications (Supplementary Figs. S3–S6). These pericentric duplications are conspicuously amplified on the NOR− gorilla acrocentrics (Supplementary Fig. S4), explaining their relatively lower satellite percentage. Siamang’s sole NOR+ autosome is comparatively miniscule, containing little sequence other than its large 4.5–10 Mb rDNA array and smaller HSat3 and βSat arrays (Supplementary Fig. S7).

Human satellite repeats are often referred to as “centromeric satellites” due to their association with human centromeres^22^. However, aside from centromeric αSat, this association is just as strong with the short arms of the acrocentric chromosomes (Figure 1D). In particular, 80% of all HSat2,3, HSat1A, βSat, ACRO, SST1, and CER bases are specific to acrocentric chromosomes in at least one great ape species (Supplementary Table S1). Furthermore, the vast majority of these satellites found elsewhere in the genome are on chromosomes that were previously acrocentric in the ancestral hominoid karyotype, suggesting they are relics from their acrocentric origin (Figure 1E). For example, HSA16 is not acrocentric in any of the great apes, but is predicted to have been acrocentric in the ancestral primate karyotype^42^ and contains both HSat3 and 45S rRNA pseudogenes in the pericentromeres of all great apes (Supplemental Fig. S8). Similarly, HSA20 contains many acrocentric-like features in the pericentromere, especially in the human, chimp, and gorilla genomes. All other non-acrocentric chromosomes with an enrichment of acrocentric satellites in their pericentromere are either predicted to have been acrocentric in the ancestral primate karyotype or derived from a fission event^5,42^, except for HSA 1 and 4, whose enrichment is primarily due to species-specific satellite expansions in human and gorilla, respectively, and HSA17, which has a balance of acrocentric satellites in all great ape species (Supplementary Fig. S9).

Aside from the 45S rDNA, ACRO is the most strongly and universally enriched repeat class on the great ape acrocentric chromosomes (Figure 1D). ACRO is a 6 kb composite repeat, comprising ACRO1, L1PA10, MER21B, and L1MB8 Dfam annotations^47^, that is primate-specific^48^ and enriched for nucleolar-associated H1.0 histones^49^. Intriguingly, ACRO shows evidence of expression in early embryogenesis, peaking at the 4-cell stage^50^, but the omission of most ACRO copies from earlier reference genomes has hindered prior analyses. Unlike the other satellite classes, ACRO appears almost exclusively on acrocentric chromosomes (>90% of ACRO bases, Supplementary Table S2), typically organized as highly self-similar tandem arrays up to a few hundred kb in length. ACRO is especially characteristic of the distal short arm, and at least one such array is consistently found on all NOR+ great ape chromosomes pointing to a potential functional association with the nucleolus (Figure 1C).

Less exclusive to the acrocentrics, but equally notable, is the ~2 kb primate-specific macrosatellite SST1^51^ (also known as NBL2), which displays unusual methylation patterns in certain cancers^52^ and has been implicated as a common breakpoint of human Robertsonian translocations^21^. SST1 repeats on the short arms of T2T-CHM13 are also highly methylated^12^, actively transcribed^23^, and enriched for PRDM9 binding motifs^34^, suggesting they may be more accessible and prone to recombination than the surrounding heterochromatin. Mirroring the invasion of HSat1A, the enrichment of SST1 on the acrocentrics rises from 11% in the orangutans to 73% in gorilla, 81% in chimp, and 45% in human (Supplementary Table S2, Figure 1D). Subfamily SF1 is exclusively found on the ancestral acrocentrics of all species except orangutan, and, if acting as a hotspot for structural variation, its absence may explain the relative stability and uniformity of the orangutan acrocentrics (Figure 1C, Supplementary Figs. S1–S2).

### Karyotype evolution through short-arm inversion and NOR loss

**Figure 2.**
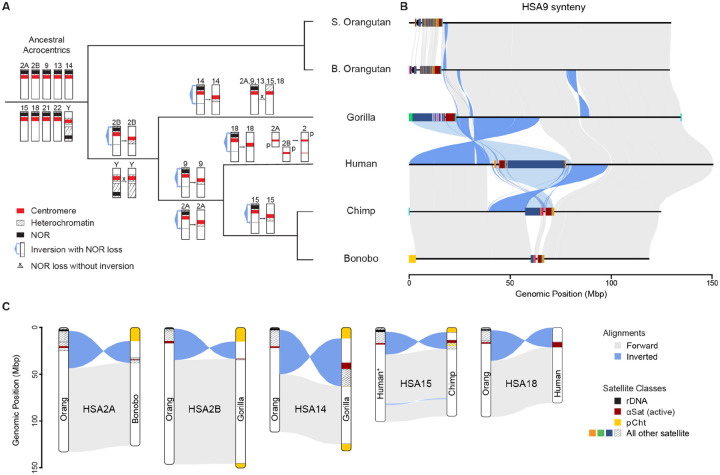
Inversion, fusion, and NOR loss events in the evolution of great apes. **(A)** Great ape phylogeny overlaid with the most parsimonious assignment of karyotype changes labeled on specific branches. **(B)** An example alignment of HSA9 across the six species studied here, showing acrocentric chromosomes in both orangutans and gorilla; conversion of acrocentric to metacentric in human, chimp, and bonobo; and a pericentromeric inversion between human and chimp/bonobo. The lighter blue highlights indicate the inferred repositioning of satellites that are too divergent to be reliably aligned, e.g. the repositioning of a large HSat3 block relative to the αSat centromere. **(C)** Simplified alignment schematics showing the five inversion events against an orangutan reference, with the exception of HSA15 shown against the human reference due to other complicating paracentric inversions relative to orangutan. pCht is a subterminal satellite cap that arose independently in the gorilla and Pan lineages^54^. Complete alignments for all events are given in Supplementary Figs. S10–S19.

Confirming prior hypotheses from FISH and cytogenetics^41^, pairwise alignments of the complete great ape chromosomes reveal at least six large-scale inversion events have converted acrocentrics to metacentrics. Assuming parsimony, these events can be placed on specific branches of the phylogeny (Figure 2A). Beginning with the oldest, the short arm of HSA2B was inverted in the human/gorilla common ancestor, followed by inversions of both HSA 2A and 9 in the human/chimp ancestor. Additional lineage-specific inversions have since occurred on the human branch (HSA18), the chimp/bonobo branch (HSA15), and the gorilla branch (HSA14). It is unclear whether these inversion events included the NOR, but inverted or proximal NORs appear to be selected against in the great apes given their uniform direction and location. Thus, since HSA 2A and 2B were both inverted prior to the human branch, they were likely NOR− at the time, which supports the hypothesis of a telomeric or subtelomeric fusion rather than a Robertsonian^8,53^.

Each short arm inversion was mediated by a break in the long arm of the ancestral chromosome, followed by a whole-arm inversion that repositioned the centromere and short-arm heterochromatin proximally. In the case of HSA9, the heterochromatin of the ancestral short arm was flipped into the middle of the chromosome, including a large HSat3 block and other acrocentric-specific repeats such as SST1 (Figure 2B). Although the satellite sequences have rapidly diverged due to sequence turnover and do not easily align, the location of the HSat3 block relative to the αSat centromere has swapped from gorilla to human, consistent with the inversion. The new centromere of HSA9 remains highly polymorphic in human, with heteromorphisms such as 9qh+ and inv(9)(p11q13) present in more than 1% of the population with unclear health implications^55^. A similar centromeric inversion appears in both chimp and bonobo genomes, and may also be polymorphic in their populations. This pattern of acrocentric loss by short-arm inversion is consistent across the other chromosomes, with the q-arm breakpoints localized within euchromatin approximately 10–40 Mb from the modern orangutan centromeres (Figure 2C, Supplementary Figs. S10–S19). Given the high rate of turnover on the short arms, the p-arm breakpoints are more difficult to pinpoint, and the amount of acrocentric-like sequence preserved in the pericentromere varies considerably.

In addition to short arm inversions, there have been multiple in-place NOR losses, including from chromosome Y on the human/gorilla/chimp branch, and from HSA 2A, 9, 13, 15, and 18 on the gorilla branch. Transient NOR loss appears relatively common in the great apes, given that three heterozygous NOR losses were observed in these six genomes alone^10^, including HSA13 of Sumatran orangutan (near-complete short arm loss), HSA18 of Bornean orangutan (distal truncation), and HSA21 of bonobo (distal truncation) (Figure 1C). Thus, it is possible that short arm truncation and NOR loss could have preceded the inversions, which would explain the smaller blocks of heterochromatin found in inverted HSA2B in gorilla and HSA18 in human.

The mechanism of NOR gain and loss on ChrY is more difficult to ascertain, given its high structural variability and rapid evolution among the great apes^13,31,56^. However, both orangutan and siamang Y chromosomes contain active NORs, suggesting a NOR+ ChrY in the ape common ancestor. These NORs always appear towards the distal end of the chromosome, making them prone to truncations without a substantial loss of additional ChrY sequence. Although no ChrY NORs remain in the other great apes, remnants of rRNA pseudogenes can be found near the ChrY centromere in human (Supplementary Fig. S20) and the DJ palindrome is remarkably amplified on the Y chromosomes of chimp and bonobo alongside the Y-specific genes *TSPY* and *RBMY* (Supplementary Fig. S21).

### Pseudo-homolog regions on the short arms

The complete T2T-CHM13 human genome revealed high degrees of similarity between all pairs of acrocentric chromosomes^12^, which were later confirmed to be recombining in the human population and termed PHRs^34^. This exchange maintains >99% sequence identity between the PHRs, well above what would be expected between heterologous chromosomes, and this high degree of interchromosomal similarity can be used as a surrogate for their detection in species that lack the population-scale long-read data needed to detect ongoing recombination.

**Figure 3.**
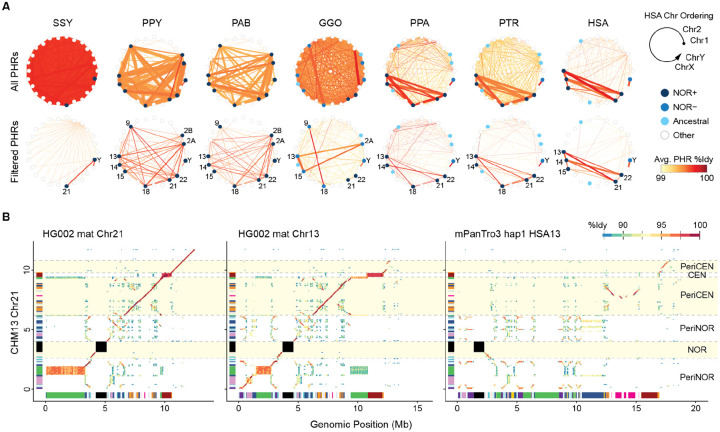
Substrates for recombination between heterologous acrocentrics. **(A)** Putative PHRs identified by sequence similarity between pairs of chromosomes, where the thickness of the line indicates the number of shared bases (max 15 Mb) and color indicates the average identity (99–100%). Values are the average of the diploid pairs. Top row includes all identified similarities. Bottom row excludes rDNAs, satellites, and subterminal regions, leaving only segmentally duplicated sequences. The acrocentric chromosomes for each species are numbered. **(B)** Percent identity dotplots between different human chromosomes illustrate a large, high-identity PHR between human Chrs 13 and 21 with residual similarity to chimp HSA13. The corresponding satellite annotations from Figure 1 are displayed on the X and Y axes. The centromere, NOR, and their surrounding regions are highlighted with respect to CHM13 Chr21.

Using a minimum threshold of 99% sequence identity over a 10 kb span, we found evidence of PHRs across the short arms of all great ape acrocentrics (Figure 3A). Considering all sequence classes, there is an average of ~186.5 Mb of identified PHRs per haplotype in each species, and the largest sum of PHRs between a single pair of chromosomes is 9.8 Mb (Bornean orangutan HSA2A and HSA22). The NORs are the most highly conserved PHRs among all species, including the rDNA array and DJ sequence, which share >99% identity across the apes compared to 97–99% for the entire short arm. Aside from the rDNAs, most PHR bases (115.5 Mb of the total 186.5 Mb) are dominated by satellites, such as the similar short arms of the orangutans, and the subterminal satellite regions of chimp, bonobo, gorilla, and siamang. After filtering rDNAs, satellites, and subtelomeric regions to focus on potentially genic regions, ~40.6 Mb of segmentally duplicated PHR sequence remains and distinct relationships between certain acrocentrics becomes clear (Figure 3A).

Certain pairs of chromosomes show stronger PHR relationships, such as Chr13 and Chr21 in human HG002, which share an average of 4.6 Mb per haplotype at 99.7% identity. Alignment of human Chr13 and Chr21 from HG002 to the CHM13 Chr21 reference shows a high degree of similarity extending nearly 3 Mb into the p-arm pericentric region in all three chromosomes, before becoming highly shuffled in the proximal region adjacent to the NOR (Figure 3B). This is the same PHR involved with Robertsonian chromosomes, noted above. The distal satellite region (distal PeriNOR) of CHM13 Chr21 is also a better match to HG002 Chr13 than Chr21, suggesting that the short arms of these chromosomes may interchange. These distal regions can be difficult to assemble, but their correct placement in CHM13 and HG002 has been previously validated by FISH^12,57^. Ectopic recombination between the pericentric PHRs of human chromosomes 13 and 21 was also observed in a multi-generation pedigree^58^.

Given the rapid evolution and high degree of turnover in the acrocentrics, the sequence and location of PHRs are not well conserved between species and synteny quickly breaks down in the short arms between species (Supplementary Figs. S10–S19). As noted previously, the NOR and DJ are inverted in chimp relative to human and surrounded by similar (but shuffled) classes of satellites and segmentally duplicated PJ sequences. In both species, the patterns of segmental duplication appear primarily organized around the NOR and centromere, with some local similarity remaining between species (Figure 3B). For example, >100 kb fragments of the pericentric PHR on human Chr13/21 can also be found on chimp and gorilla HSA13 with a gap-excluded nucleotide identity as high as 98%, similar to the genome-wide average. In human and chimp, this PHR contains mostly noncoding and pseudogene annotations, but in gorilla, multiple genes within this PHR appear to be protein coding, as detailed below.

All great ape species show similar, segmentally duplicated PHRs between all pairs of NOR+ acrocentric chromosomes, likely reflecting increased rates of sequence exchange due to their colocalization at the nucleolus. This includes the distal satellites, which tend to have a similar structure within each species (Figure 1C). ChrX and ChrY also consistently show a PHR relationship due to their pseudoautosomal regions (PARs), but the total amount of PAR sequence is often less than the acrocentric PHRs (Supplementary Table S3). Excluding subterminal satellites, the ancestrally acrocentric chromosomes tend to lose their PHRs, indicating a cessation of sequence exchange once they have converted to metacentric chromosomes (Figure 3A). In comparison, the gorilla NOR− acrocentrics contain even more PHR sequence than the NOR+ acrocentrics, suggesting that a NOR is not necessary to maintain recombination between short arms. In gorilla, three distinct acrocentric groups emerge from the PHR analysis: HSA2A/13/15 (NOR−), HSA9/18 (NOR−), and HSA21/22 (NOR+).

### Amplified protein-coding genes on the short arms of gorilla acrocentrics

**Figure 4.**
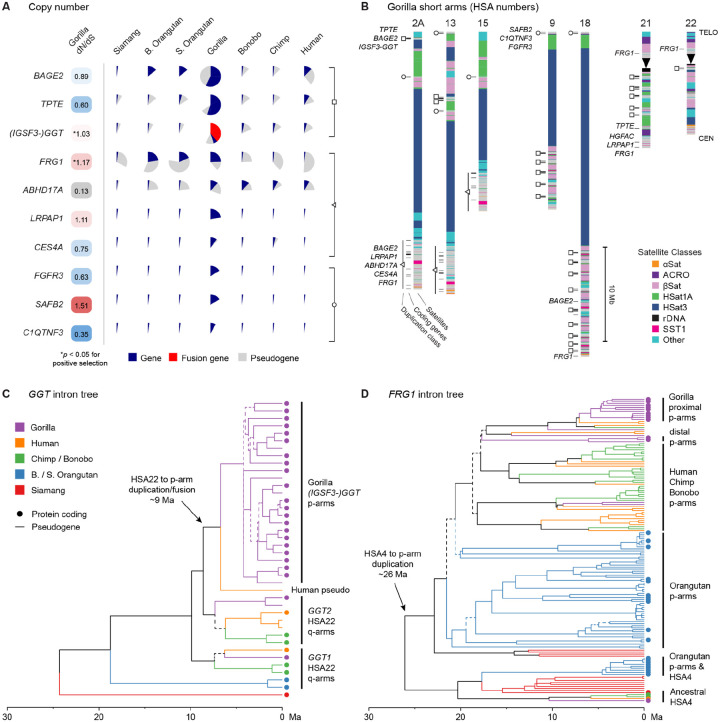
Amplification and selection of genes on the NOR− gorilla acrocentrics. **(A)** Copy numbers of protein-coding genes that have been amplified across the gorilla acrocentrics (primary haplotype), along with dN/dS values computed from paralogous coding copies. Three recurring segmentally duplicated gene clusters are indicated by a square, triangle, and circle. Although four genes have a dN/dS > 1, only *GGT* and *FRG1* were significant for positive selection with p < 0.05 after multiple testing correction. Selection tests were run using BUSTED on gap-trimmed alignments that excluded the *IGSF3* portion of *IGSF3-GGT* in gorilla and multiple variable exons of *ABHD17A* (gray). **(B)** Satellite and protein-coding gene annotations for the short arms of seven gorilla acrocentrics (primary haplotype, excluding ChrY), with recurring gene clusters summarized by symbols. **(C)** Time-measured intron phylogeny of all *GGT* genes including the *GGT* portion of the *IGSF3-GGT* fusion gene. **(D)** Time-measured intron phylogeny of all *FRG1* genes. For both trees, branches with a bootstrap <90% are dashed, and marmoset and macaque were used as outgroups (not shown). Fully labeled trees, including bootstraps and split times, are given in Supplementary Figs. S36 and S38.

Unlike human, annotation of the gorilla short arms revealed at least 10 predicted protein-coding gene families, particularly on the NOR− acrocentrics (Figure 4A, Supplementary Table S4–S5). Most of these genes are uniquely amplified in gorilla, confirming earlier array-based comparative genomic hybridization^59^ and FISH results^60,61^. The complete gorilla genome allowed us to pinpoint the location and composition of these amplifications and confirm which genes maintain open reading frames (ORFs) and are expressed (Supplementary Table S6). They can be roughly organized into three segmentally duplicated gene clusters (Figure 4A), all of which contain at least one gene family with a dN/dS > 1 (ratio of nonsynonymous substitutions per nonsynonymous site) and two of which contain a gene family with evidence of positive selection p < 0.05 using Branch-Site Unrestricted Statistical Test for Episodic Diversification (BUSTED)^62^ (Supplementary Table S7). Each cluster also contains additional coding genes that have a low fraction of pseudogenized copies and dN/dS values < 1, indicating they may remain under weak purifying selection (e.g. *TPTE* has 32 copies and only 5 pseudogenes). The prospect of gene conversion between copies possibly confounds our phylogenetic and positive selection analyses, but the variable branch lengths and dN/dS of adjacent coding sequences (CDS) within the same cluster also support differential selection (Supplementary Figs. S22–S34).

The locations of these amplified gene clusters correspond with similarly organized satellite structures on the gorilla acrocentrics and explain the observed PHR relationships between groups HSA2A/13/15, HSA9/18, and HSA21/22 (Figure 4B). The NOR− groups HSA2A/13/15 and HSA9/18 appear very self-similar, as do the distal ends of NOR+ HSA21/22, but the proximal regions of HSA21/22 are more distinct. Neither NOR+ acrocentric contains the large HSat3 arrays characteristic of the NOR− acrocentrics, as previously noted^41^, and these HSat3 arrays on HSA2A/13/15 are consistently inverted relative to those on HSA9/18. The observed patterns of amplification for all acrocentrics are consistent across both haplotypes, with minor variation in array sizes (Figure 1C).

The largest amplification primarily involves HSA9/18, which share 18.1 Mb of PHR sequence at 99.6% identity (7.6 Mb after satellite filtering), and includes a novel gorilla-specific fusion gene between *IGSF3* (Immunoglobulin Superfamily Member 3) and a member of the *GGT* (Gamma-Glutamyltransferase) family. GGT enzymes participate in glutathione metabolism and detoxification, potentially conferring an adaptive advantage for a diet rich in plant secondary metabolites^59^. The primary gorilla haplotype contains 21 *IGSF3-GGT* fusion genes that are predicted to be coding (average length 1,307 AA), along with fragments of *TPTE* (268 AA) and *BAGE2* (B Melanoma Antigen 2) (129 AA) that also maintain ORFs but in a modified structure compared to their human homologs. Of these genes, the *GGT* portion of *IGSF3*-*GGT* displays strong evidence of positive selection (BUSTED corrected p-value 1.38×10^−5, dN/dS 1.03), which could be driving the amplification. We focused on this half of the fusion gene because all but the first *GGT* exon are included in all copies (Supplementary Fig. S35), while the *IGSF3* portion is partial and does not show evidence of positive selection.

*GGT* is a multi-copy gene family in the great apes, with all species having at least two ORFs on HSA22, commonly referred to as *GGT1* and *GGT2*. Human, chimp, and bonobo also have multiple *GGT* pseudogenes (6, 5, and 7 copies, respectively), mostly occurring near the coding copies on the long arm of HSA22 (Supplementary Table S4). All *IGSF3-GGT* copies in gorilla are exclusive to the short arms of its acrocentrics, and human contains a single *IGSF3-GGT* fusion pseudogene in the short-arm pericentromere of human Chr13 next to *TPTE*. Thus, the fusion gene likely arose via a duplication onto the short arms in a common ancestor of human and gorilla. This hypothesis is supported by a phylogeny of *GGT* intron sequences that places the initial duplication of *GGT2* onto the short arms 7.2–10.0 million years ago (Ma) (Figure 4C, Supplementary Fig. S36), around the estimated split between human and gorilla 10.6–10.9 Ma^10^. Amplification across the acrocentrics proceeded sometime thereafter with all gorilla fusion copies coalescing 3.5–5.0 Ma. A corresponding CDS tree for *GGT* shows accelerated evolution in gorilla after formation of the fusion gene, especially for the HSA9/18 copies, presumably aided by recombination within and between the PHRs (Supplementary Fig. S28). The presence of this *GGT*-linked amplification, especially on HSA9/18 and including ChrY, was confirmed by FISH for nine different gorilla individuals (Supplementary Fig. S37–39, Supplementary Table S8).

A similar story of amplification tied to positive selection emerges for the pericentromeres of HSA2A/13/15 that total 57.5 Mb of shared PHR sequence at 99.5% identity (41 Mb after satellite filtering). This cluster harbors multiple predicted protein-coding genes including *FRG1*, which is critical for muscular and vascular development^63^. Gorilla contains 12 predicted protein-coding *FRG1* copies on the primary haplotype, while Bornean orangutan has 11, and Sumatran orangutan has 9. Both gorilla and orangutan amplified *FRG1* copies show evidence of accelerated evolution and positive selection of their CDS (Supplementary Fig. S27, BUSTED corrected p-values 1.88×10^−2 and 5.15×10^−8, dN/dS 1.169 and 1.018, respectively). Most of the pericentric copies on gorilla HSA2A/13/15 use an alternative start position, downstream of the canonical start (Supplementary Fig. S40), and their CDS cluster separately from the distal copies on HSA21/22 (Supplementary Fig. S27). Other amplified genes in this cluster include *ABHD17A* (Abhydrolase Domain Containing 17A, Depalmitoylase), *LRPAP1* (LDL Receptor Related Protein Associated Protein 1), and *CES4A* (Carboxylesterase 4A), with both *ABHD17A* and *LRPAP1* exhibiting modified exon structures that could be indicative of positive selection but could not be tested with BUSTED due to the large variation in gene models. Separate from the larger cluster, individual *LRPAP1*, *FRG1*, and *HGFAC* (HGF Activator) genes, all deriving from ancestral copies on HSA4, are found in the pericentromere of HSA21, again pointing to an initial duplication onto the acrocentrics preceding amplification. The presence of this *LRPAP1*-linked amplification across HSA2A/13/15 was also confirmed by FISH, again including some weak signal on ChrY (Supplementary Fig. S41–S42).

All apes contain a large number of *FRG1* genes or pseudogenes (12–39 copies per haplotype), pointing to an ancestral amplification^64^. However, in the human and chimp lineages, all but the ancestral copy on HSA4 have been pseudogenized by nonsense mutations. A phylogeny of *FRG1* intron sequences places an initial duplication of the ancestral HSA4 sequence onto the short arms of the acrocentrics 24.8–27.4 Ma in the ancestor of all apes, from where it repeatedly amplified and diversified (Figure 4D, Supplementary Fig. S43). All additional *FRG1* copies in human, chimp, bonobo, and gorilla derive from this ancestral duplication onto the acrocentrics, and do not cluster cleanly by species, indicative of amplification in their common ancestor. These copies coalesce 16.8–20.9 Ma, corresponding with the currently estimated human/orangutan split 18.2–19.6 Ma^10^. Orangutan and siamang also show evidence of this original duplication/amplification, as well as an independent and more recent amplification of the HSA4 copy, which has spread across the distal ends of the orangutan short arms. Thus, the mechanisms of interchromosomal exchange appear flexible and include both distal and proximal PHRs on both NOR+ and NOR– acrocentrics.

Lastly, a small, amplified cluster of three genes is found towards the distal end of all gorilla NOR− acrocentrics. The cluster always includes *SAFB2* (Scaffold Attachment Factor B2) and *FGFR3* (Fibroblast Growth Factor Receptor 3), with the third gene alternating between *C1QTNF3* (C1q And TNF Related 3) or *LETM1* (Leucine Zipper And EF-Hand Containing Transmembrane Protein 1). *SAFB2* has been associated with androgen receptor activity and male reproduction^65^, while *FGFR3* is a fibroblast growth factor receptor involved in skeletal development^66^. *C1QTNF3* occurs exclusively within the clusters on the very distal ends of HSA13 and HSA9/18, while *LETM1* is restricted to spacer sequence between HSat1A and βSat on HSA2A/13/15 and displays an atypical exon structure, leaving its coding status unclear. *SAFB2* maintains a modified ORF with a high dN/dS of 1.51 but does not show significant evidence for positive selection with BUSTED, whereas *FGFR3* and *C1QTNF3* have dN/dS values of 0.63 and 0.35, respectively, consistent with possible purifying selection.

### Structure of the gorilla *IGSF3-GGT* amplification

**Figure 5.**
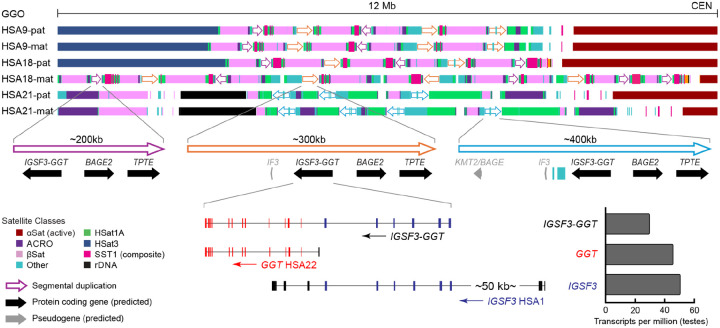
Structure of the amplified, gorilla-specific *IGSF3-GGT* gene cluster. Pericentric satellites and segmental duplications on the short arm of gorilla chromosomes HSA 9, 18, and 21, illustrating amplification of the *IGSF3-GGT* gene cluster. The large blocks of “Other” satellites are mostly composites of HSMAR2-like repeats. Gene structures are shown for *IGSF3*, *GGT*, and the *IGSF3-GGT* fusion. The histogram to the right of each model shows the TPM values for each gene model in gorilla testes.

As noted above, the *IGSF3-GGT* fusion gene likely arose in a common ancestor of human and gorilla by duplication of the *IGSF3* and *GGT2* genes onto the short arms of the acrocentrics, from which it was amplified and maintained by positive selection in the gorilla lineage. To better understand how this new gene duplicated and evolved in concert, we investigated the structure of the amplifications. In particular, gorilla HSA 9, 18, and 21 contain arrays of tandemly repeating, and sometimes inverted, copies of the *IGSF3-GGT* cluster alternating with the acrocentric-associated satellites βSat, HSat1A, ACRO, and SST1, as well as composite repeat arrays with homology to the human mariner transposon, HSMAR2 (Figure 5). The core of the segmental duplication, including *IGSF3-GGT*, *BAGE2*, and *TPTE*, is conserved across all three amplified arrays, but the longest duplication on HSA21 also includes an additional *BAGE2* pseudogene fragment.

The *IGSF3-GGT* fusion gene structure is preserved across all copies, beginning with six internal exons of *IGSF3* and ending with all but the first exon of *GGT*. Long read transcripts from testes show similar expression levels for the individual *IGSF3* and *GGT* genes, and strong, but slightly lower, expression of the fusion gene measured in transcripts per million (TPM). Absence of *GGT* and *IGSF3-GGT* transcription in gorilla fibroblasts is consistent with *GGT* expression patterns in human, where it is most strongly expressed in kidney and intestine^67^. Together, the shared satellite patterns, expression evidence, *GGT* phylogeny, and positive selection evidence suggest that the *IGSF3-GGT* copies are functional and evolving in concert.

**Figure 6.**
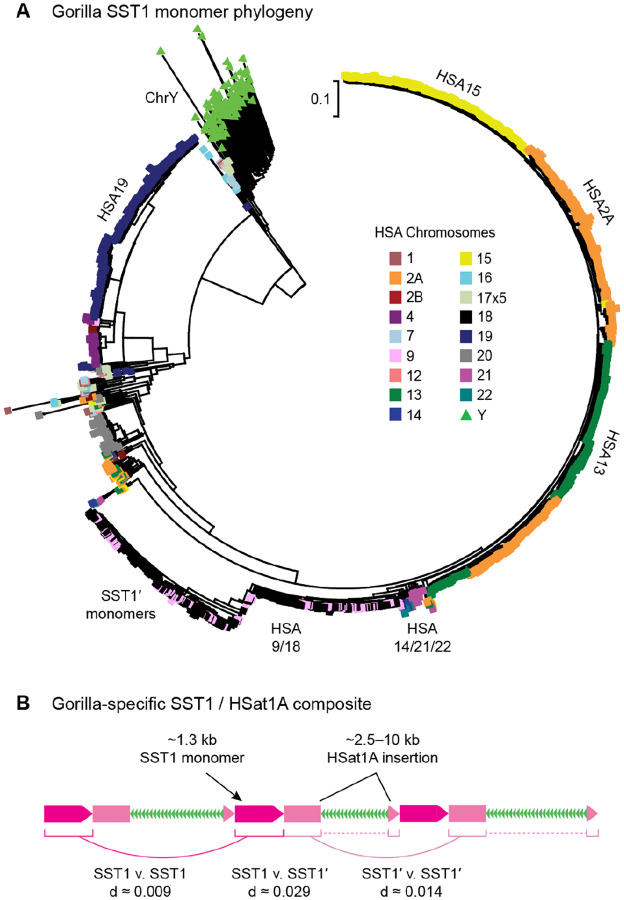
**(A)** Phylogenetic relationship of 3,078 full-length SST1 monomers in gorilla with color indicating the source chromosome. All SST1 copies on the acrocentric chromosomes are separated from all other chromosomes by a single branch, with the exception of HSA14, which was only recently converted to a metacentric. SST1 repeats from HSA9 (pink) and HSA18 (black) co-cluster, consistent with frequent recombinational exchange. A subclade of SST1′ monomers exclusive to HSA9/18 shows accelerated evolution. **(B)** Schematic representation of the SST1 composite found on gorilla chromosomes HSA9/18, where the arrays are organized as tandemly arranged dimers of SST1 (magenta) and SST1′ (pink) with HSat1A insertions (green). Average nucleotide distances are given between SST1 and SST1′ copies showing the increased divergence of SST1′ copies. Differential clustering of SST1 and SST1′ monomers in the phylogeny suggests the SST1′ copies are evolving as dimers.

To further understand the patterns of sequence exchange in the gorilla PHRs that could result in concerted evolution, we focused on the SST1 macrosatellite that has been previously implicated as a human translocation hotspot^21^. Not only has SST1 been previously implicated in similar recombination events in primates, but it is also a convenient marker of interchromosomal exchange because its arrays are composed of well-defined ~1.4 kb monomers that are found on all gorilla acrocentrics and the pericentromeres of many other chromosomes. Both the gorilla HSA9/18 and HSA2A/13/15 PHRs contain prominent SST1 arrays. A phylogeny of all SST1 monomers in the gorilla genome mirrors the observed PHR associations, with HSA9/18 and HSA2A/13/15 co-clustering, as expected (Figure 6A). In particular, the HSA9/18 monomers show the highest similarity between chromosomes and co-cluster within the same clade, indicative of more recent exchange between these two chromosomes. Pericentromeric HSA21 SST1 monomers are also highly similar to HSA9/18 monomers, but branch ancestrally in the tree. This is consistent with HSA21 *GGT* CDS copies, which also branch ancestrally from the HSA9/18 copies (Supplementary Fig. S28). Closer inspection reveals that the HSA9/18 SST1 monomers comprise a unique composite (SST1′) of SST1 and HSat1A elements that is only found on these two chromosomes and localized to the *IGSF3-GGT* amplified PHR (Figure 6B). Excluding the HSat1A insertions, the monomeric SST1′ sequence itself shows an elevated substitution rate compared to SST1. Overall, this pattern of diversification matches that of *IGSF3-GGT* and suggests that accelerated exchange between the HSA9/18 PHRs may be promoted by novel satellites such as SST1′.

The satellite patterns, predicted PHRs, and phylogenies all suggest that the gorilla NOR− acrocentrics independently associate and exchange with one another. For example, although HSA21 contains an array of *IGSF3-GGT* genes, they are arranged in a unique structure compared to HSA9/18 and cluster separately in both the CDS and intron phylogenies. This is consistent with our observation that, although the NOR− acrocentrics contain large satellite blocks, they do not reliably associate with nucleoli (Supplementary Fig. S44). Thus, interchromosomal exchange is not dependent on the presence of NORs but may be a more general feature of repetitive, homologous sequences found on the short arms of acrocentric chromosomes combined with mechanisms associated with non-allelic homologous recombination (NAHR). Interestingly, the three groups of acrocentric chromosomes in gorilla each have their own unique satellite organization. For example, the large HSat3 arrays on HSA9/18 and HSA2A/13/15 are in a common orientation within each group, but opposite orientations between the groups, and HSA21/22 contain very little HSat3. Further supporting a cessation of recombination after conversion to a metacentric, SST1 monomers on ancient acrocentrics, such as HSA2B, cluster ancestrally to copies on modern acrocentrics.

## Discussion

Complete sequencing of the great apes has revealed the most rapidly evolving regions of their genomes, including recently duplicated sequences that are closely linked to adaptation and speciation^10^. We have shown here that the short arms of the acrocentric chromosomes are enriched for such duplications and have been key drivers of karyotype evolution and genomic innovation in the great apes. Thus, the acrocentric chromosomes have played a critical role in the past adaptation of our ancestors and continue to play an important role in our health today.

At least one whole short-arm inversion event can be placed on each major branch of the species tree, converting acrocentric chromosomes to metacentric chromosomes and forming distinct karyotypes for each species. This fits the hypothesis that pericentric inversions are statistically directional, i.e. they favor the conversion of acrocentric to metacentric chromosomes^68^, which may then become fixed in the population through selection, meiotic drive, or genetic drift^39^. The result is a progressive loss of acrocentrics in the great apes, and a trend towards metacentrics compared to the ancestral karyotype. Notably, the two species that have experienced the fewest acrocentric to metacentric conversions, orangutan and gorilla, both harbor positively selected genes on their short arms (e.g. *FRG1*), potentially countering the forces leading to acrocentric loss in the other species.

Further, we have shown that the unique compositional and recombinational properties of the short arms, including satellite DNAs and PHRs, lend themselves not only to the homogenization of rDNA arrays but also the amplification of satellites and multicopy gene families through birth-and-death evolution, whereby new sequences arise through duplication and are maintained or lost based on selective pressures^69^. The short arms of the acrocentrics appear especially tolerant of genomic repeats, perhaps due to a suppression of meiotic recombination^58^, similar to what is observed within centromeres and the non-recombining region of ChrY. However, enough sequence exchange remains between the short arms to facilitate sequence amplification and concerted evolution within PHRs. Our results suggest that many of the pericentromeric repeats observed across the human genome, such as the HSat2,3 arrays, were first seeded and amplified on the short arms of acrocentric chromosomes before being moved proximally by a chromosomal fusion or inversion. This may include Chr16, whose ancient conversion to metacentric and subsequent cessation of sequence exchange with the acrocentrics could explain the divergence of its pericentromeric HSat2 array.

We have primarily focused on the evolution of acrocentric chromosomes following the human and orangutan split ~19 Ma, but not the events leading to their emergence in the great ape ancestor. This is a more complicated picture involving multiple chromosome fission and fusion events, and only Chr13 and ChrY are acrocentric in both human and the inferred primate common ancestor^5^. The hypothesized ancestral primate karyotype contained as many as 14 acrocentric chromosomes^42^, many presumably carrying a NOR. Some of these ancient acrocentrics were lost by fusions, including HSA 10, 16, and 19, and others by short-arm inversions, like those presented here. In the Old World monkeys, *Macaca mulatta* lost all acrocentric NORs and now carries a single pericentromeric NOR resulting from the fusion of HSA20 and HSA22^41,70^. Conversely, new acrocentrics have been created by fission of the ancestral primate karyotype, including HSA14/15, HSA3/21, and HSA12/22^42,71^. The subsequent gain of NORs on these chromosomes points to a general tendency of acrocentric chromosomes to exchange and acquire NORs from each other, and a similar mechanism of NOR gain through centric fission and satellite expansion has been hypothesized in ants^72^. Thus, karyotype evolution by NOR-associated fission and fusion events may be a general mechanism contributing to eukaryotic speciation.

It is debatable whether the redundancy of multiple NORs, as seen in the great apes, is advantageous or simply a consequence of the different selective pressures acting on the short arms of acrocentric chromosomes. Dispersing onto multiple chromosomes may offer the benefit of redundancy and help balance overall rDNA copy number and fidelity, reducing the impact of deleterious variants, array silencing, or array loss^29^. Combined with strong purifying selection, the mechanisms of molecular drive (e.g. unequal crossing over) can maintain a high degree of similarity with individual rDNA arrays^33,35^. Selection appears to favor this form of redundancy on the ends of chromosomes, since crossover between pericentromeric arrays on two metacentrics would result in a deleterious translocation. This is consistent with the uniform orientation of rDNA arrays within each great ape species, and lone pericentric NOR in *M. mulatta*, thereby preventing crossover recombination of different rDNA arrays from forming a Robertsonian chromosome.

The gorillas illustrate that these mechanisms of redundancy and molecular drive are not limited to the rDNA arrays and can involve the entire short arms of NOR− chromosomes. The gorilla NOR− acrocentrics appear to have repurposed their remaining heterochromatin, in the form of satellite DNA, to facilitate recombinational exchange between the short arms of heterologous acrocentric chromosomes^73,74^. This has enabled the amplification and concerted evolution of protein-coding genes in a manner similar to the rRNA genes. Thus, given their ability to flexibly amplify and recombine all manner of genomic sequences with minimal fitness costs, the short arms of acrocentric chromosomes appear to be underappreciated hubs of genomic adaptation. Similar effects have also been reported within the subterminal heterochromatin caps of chimp and gorilla chromosomes^54^. As Ohno stated in summarizing the importance of duplications in evolution, “natural selection merely modified, while redundancy created”^75^. In this way, the redundancy generated by interchromosomal exchange between the acrocentrics generates additional variation for natural selection to act upon, and the degree of homogenization versus diversification can be fine-tuned by the rate of recombination. This manifests as both the highly homogenized nature of the tandemly repeating rRNA genes (high rates of exchange) and the segmentally duplicated PHRs (lower rates of exchange). For example, by decoupling from the nucleolus, the gorilla NOR− acrocentrics have been freed to fine-tune their own degree of concerted evolution. These PHRs will continue to evolve in concert until random mutation outpaces the rate of interchromosomal exchange and the process halts^76^, as seen with sequences on chromosomes that have converted from acrocentric to metacentric, thus, lowering their rate of recombination and allowing mutations to dominate.

The amplified gene families found on the gorilla acrocentrics obviously raise questions of adaptation, and these genes are broadly associated with common drivers of rapid evolution, including immunity and inflammation (*ABHD17A*, *C1QTNF3, GGT*, *SAFB2*), metabolism and detoxification (*C1QTNF3*, *CES4A*, *GGT*, *LRPAP1*), musculoskeletal development (*FGFR3*, *FRG1*), reproduction (*SAFB2*, *TPTE*), and cancer/testis genes (*BAGE2, TPTE*). In particular, *IGSF3-GGT* and *FRG1* show evidence of positive selection, and it is tempting to speculate how these unique genomic features may be linked to gorilla-specific traits such as their unique diet and sexual dimorphism. These two gene families also provide a beautifully contrasting view of birth-and-death evolution in the apes, with the newer *IGSF3-GGT* recently “born” in gorilla and the much older *FRG1* copies having pseudogenized outside of gorilla and orangutan. In human, remnants of these ancient *FRG1* amplifications remain as expressed pseudogenes on the acrocentrics^12^, and their functional effect (if any) has not yet been determined.

Here we investigated the unique features of great ape acrocentric chromosomes and found that, in some cases, selection appears to have driven the amplification of genes and repeats across the short arms, where they can be readily expanded across multiple chromosomes and recombined. Through a series of past fusions and inversions, some of these sequences have been repositioned into the pericentromeres of metacentric chromosomes, helping to explain the distribution of satellites and segmental duplications in modern genomes. We speculate that some of these remnants are now responsible for chromosome instability, such as variation in and around the pericentromeric heterochromatin of Chr9 and Chr16^77^ and Robertsonian chromosome formation^21^. For example, the Chr13/14/21 PHR thought to facilitate most Robertsonians in humans contains many *FRG1* pseudogenes that may have once been under positive selection. Thus, the same processes that orchestrated adaptation in our ancestors can also predispose us to certain genomic disorders; evolution is often a double-edged sword. More complete, T2T genomes are now needed, along with long-read functional genomics data, to enable a greater understanding of these previously “dark” regions of the genome and their role in adaptation and evolution across species.

## Methods

### Satellite and rDNA annotation

Satellite annotations were primarily drawn from prior work^10^, but a few gaps were identified in the original annotation. To fill these gaps, the genomes were additionally annotated using AniAnn’s (github.com/marbl/anianns, git commit 8dd4e1b). AniAnn’s identifies tandem repeats by splitting each sequence into windows (2,000 bp) and measuring the average nucleotide identity (ANI) between adjacent windows. Previously unannotated regions exceeding an ANI threshold of 86% were identified as unknown satellite arrays and added as “other” satellites to the original annotation.


anianns annotate -f $species.fa -c $kmer_dir > $species.bed 


All percent identity dotplots were created using ModDotPlot v0.9^78^.


moddotplot static -f $species.fa -a $max_length


To calculate satellite enrichment on the acrocentrics, the above satellite annotations were subsetted for various satellite classes and partitioned by species and chromosome type.

To show the orientations of rDNA arrays are consistent within species, each ape genome was mapped to the human 45S gene extracted from the KY962518.1 rDNA reference sequence^79^ using minimap2^80^. Mappings were inverted and filtered for a minimum of 95% identity and over a 10 kb alignment block to ensure accuracy and inspected in the UCSC Genome Browser^81^.


minimap2 -c -x asm20 --eqx --MD -t 2 45S.fa $species.fa > $species.to.45S.paf rustybam invert $species.to.45S.paf > 45S.to.$species.paf 
awk -v OFS=‘\t’ ‘{if($11>=10000 && $10/$11 >= 0.95) print $6,$8,$9, $10/$11}’ 45S.to.$species.paf > 45S.to.$species.filt.bed


The same was done for the DJ, aligning a representative copy from T2T-CHM13 Chr13 but filtering for 100 kb matches at 90% identity.


minimap2 -c -x asm20 --eqx --MD -t 2 DJ_palindrome.fa $species.fa > $species.to.DJ_palindrome.paf 
rustybam invert $species.to.DJ_palindrome.paf > DJ_palindrome.to.$species.paf awk -v OFS=‘\t’ ‘{if($11>=100000 && $10/$11 >= 0.9) print $6,$8,$9, $10/$11}’ DJ_palindrome.to.$species.paf > DJ_palindrome.to.$species.filt.bed


The above DJ alignment and filtering methods were also used to identify remnants of the DJ on the chimp and bonobo Y chromosomes.

### Pairwise alignment of whole chromosomes

Primary haplotypes for each chromosome belonging to the superset of great ape acrocentric chromosomes (HSA 2A, 2B, 9, 13, 14, 15, 18, 21, 22, Y) were extracted from each species and renamed to indicate the species of origin and HSA chromosome designation. HSA9 and Y were inverted in chimp and bonobo to be consistent with the other great apes. Whole chromosomes were aligned pairwise using wfmash (https://github.com/waveygang/wfmash, version 0.21.0) with default parameters, except for a 50 kb seed size to reduce spurious alignments in repetitive sequences.


wfmash -t 16 -s 50000 -p 85 $fa_2 $fa_1 > $out.paf


The resulting PAF files were concatenated to contain one of each species pair in a central PAF file for each homologous chromosome. The resulting alignments and corresponding satellite annotations were visualized using the AVA mode of SVbyEye^82^, showing large synteny blocks and inversions between pairs of species.

### Pseudo-homolog region identification

To identify PHRs, each chromosome was compared in a pairwise fashion with MashMap3^83^ to all other chromosomes using 10 kb chunks and an identity threshold of 85%. The resulting matches were filtered to only retain hits above 99% identity for the purpose of identifying putative PHRs. To generate heatmap tracks for viewing, the best MashMap hit to any position on the query chromosome was retained for each 10 kb window on the reference chromosome. A custom script was used to convert percent identity to an RGB value on the YlOrRd scale, ranging from 80–100%, with values below excluded and appearing white.

The mappings were then summarized to calculate the amount of shared sequence above 99% identity between all pairs of chromosomes in each species. Total bases and average percent identity of the shared sequence were averaged for each diploid pair of chromosomes to obtain haploid counts. A custom script using NetworkX^84^ was used to plot the interchromosomal relationships. The average percent identity of shared sequence between chromosomes was plotted as line color by filtering alignments for those above 99% identity, subtracting 99%, and normalizing between 0 and 1. The line width was normalized by dividing the total shared sequence by 2.5 Mb and using this value as the line width in pixels. Since the amount of shared sequence between chromosomes was dominated by satellite DNA, rDNA, and subterminal spacers in the pCHT arrays, these were filtered to isolate the segmental duplications.

The segmental duplication structure of the HSA9/18 and HSA21 amplifications was first assessed with dotplots and satellite annotations. This revealed three approximate gene clusters of varying lengths and orientation. One representative cluster was extracted for each class and realigned to the gorilla genome with minimap2 to identify all instances of each cluster aligning with at least 90% average identity over 100 kb.

### Amplified gene annotation

Initial gene annotations for the short arms of the gorilla acrocentrics were taken from NCBI RefSeq^85^ and Comparative Annotation Toolkit (CAT) annotation datasets^10^. Due to incompleteness in both count and transcript-level detail within the duplicated regions, we performed systematic manual curation of all genes appearing at least once on the short arms of the gorilla acrocentrics. For these genes, all potential paralogs were identified across the great apes using BLAT sequence alignment^86^ against the complete assemblies and visualized in the UCSC Genome Browser. Each paralog was manually assessed for protein-coding potential by considering exon structure integrity, nonsense mutations leading to pseudogenization, and transcriptional activity confirmation using long-read PacBio Iso-Seq transcripts from testes and fibroblast samples of the reference individual (KB3781). For each of the amplified gene families, TPM was calculated using IsoQuant^87^ for each sample type separately and combined. Reads were first aligned using minimap2, sorted and indexed with samtools, and IsoQuant was then run using default parameters and consensus NCBI/CAT gene annotations.

### Gene phylogenies and selection analysis

Protein-coding copies for each gene family were extracted and aligned using MAFFT v7.5 with default parameters^88^. Phylogenetic relationships were reconstructed using maximum likelihood methods implemented in RAxML-NG under a GTR+G+I substitution model with default settings^89^. Before conducting selection tests, any gaps in the CDS alignments were trimmed using MEGA to visualize and edit the alignments^90^. Gaps and novel exons were removed to ensure that only the core set of codons shared across all the copies were present in the alignment. Selection analysis was conducted using BUSTED^62^ to detect gene-wide episodic diversifying selection, implementing site-to-site synonymous rate variation and flexible random effects branch-site variation for dN/dS ratio estimation. A significance threshold of p < 0.05 was applied to all analyses after Bonferroni correction to account for the 14 tests performed.

To estimate the age of *FRG1* and *GGT* duplications, intronic sequences were extracted from both gene bodies and aligned with MAFFT using parameters:


--anysymbol --reorder --maxiterate 1000 --thread 16


The alignment was then processed with IQ-TREE2^91^ using parameters:

--date {divergence_time_file} --keep-ident --date-tip 0 --date-ci 100 -B 1000
-T 36 

to generate a maximum likelihood phylogeny that was bootstrapped using UFBoot2^92^ with 1,000 replicates and dated using LSD2^93^ with an estimated human/macaque divergence time of 28.8 Ma and human/marmoset divergence time of 43 Ma.

### SST1 monomer characterization

SST1 arrays were initially identified using RepeatMasker (http://www.repeatmasker.org). For each identified array, sequences were retrieved along with 1 kb of flanking sequence. All clusters were manually curated through visual inspection. This was performed by generating dotplots using the Dotlet applet^94^ with a word size of 15 bp and a similarity cut-off of 60%. The consensus sequences were refined through an iterative process, which enabled precise sequence characterization. This manual curation allowed for the definitive identification of the start and end points of each array and its constituent monomers relative to the generated consensus. To ensure consistency in subsequent alignments, all monomeric sequences were characterized using identical start and end points defined by the consensus. All retrieved full-length SST1 monomeric sequences from assembled genomes were aligned using MAFFT with the G-INS-1 model. A maximum likelihood phylogenetic analysis was conducted under the best-fit nucleotide substitution model (GTR +G, with a shape parameter α = 5.5047), as determined by jModelTest2^95^. The robustness of the inferred topology was assessed with 1,000 bootstrap replicates.

### Gorilla fluorescence in situ hybridization

Two human BAC clones were independently tested by FISH experiments in gorilla to confirm the primary amplifications were not isolated to the assembled individual: RP11–341D18 (AL356585.7), overlapping the *GGT-IGSF3* amplification, and RP11–529E10 (AL590235.23), overlapping the *LRPAP1* (*FRG1*) amplification.

Western lowland gorilla fibroblast cell lines were obtained from the San Diego Zoo Wildlife Alliance (KB3781), and from Coriell (AG20600, AG21765, and AG05251). Note, KB3781 “Jim” is the same animal originally used for the T2T reference assembly. All cell lines were initially cultured in alpha-MEM (Gibco) with 2mM L-glutamine supplemented with 15% fetal bovine serum (FBS) and then switched to DMEM/F12 (Gibco) with 15% FBS. Cell cultures were maintained in a 37°C incubator with 5% CO2. For the preparation of chromosome spreads, cells were blocked in mitosis by the addition of Karyomax colcemid solution (0.1 μg/ml, Life Technologies) for 6–7 h and collected by trypsinization. Collected cells were spun down and incubated in hypotonic 0.4% KCl solution for 12 min and prefixed by addition of methanol:acetic acid (3:1) fixative solution (1% total volume). Pre-fixed cells were spun down and then fixed in methanol:acetic acid (3:1). Chromosome spreads were dropped on a glass slide and incubated at 65°C overnight. Before hybridization, slides were treated with 0.1mg/ml RNAse A (Qiagen) in 2xSSC for 45 minutes at 37°C and dehydrated in a 70%, 80%, and 100% ethanol series for 2 minutes each. Slides were denatured in 70% deionized formamide/2X SSC solution pre-heated to 72°C for 1.5 min. Denaturation was stopped by immersing slides in 70%, 80%, and 100% ethanol series chilled to −20°C. Fluorescently and biotin-labeled BAC probes RP11–450E20 (rDNA), RP11–341D18 (*GGT*), and RP11–529E10 (*LRPAP1*) were obtained from Empire Genomics. Human Chromosome 21 paint was obtained from Applied Spectral Imaging. Probes used in combinations were denatured in a hybridization buffer by heating to 80°C for 7 minutes before applying to denatured slides. Specimens were hybridized to the probes under a HybriSlip hybridization cover (GRACE Biolabs) sealed with Cytobond (SciGene), in a humidified chamber at 37°C for 48 hours. After hybridization, slides were washed in 50% formamide/2X SSC 3 times for 5 minutes per wash at 45°C, then in 1x SSC solution at 45°C for 5 minutes twice, and at room temperature once. For biotin detection, slides were incubated with streptavidin conjugated to Cy5 (Thermo) for 2–3 h in PBS containing 0.1% Triton X-100 and 5% bovine serum albumin (BSA), and then washed 3 times for 5 minutes with PBS/0.1% Triton X-100. Slides were rinsed in water, air-dried in the dark, and mounted in Vectashield-containing DAPI (Vector Laboratories). Z-stack confocal images of chromosome spreads and interphase nuclei were acquired on the Nikon TiE microscope equipped with 100x objective NA 1.45, Yokogawa CSU-W1 spinning disk, and ORCA-Fusion BT C15440 CMOS camera (Hamamatsu). Image processing was performed in FIJI^96^.

FISH experiments with RP11–341D18 were independently replicated for additional *Gorilla gorilla gorilla* skin-derived cell lines from the Ventura lab at University of Bari (GGO, GGO1, GGO2, GGO5, GGO9, GGO14). Note, GGO14 is the same individual as AG20600 (above), but all others are unique individuals from European zoos; thus, a total of nine unique individuals were analyzed by FISH. For this preparation, all BACs were isolated using the PureLink Quick Plasmid Miniprep Kit (Invitrogen), labeled by nick translation with Cy3-dUTP, and subsequently subjected to ion-exchange alcohol precipitation in the presence of human Cot DNA^97^. Following denaturation at 70°C for 2 minutes, hybridization was performed overnight at 37°C. Post-hybridization washes were performed three times in 0.1× SSC at 60°C under high-stringency conditions. Slides were counterstained with DAPI, and fluorescence signals from Cy3 and DAPI were independently detected using a Leica DMRXA epifluorescence microscope equipped with a cooled CCD camera (Princeton Instruments). Images were acquired in grayscale, pseudo-colored, and merged using Adobe Photoshop. Signal intensity was recorded semi-quantitatively (+ to ++++) and localization noted.

## Resource Availability

### Lead contact

Adam M. Phillippy <adam.phillippy@nih.gov>

### Materials availability

No new materials were generated from this study.

### Data and code availability

Supplementary data including custom analysis code, self-identity heatmaps for all ape chromosomes, multiple-sequence alignments (CDS, introns, SST1), and satellite annotations are available from doi: 10.5281/zenodo.18022857

## Supplementary Material

Supplement 1

Supplement 2

Additional supplementary materials include:

Supplementary Figures S1–S44

Supplementary Tables S1–8
