# Supplementary material for: Origin and evolution of acrocentric chromosomes in human and great apes
Source: bioRxiv. 2025 Dec 23:2025.12.22.696095. Preprint. [Version 1] doi: 10.64898/2025.12.22.696095 (PMC12767296; doi:10.64898/2025.12.22.696095)
Supplement: Supplement 1 [file media-1.pdf]

Origin and evolution of acrocentric chromosomes in human and great apes

Supplementary Figures S1–S44

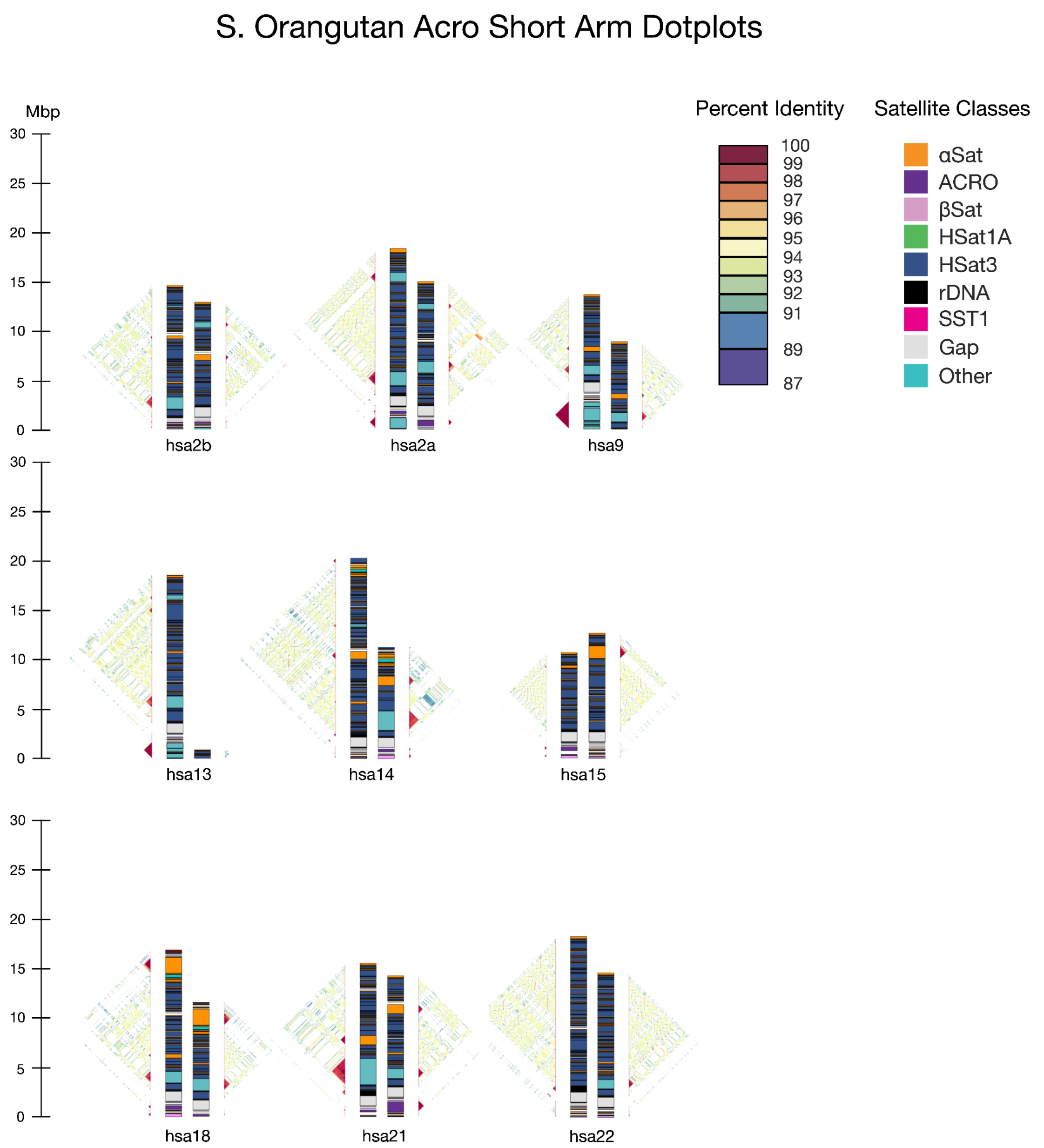

Figure S1. Self-alignment heatmaps for the short arms of the S. orangutan acrocentrics. ModDotPlot figures illustrate the differences in size and content between primary (left) and alternate (right) haplotypes. Corresponding satellite annotation tracks are provided in between each plot.

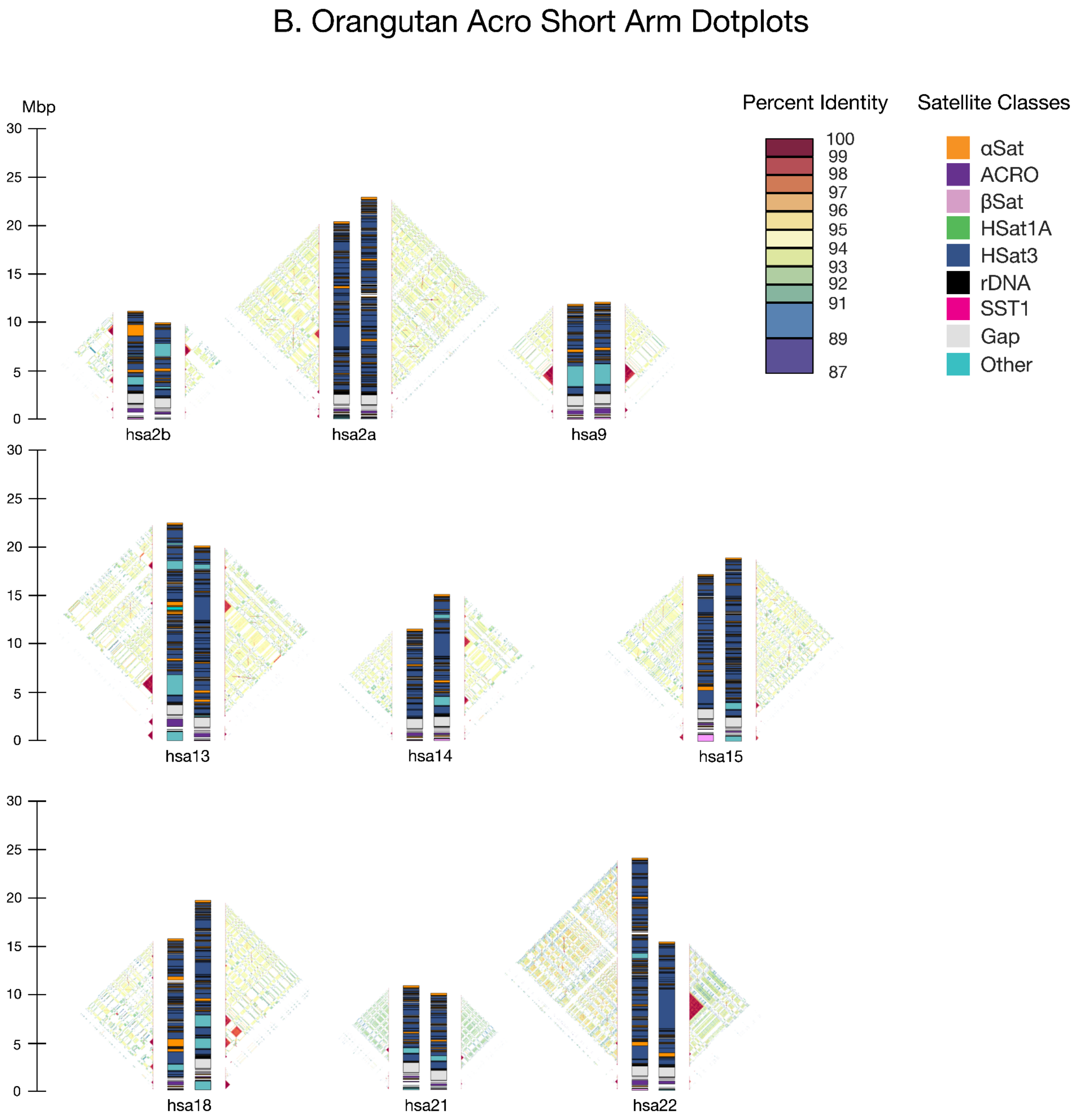

Figure S2. Self-alignment heatmaps for the short arms of the B. orangutan acrocentrics. ModDotPlot figures illustrate the differences in size and content between primary (left) and alternate (right) haplotypes. Corresponding satellite annotation tracks are provided in between each plot.

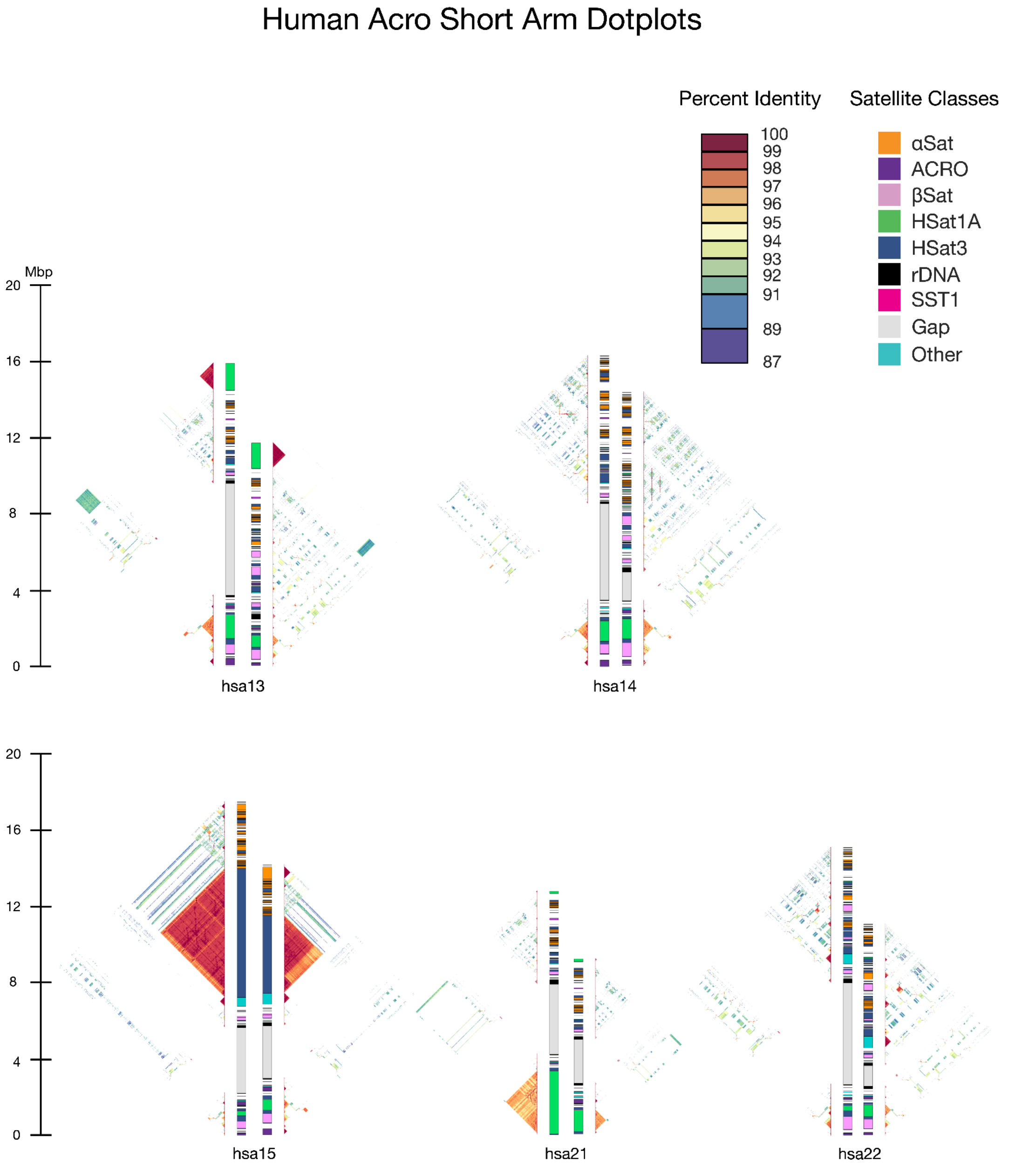

Figure S3. Self-alignment heatmaps for the short arms of the human (HG002) acrocentrics. ModDotPlot figures illustrate the differences in size and content between maternal (left) and paternal (right) haplotypes. Corresponding satellite annotation tracks are provided in between each plot.

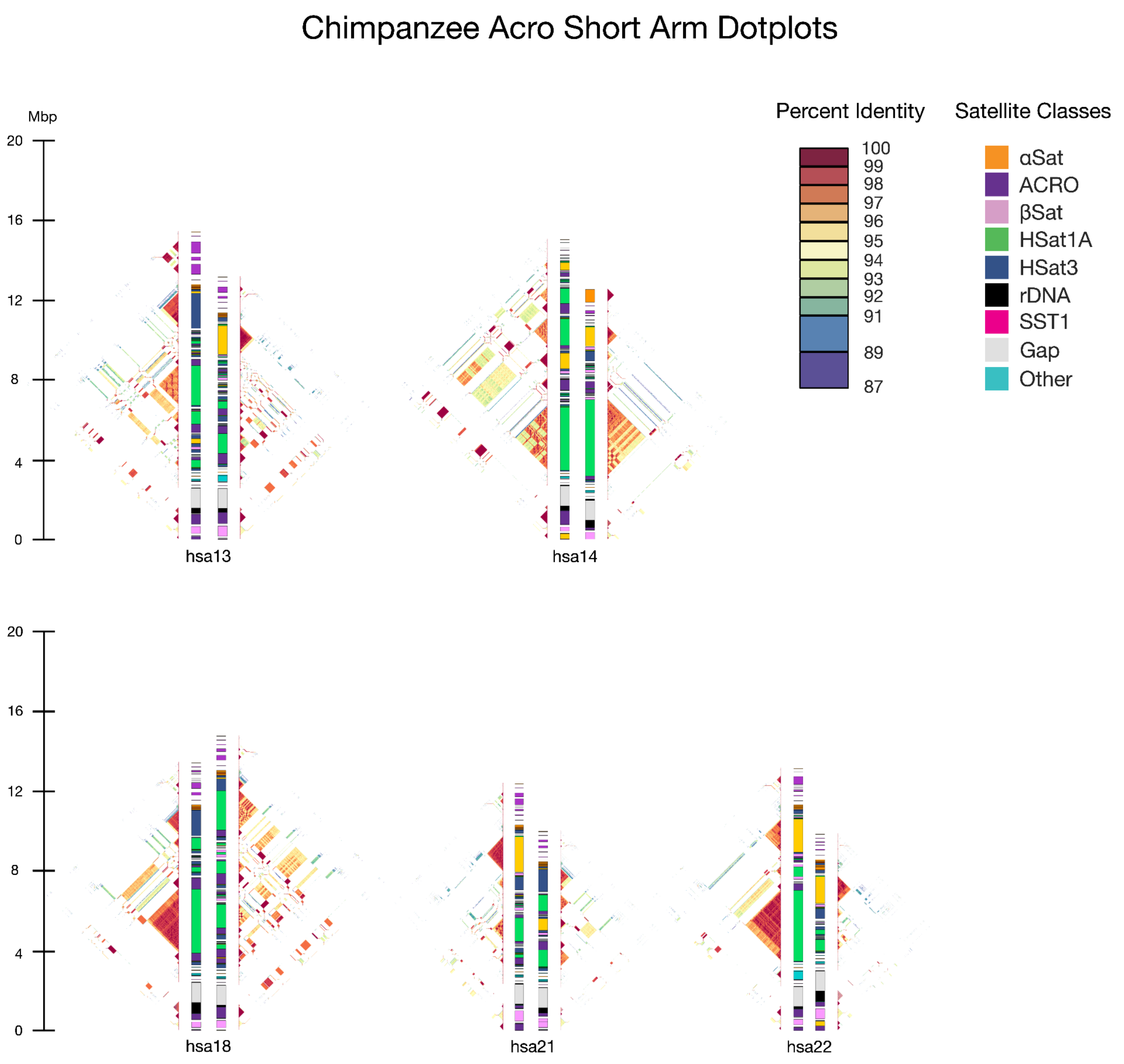

Figure S4. Self-alignment heatmaps for the short arms of the chimp acrocentrics. ModDotPlot figures illustrate the differences in size and content between primary (left) and alternate (right) haplotypes. Corresponding satellite annotation tracks are provided in between each plot.

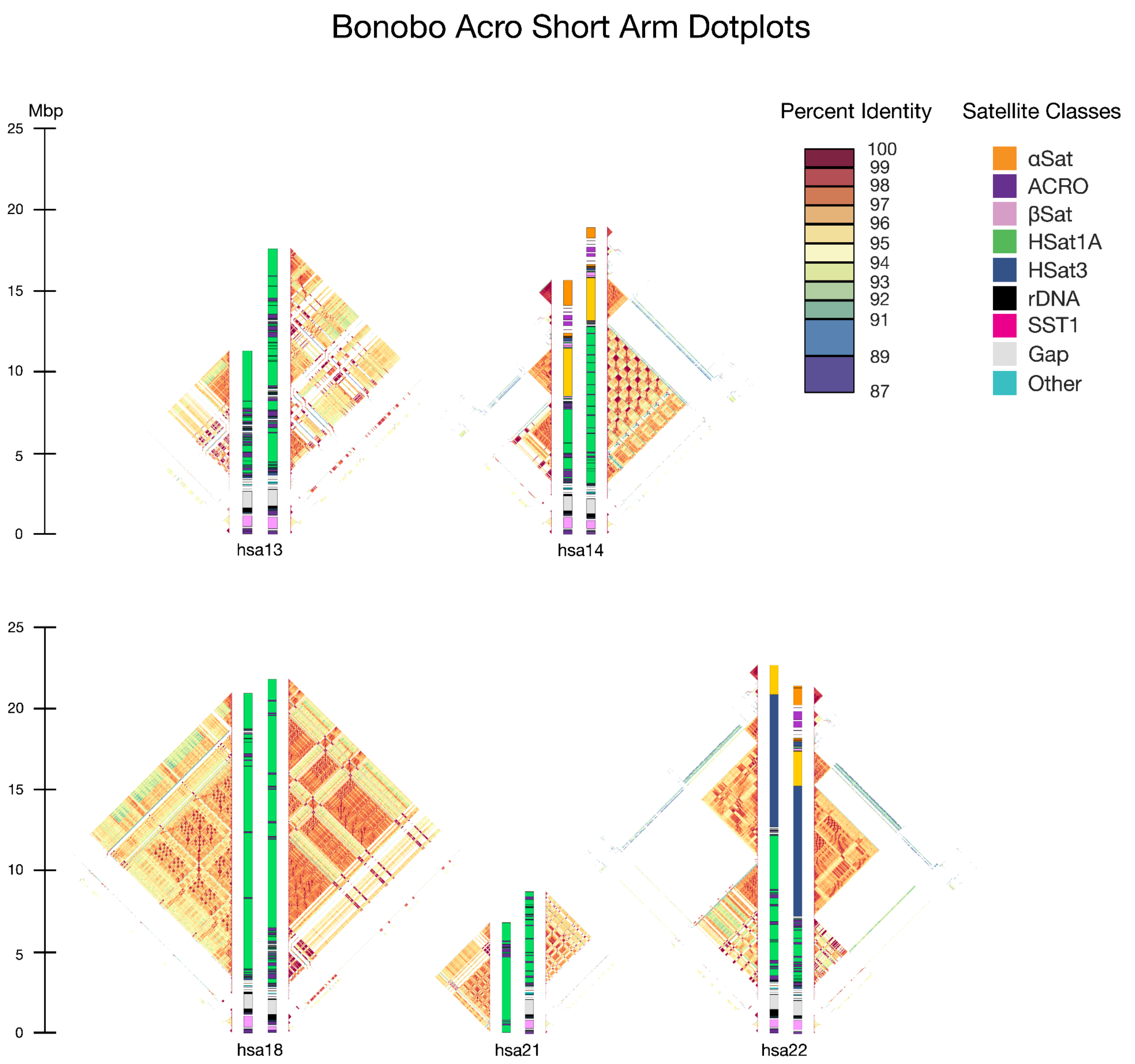

Figure S5. Self-alignment heatmaps for the short arms of the bonobo acrocentrics. ModDotPlot figures illustrate the differences in size and content between maternal (left) and paternal (right) haplotypes. Corresponding satellite annotation tracks are provided in between each plot.

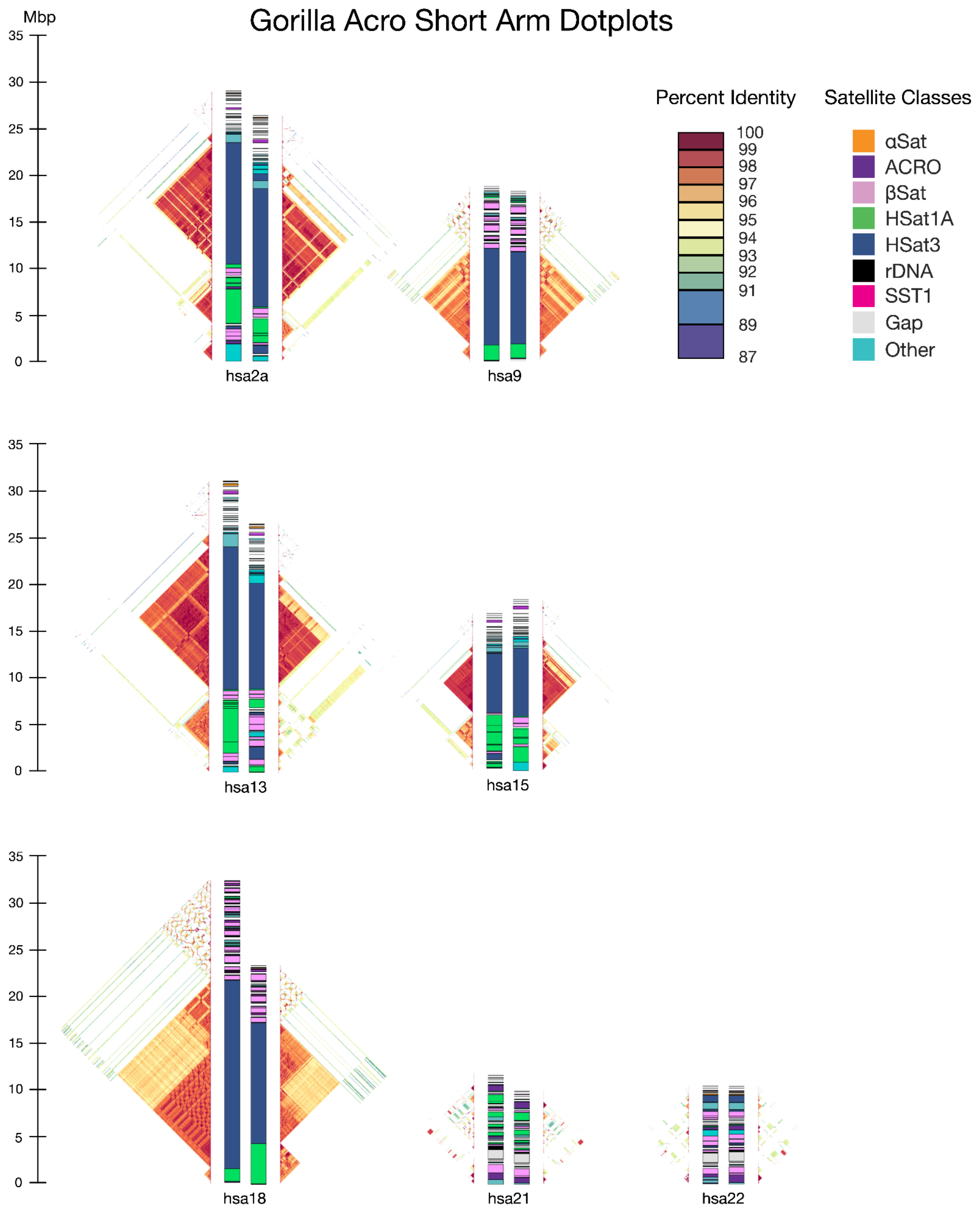

Figure S6. Self-alignment heatmaps for the short arms of the gorilla acrocentrics. ModDotPlot figures illustrate the differences in size and content between maternal (left) and paternal (right) haplotypes. Corresponding satellite annotation tracks are provided in between each plot.

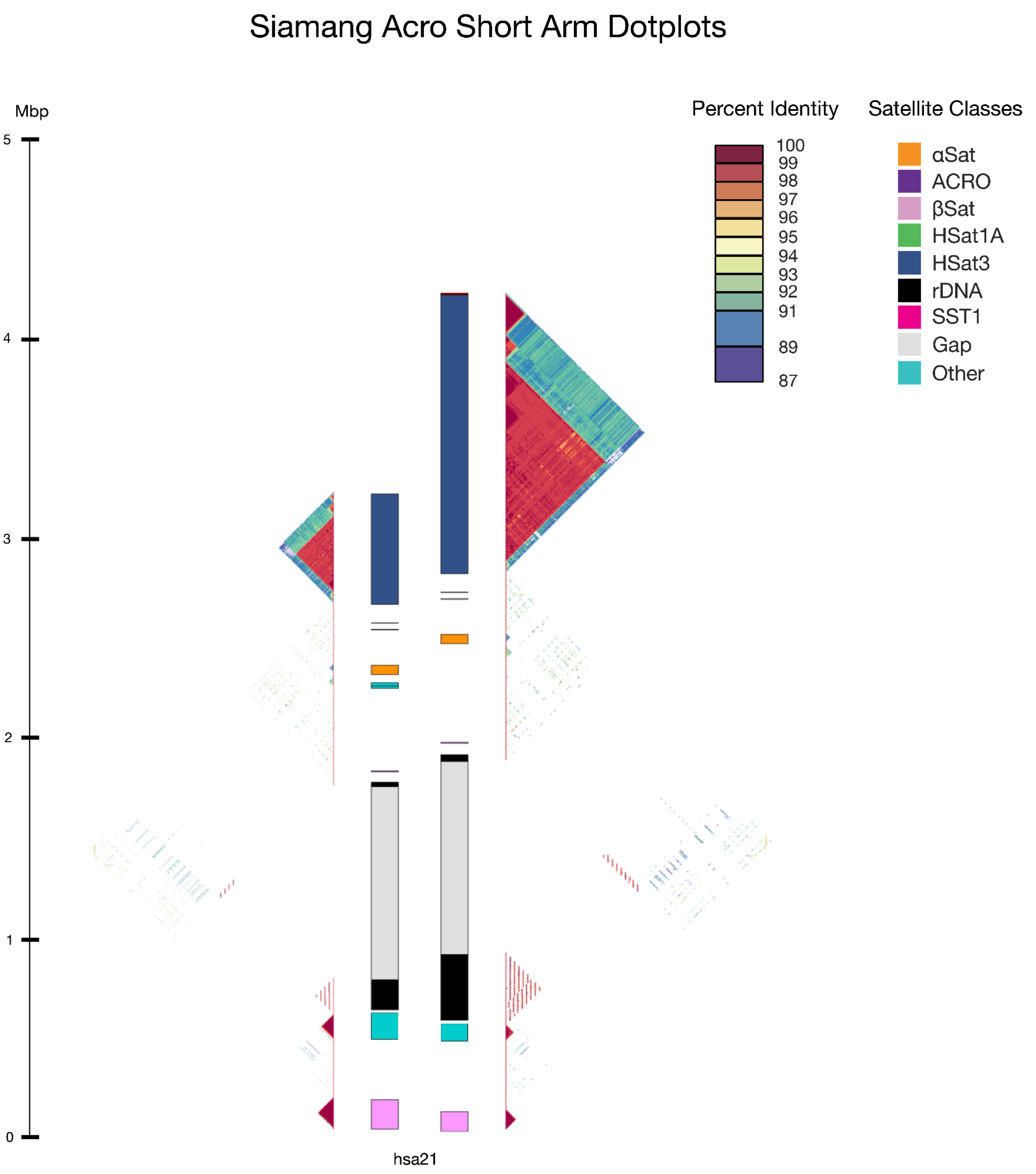

Figure S7. Self-alignment heatmaps for the short arms of the siamang acrocentrics. ModDotPlot figures illustrate the differences in size and content between primary (left) and alternate (right) haplotypes. Corresponding satellite annotation tracks are provided in between each plot.

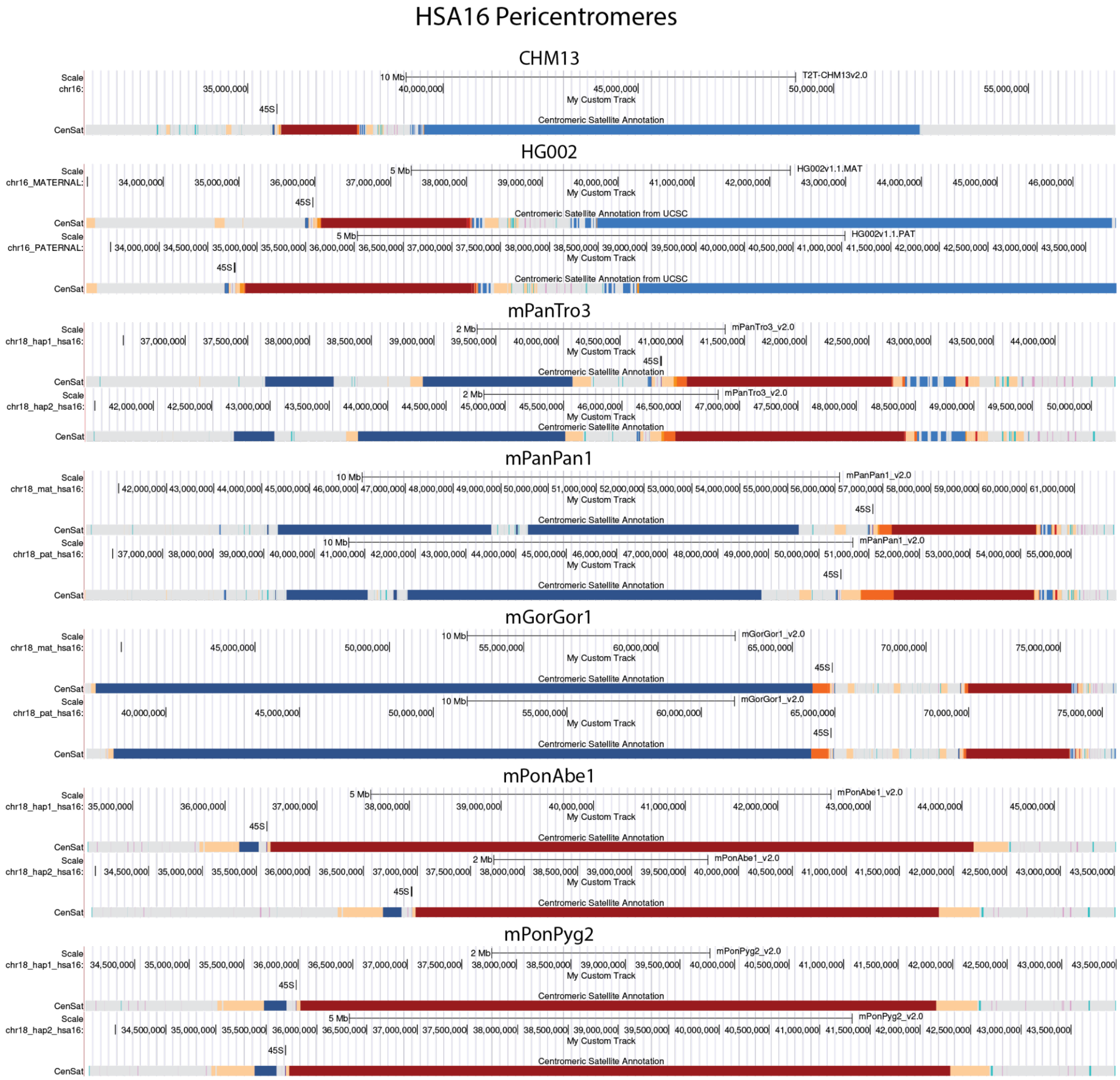

Figure S8. Location of 45S rRNA pseudogenes in the pericentromeres of HSA16. Both haplotypes of all great ape assemblies are shown. Satellite annotation tracks use the same color code as throughout the paper, with HSat2,3 as blue and the active, centromeric αSat as red. The 45S rRNA pseudogenes are labeled “45S” and marked above each satellite annotation. They are directly adjacent to the active αSat array in all species, except gorilla where they are slightly more distal and adjacent to a large HSat3 array.

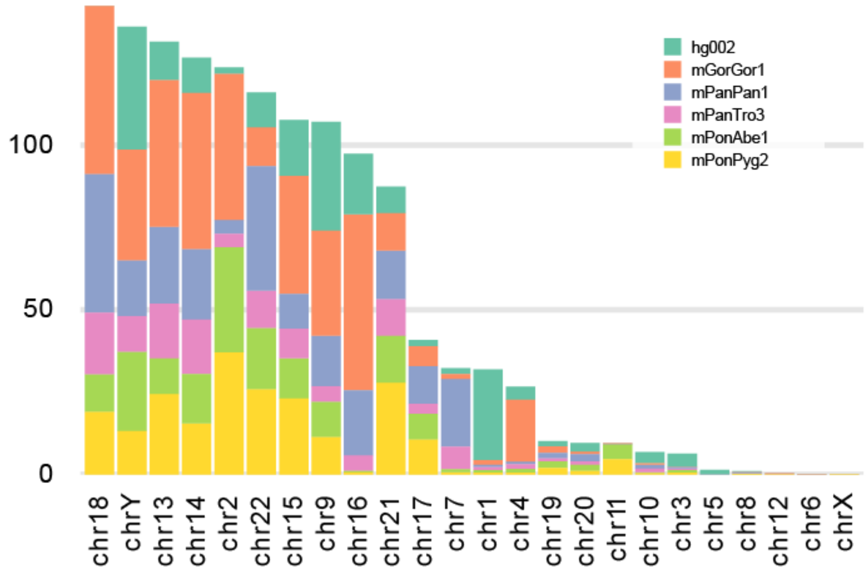

Figure S9. Acrocentric-like satellites per chromosome for each of the great apes. Total bases of acrocentric-associated satellites HSat2,3, HSat1A, βSat, ACRO, SST1, and CER, summed over each homologous human chromosome in the great apes and stacked by species. Most species have a similar amount of satellite DNA per chromosome, with notable exceptions of HSA1, HSA4, and HSA7, for which the sums are dominated by species-specific satellite expansions in human, gorilla, and chimp/bonobo, respectively.

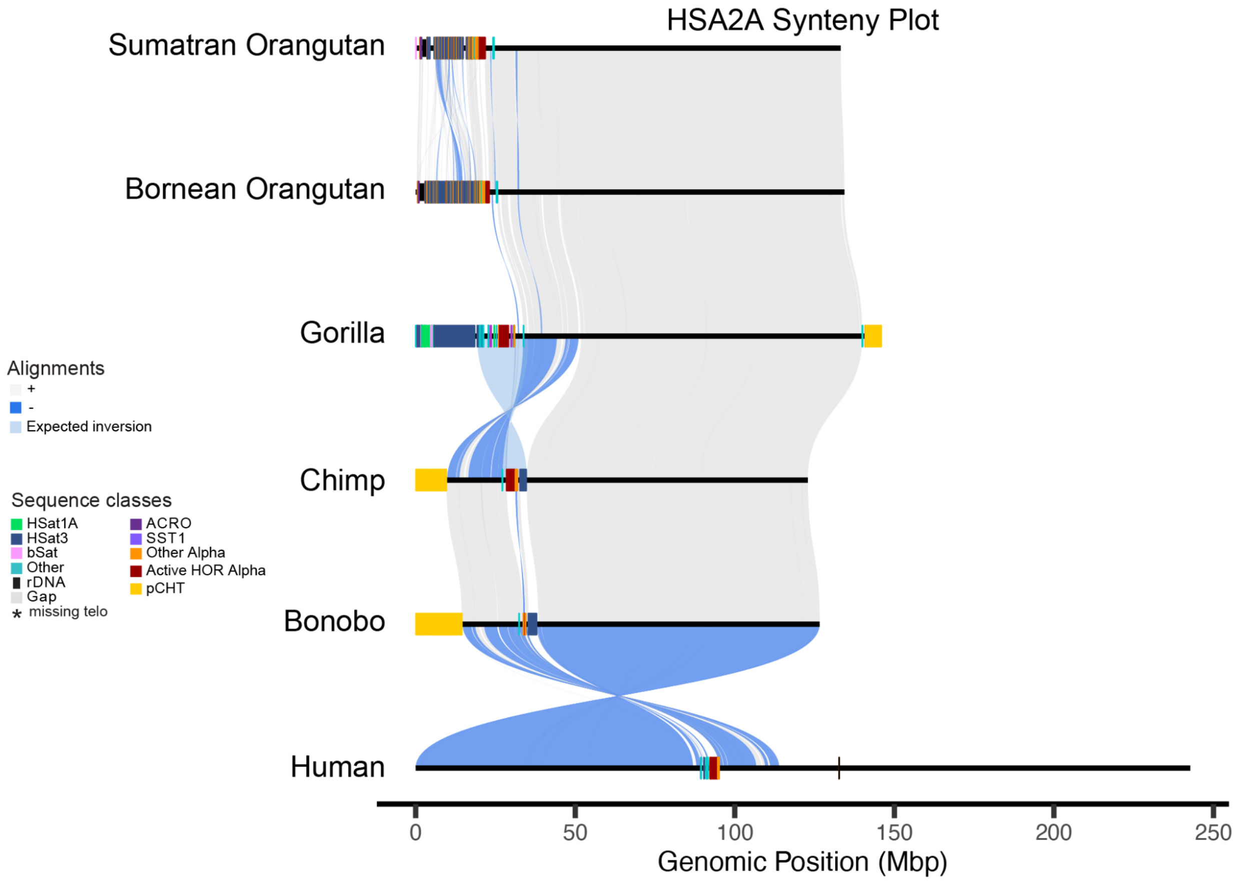

Figure S10. Whole-chromosome pairwise alignments for HSA2A. SVbyEye visualization of wfmash alignments between pairs of great ape chromosomes. Forward alignments are in grey and reverse complement in blue. Satellite annotations are given along each genome's axis with the centromeres marked in red. The whole p-arm inversion between gorilla and chimp is shown. The inversion between bonobo and human is artifactual and simply reflects the opposite orientation of the larger human fusion chromosome Chr2.

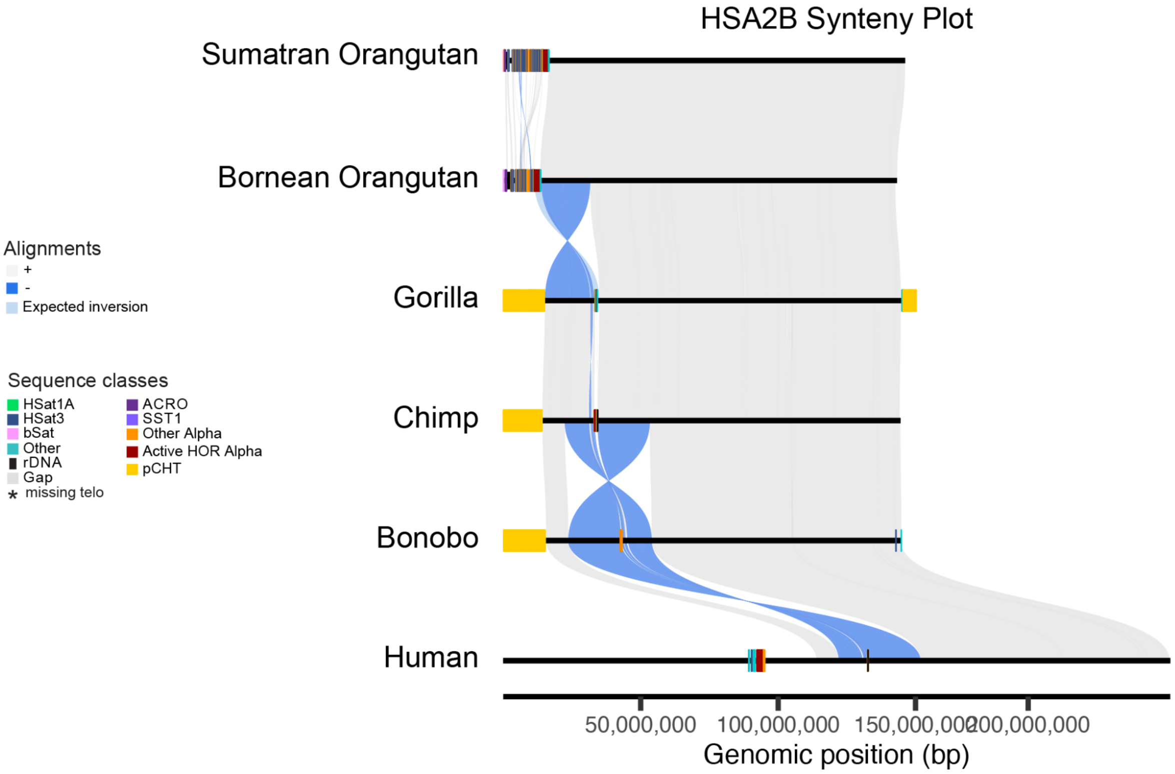

Figure S11. Whole-chromosome pairwise alignments for HSA2B. SVbyEye visualization of wfmash alignments between pairs of great ape chromosomes. Forward alignments are in grey and reverse complement in blue. Satellite annotations are given along each genome's axis with the centromeres marked in red. The whole p-arm inversion between orangutan and gorilla is shown.

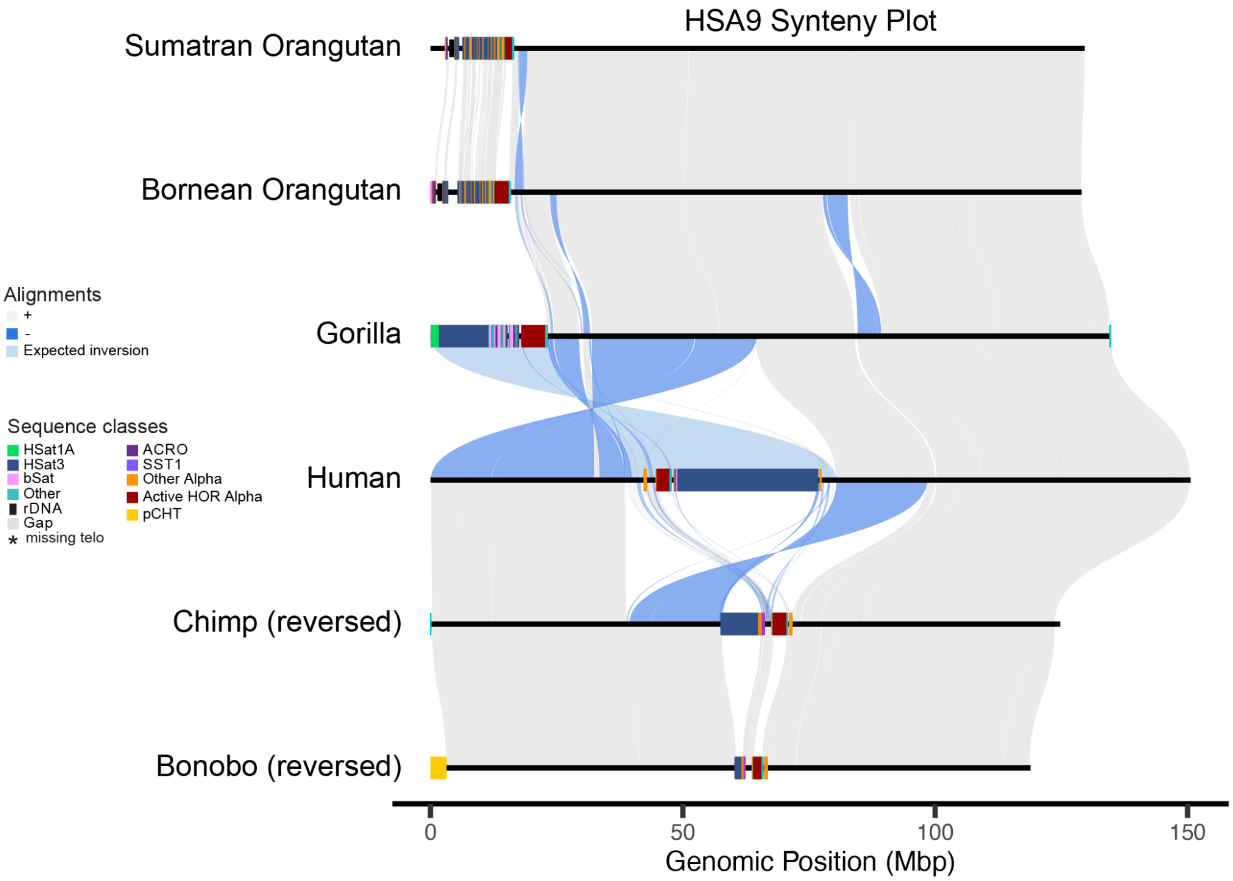

Figure S12. Whole-chromosome pairwise alignments for HSA9. SVbyEye visualization of wfmash alignments between pairs of great ape chromosomes. Forward alignments are in grey and reverse complement in blue. Satellite annotations are given along each genome's axis with the centromeres marked in red. The whole p-arm inversion between gorilla and human is shown.

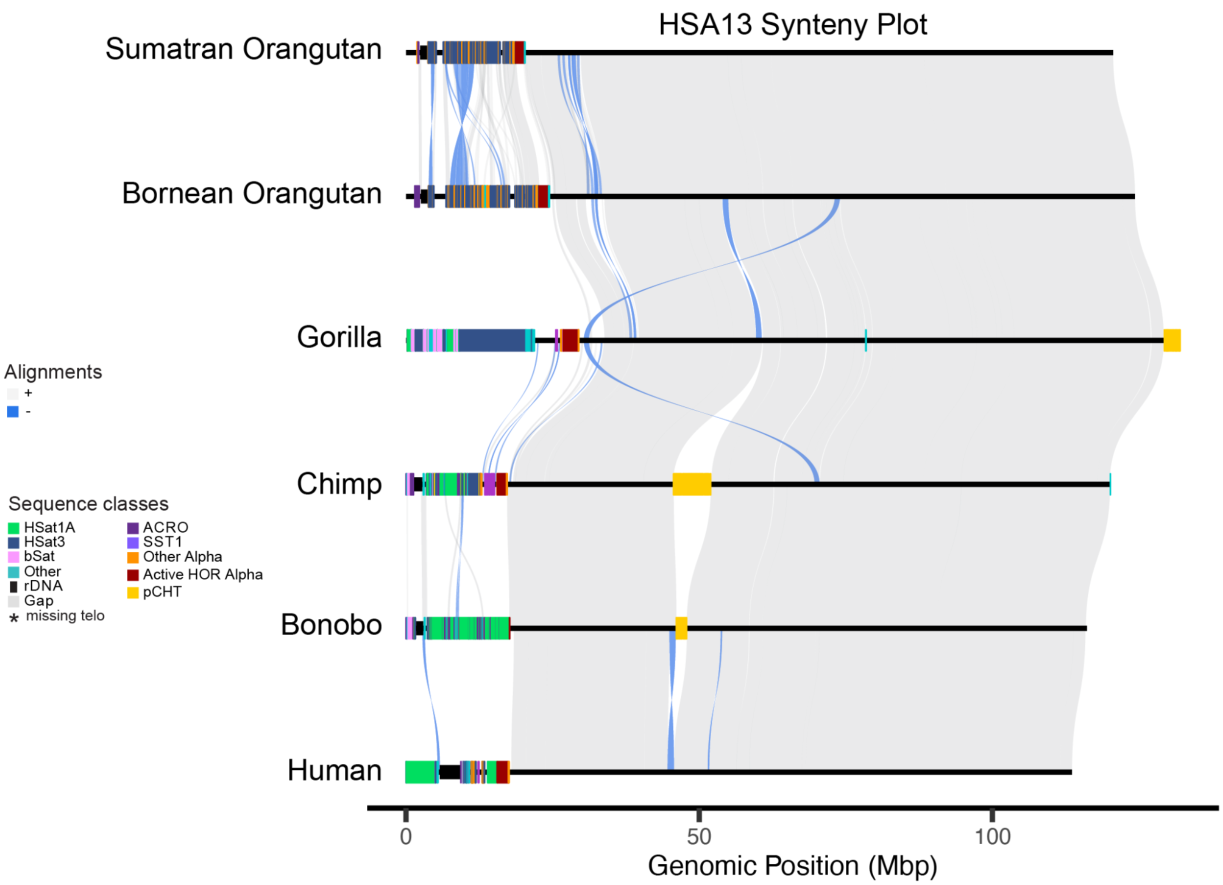

Figure S13. Whole-chromosome pairwise alignments for HSA13. SVbyEye visualization of wfmash alignments between pairs of great ape chromosomes. Forward alignments are in grey and reverse complement in blue. Satellite annotations are given along each genome's axis with the centromeres marked in red. There are no apparent whole p-arm inversions.

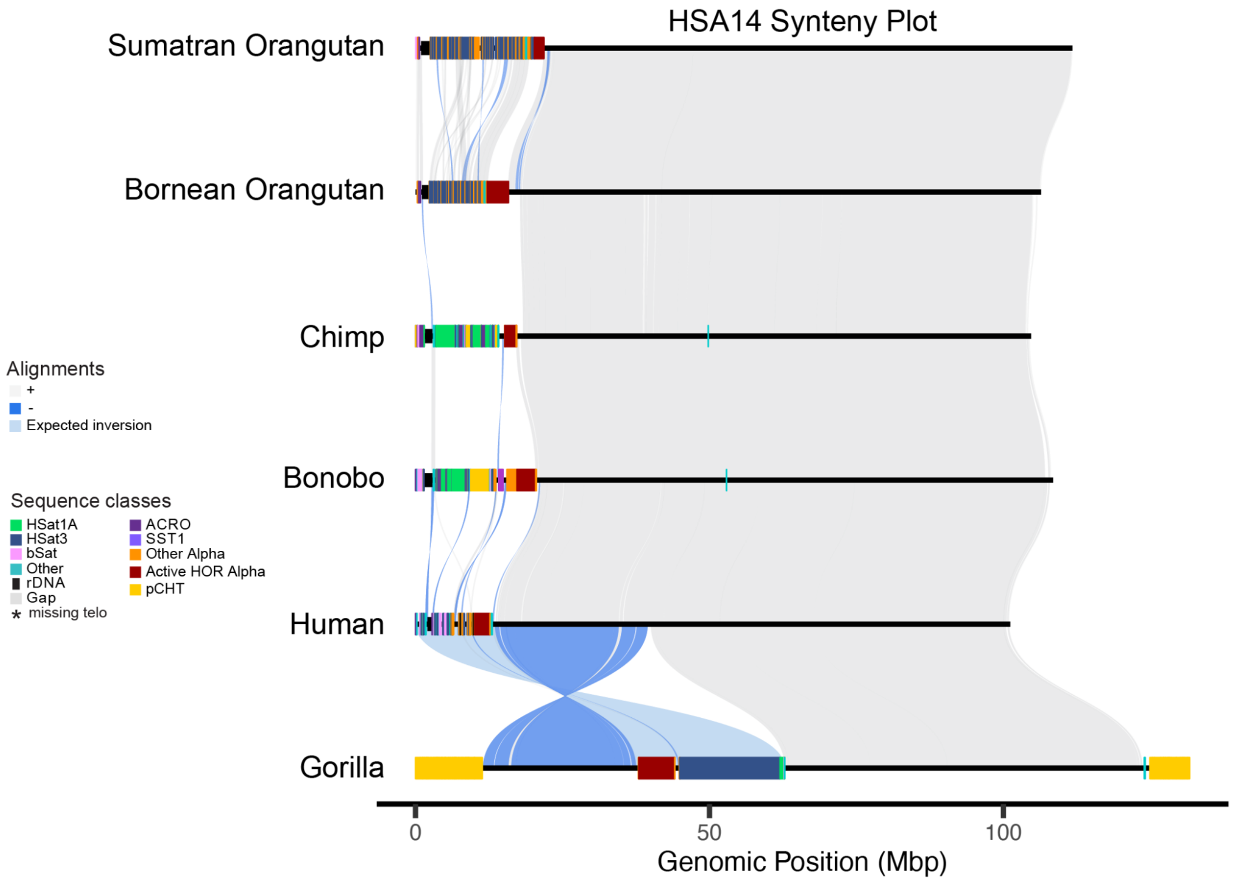

Figure S14. Whole-chromosome pairwise alignments for HSA14. SVbyEye visualization of wfmash alignments between pairs of great ape chromosomes. Forward alignments are in grey and reverse complement in blue. Satellite annotations are given along each genome's axis with the centromeres marked in red. The whole p-arm inversion between human and gorilla is shown.

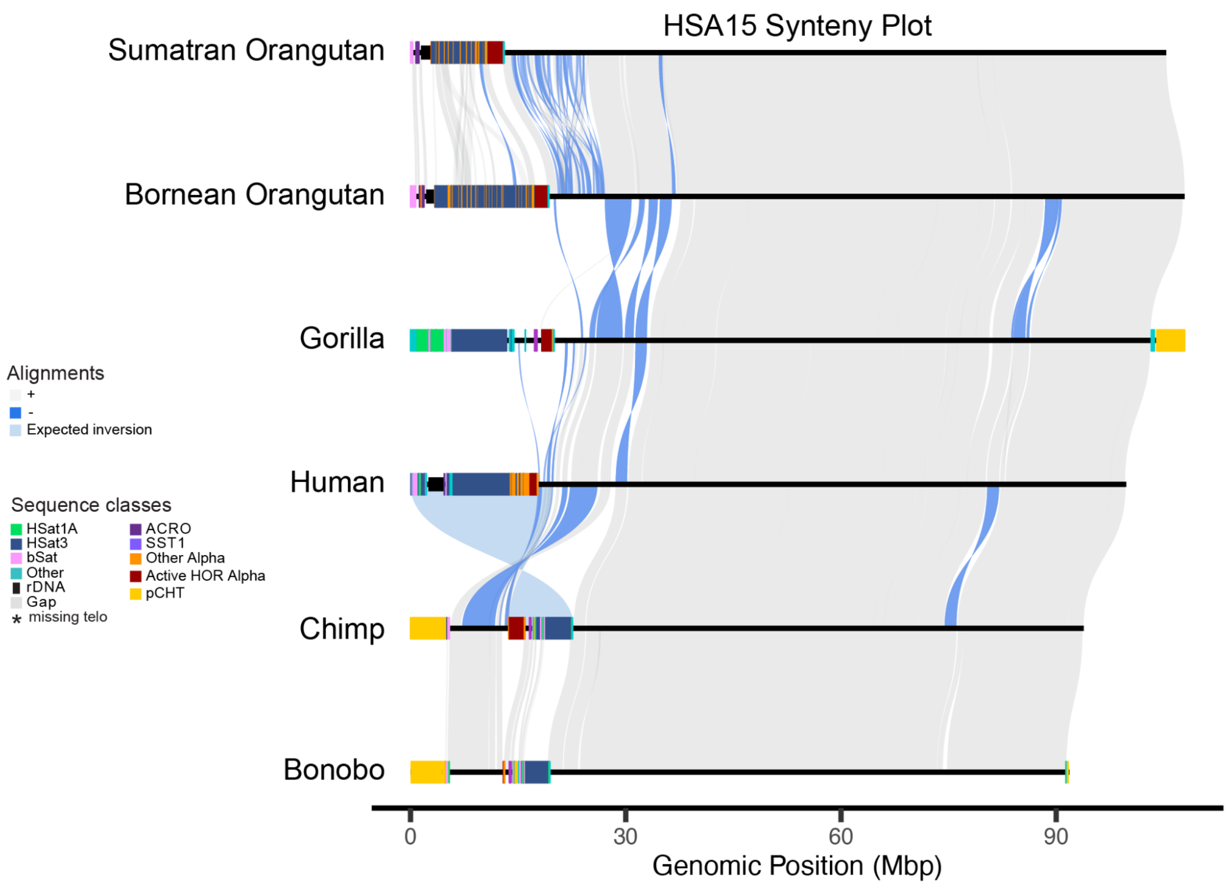

Figure S15. Whole-chromosome pairwise alignments for HSA15. SVbyEye visualization of wfmash alignments between pairs of great ape chromosomes. Forward alignments are in grey and reverse complement in blue. Satellite annotations are given along each genome's axis with the centromeres marked in red. The whole p-arm inversion between human and chimp is shown.

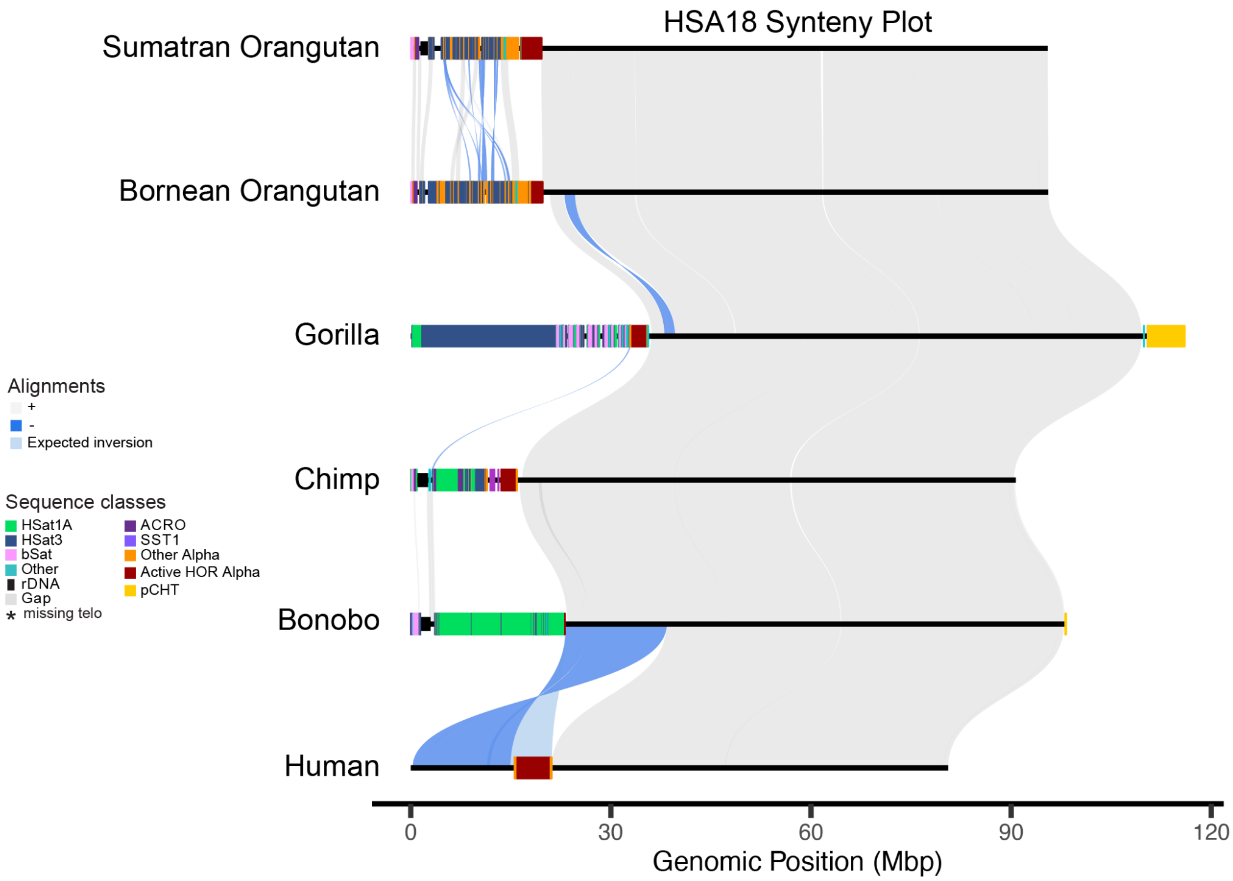

Figure S16. Whole-chromosome pairwise alignments for HSA18. SVbyEye visualization of wfmash alignments between pairs of great ape chromosomes. Forward alignments are in grey and reverse complement in blue. Satellite annotations are given along each genome's axis with the centromeres marked in red. The whole p-arm inversion between bonobo and human is shown.

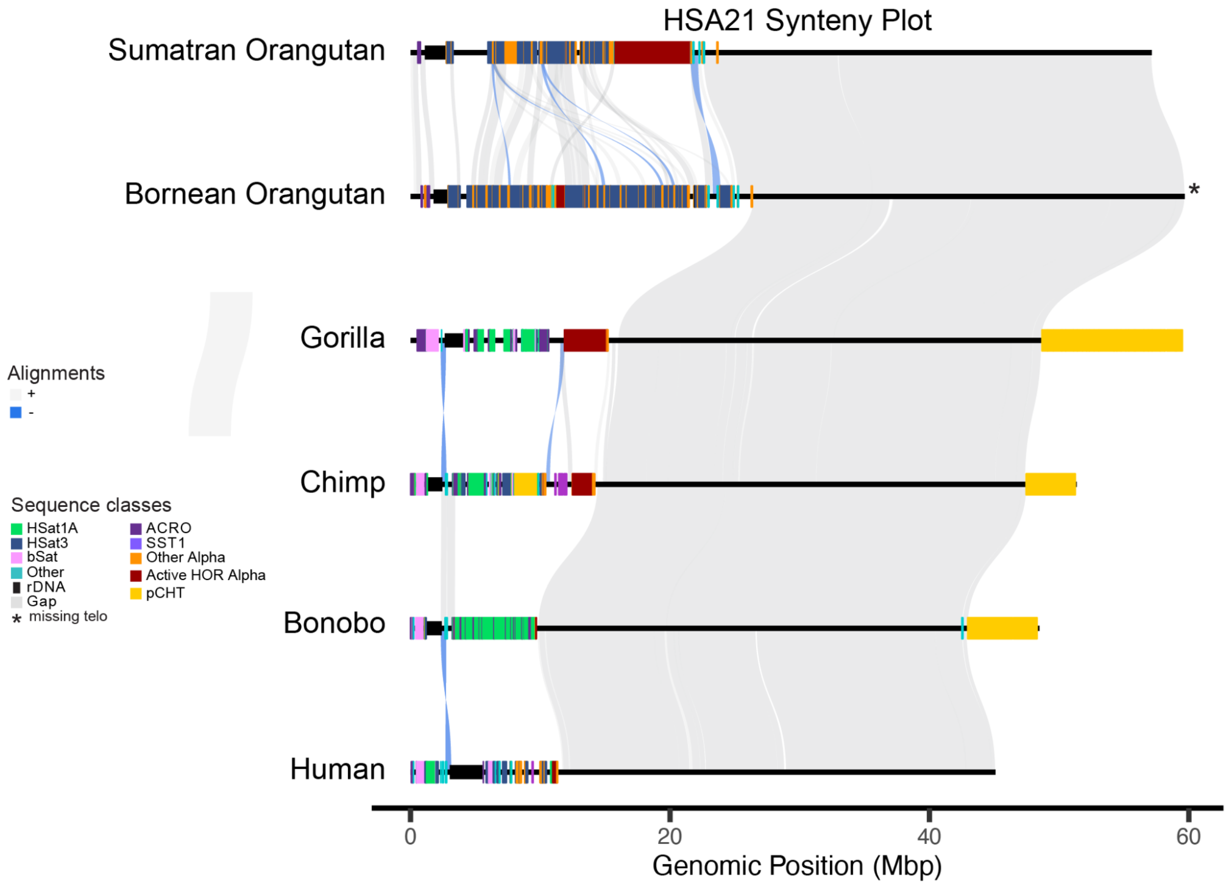

Figure S17. Whole-chromosome pairwise alignments for HSA21. SVbyEye visualization of wfmash alignments between pairs of great ape chromosomes. Forward alignments are in grey and reverse complement in blue. Satellite annotations are given along each genome's axis with the centromeres marked in red. There are no apparent whole p-arm inversions.

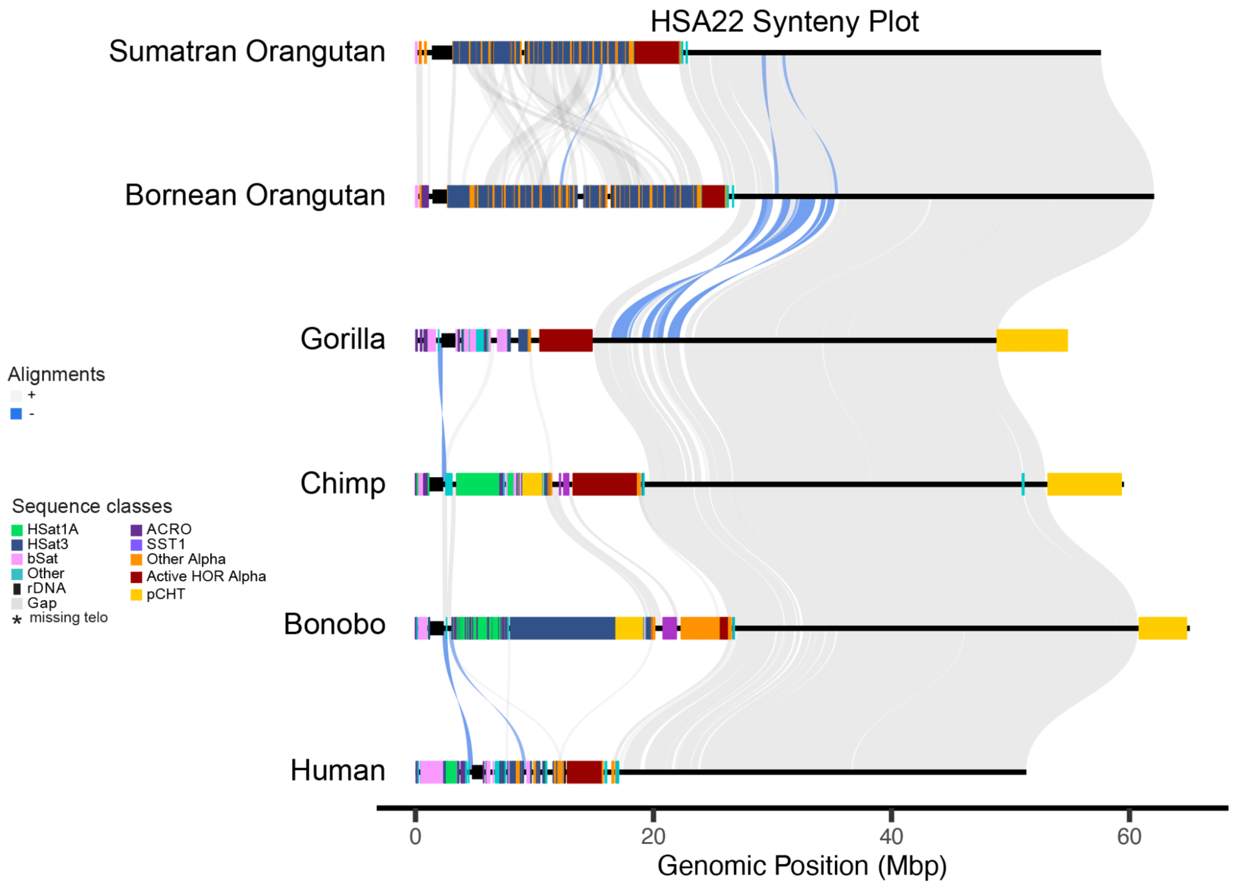

Figure S18. Whole-chromosome pairwise alignments for HSA22. SVbyEye visualization of wfmash alignments between pairs of great ape chromosomes. Forward alignments are in grey and reverse complement in blue. Satellite annotations are given along each genome's axis with the centromeres marked in red. There are no apparent whole p-arm inversions.

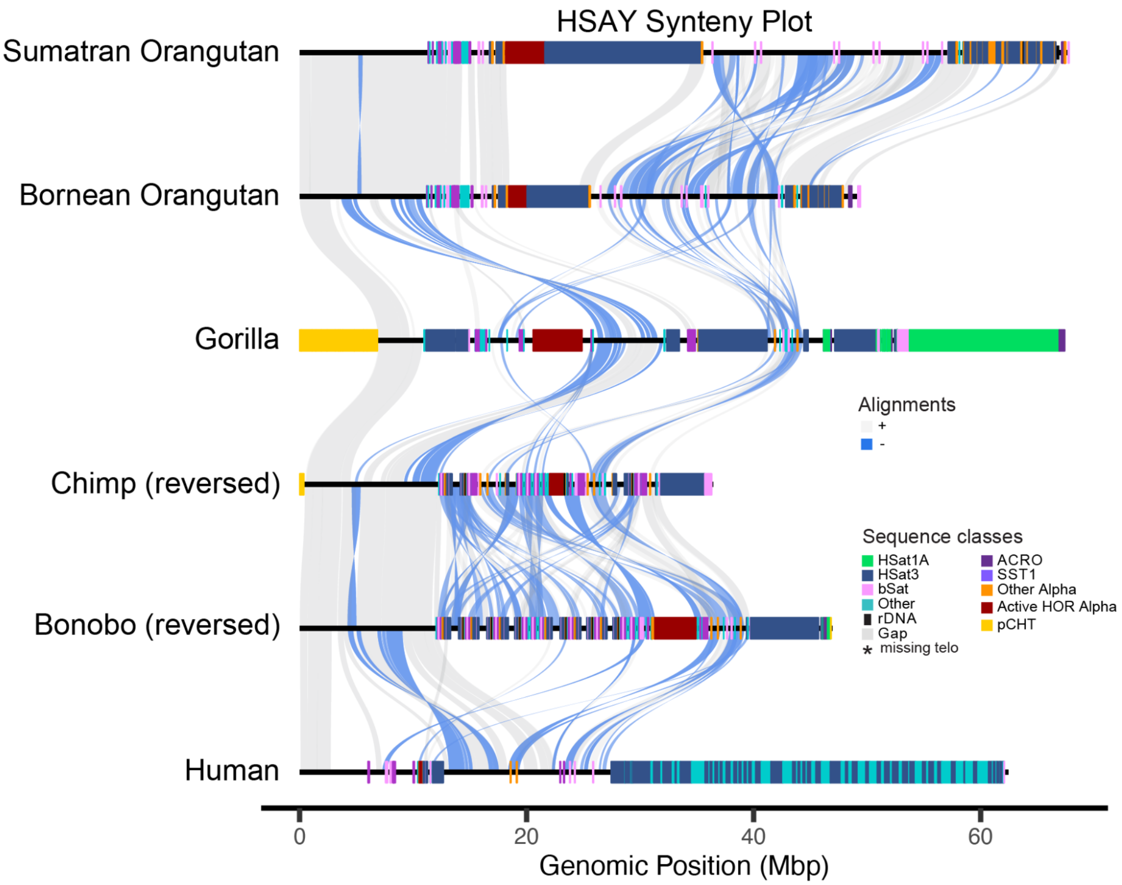

Figure S19. Whole-chromosome pairwise alignments for HSAY. SVbyEye visualization of wfmash alignments between pairs of great ape chromosomes. Forward alignments are in grey and reverse complement in blue. Satellite annotations are given along each genome's axis with the centromeres marked in red. All chromosomes are oriented with their pseudoautosomal region (PAR1) on the left, regardless of centromere position.

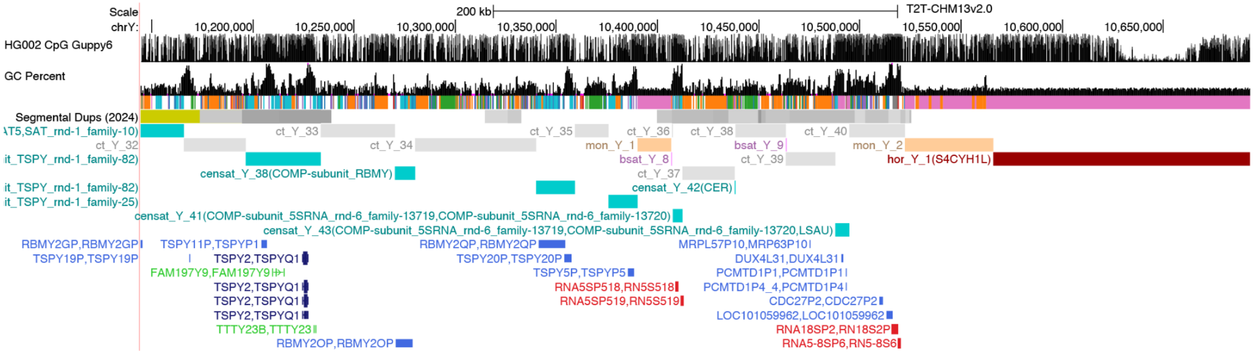

Figure S20. Location of 45S rDNA fragments in the pericentromere of human HG002 ChrY. UCSC Genome Browser tracks from top to bottom show: %methylation, %GC, RepeatMasker annotations, segmental duplications, satellite annotations, and gene annotations. The centromeric, active αSat array is marked in dark red (hor_Y_1). Annotated rDNAs are below in red, including both 5S (left) and 45S/18S/5.8S (right).

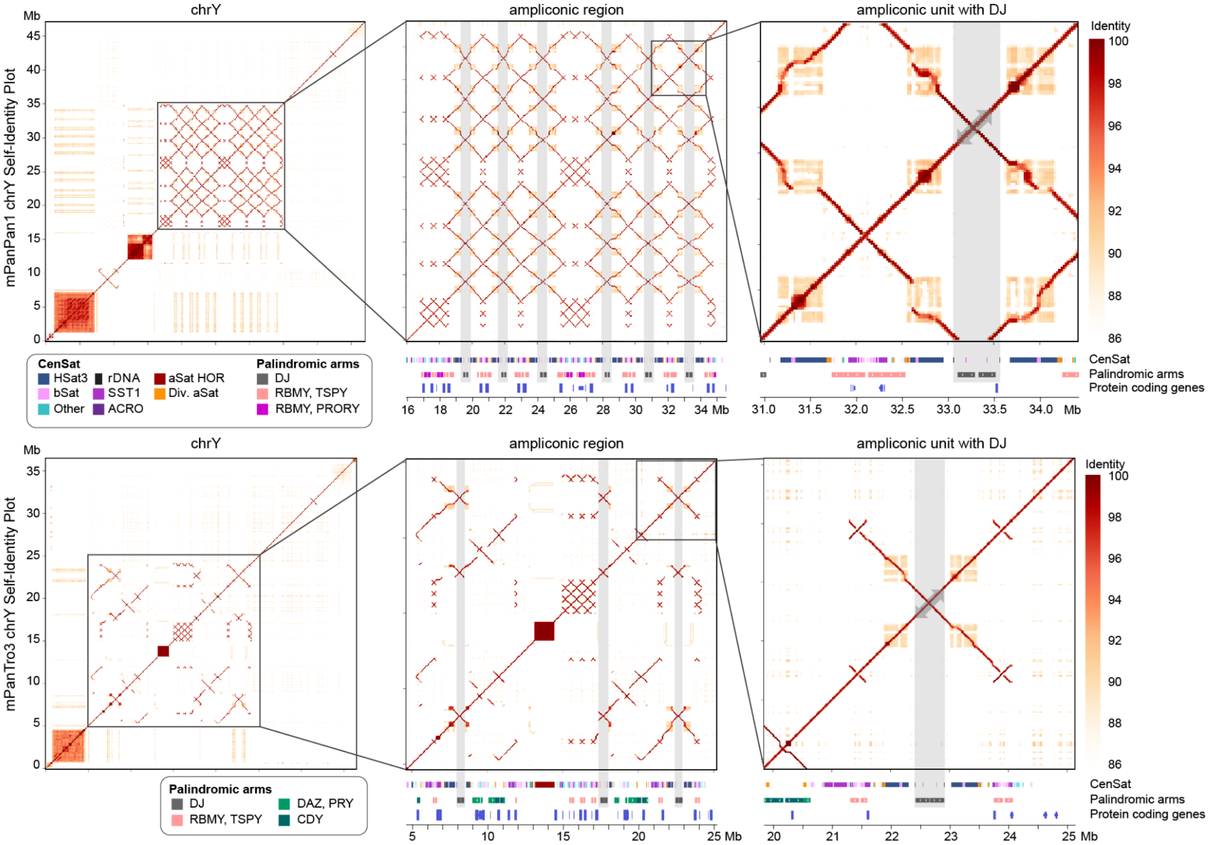

Figure S21. Amplification of a 45S rDNA DJ-like sequence alongside *TSPY* and *RBMY* on ChrY of chimp and bonobo. Chimp and bonobo Y chromosomes contain amplified, palindromic arrays of *TSPY* and *RBMY* genes. Spacing between these amplified gene copies is the palindromic repeat unit characteristic of the 45S rDNA distal junction sequence (highlighted in gray). These amplified regions are progressively zoomed from left to right to show the fine-grained structure.

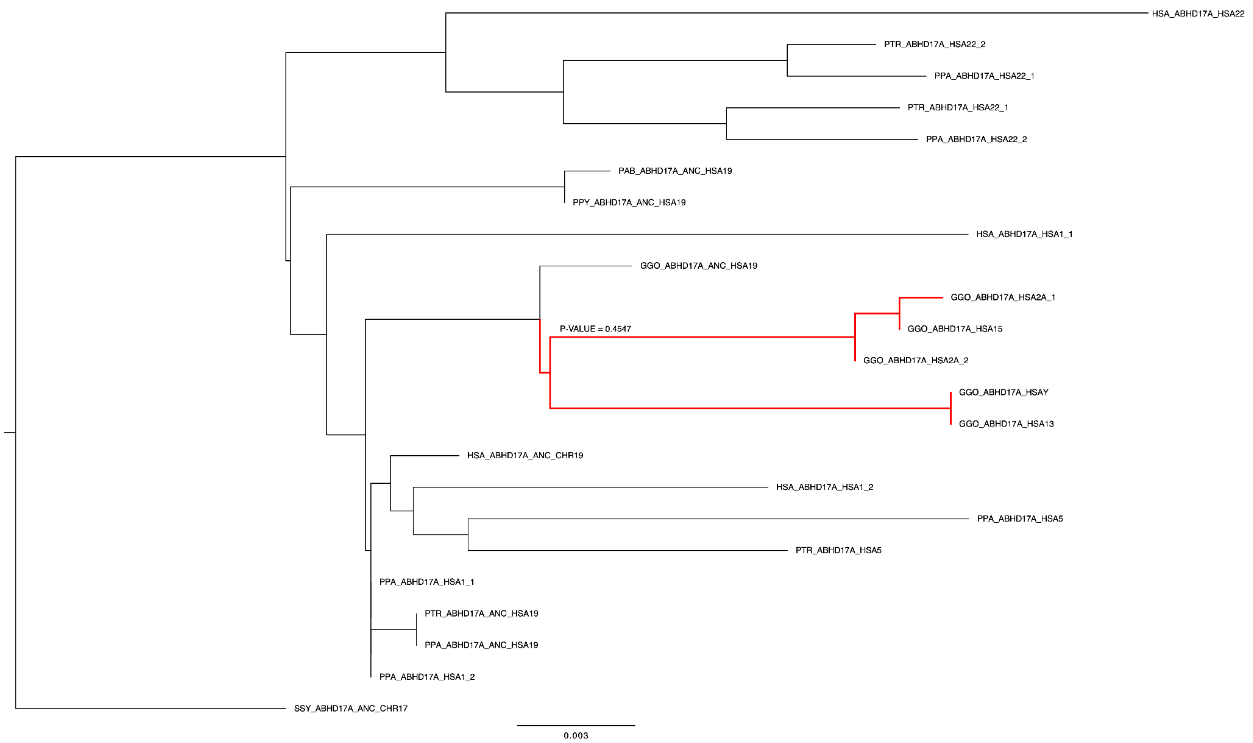

Figure S22. CDS tree for *ABHD17A*. ML tree built from gap-trimmed alignment. BUSTED positive selection testing was run on the red-highlighted branches as the foreground.

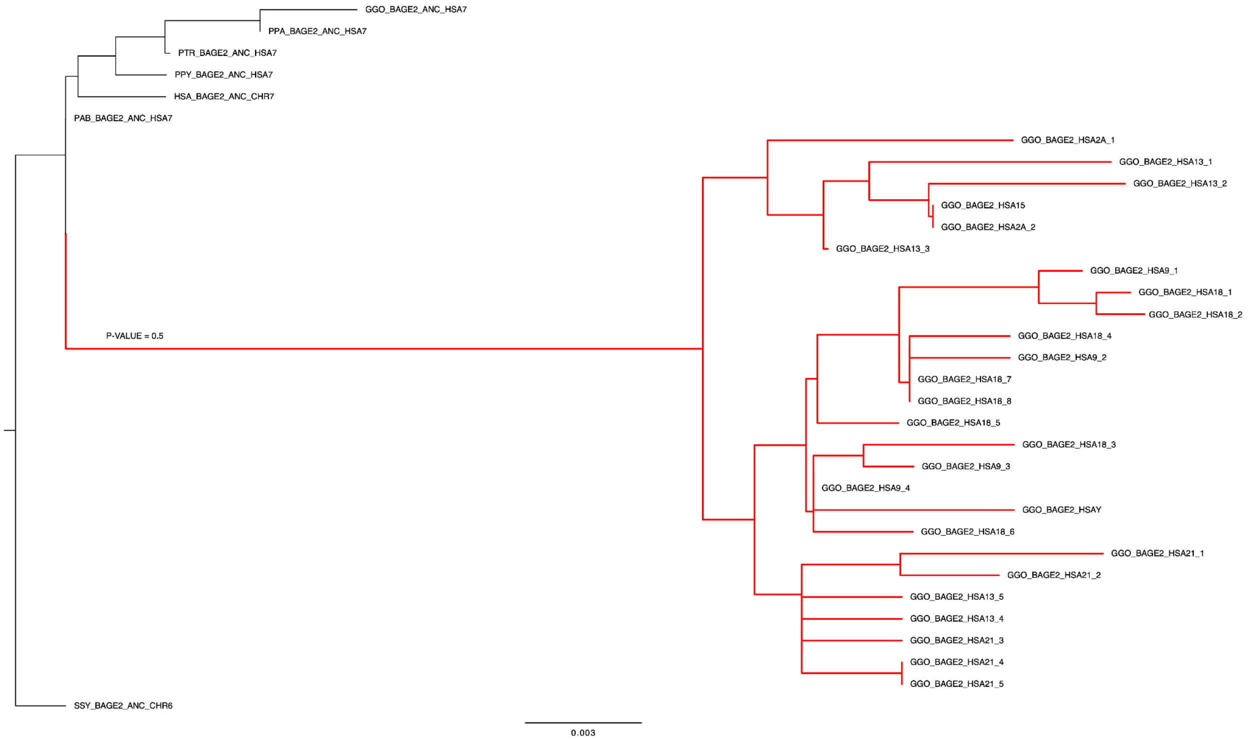

Figure S23. CDS tree for *BAGE2*. ML tree built from gap-trimmed alignment. BUSTED positive selection testing was run on the red-highlighted branches as the foreground.

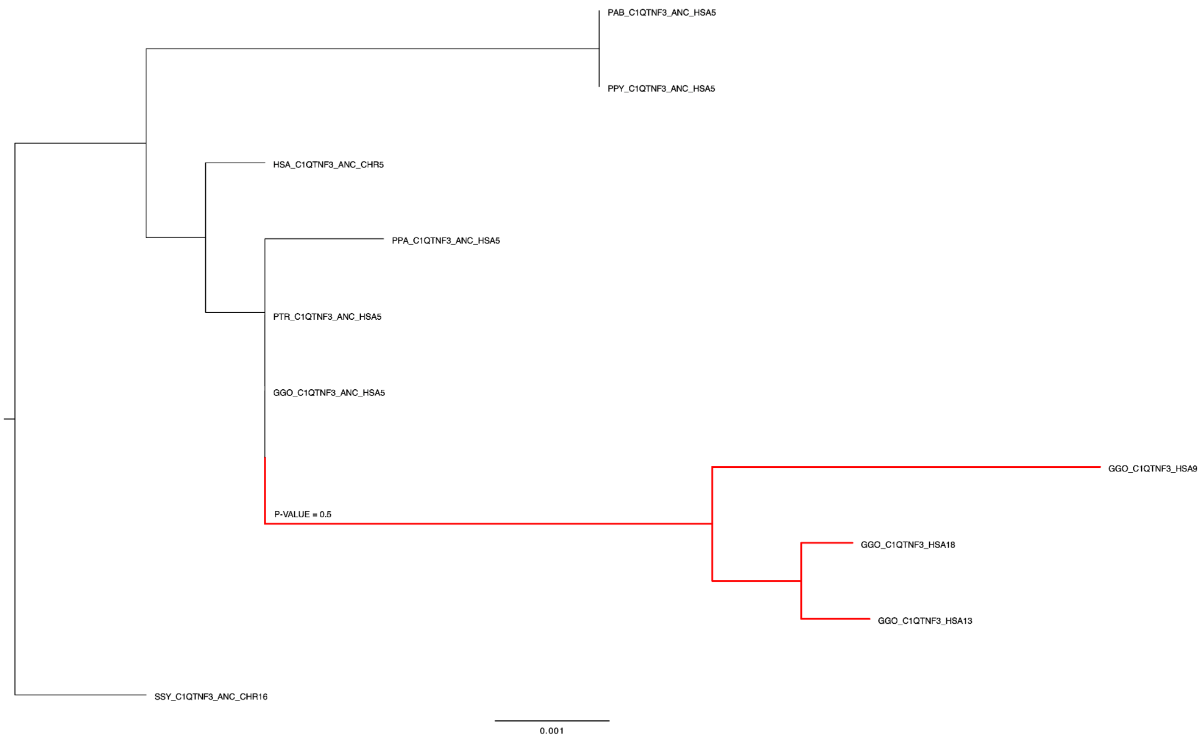

Figure S24. CDS tree for *C1QTNF3*. ML tree built from gap-trimmed alignment. BUSTED positive selection testing was run on the red-highlighted branches as the foreground.

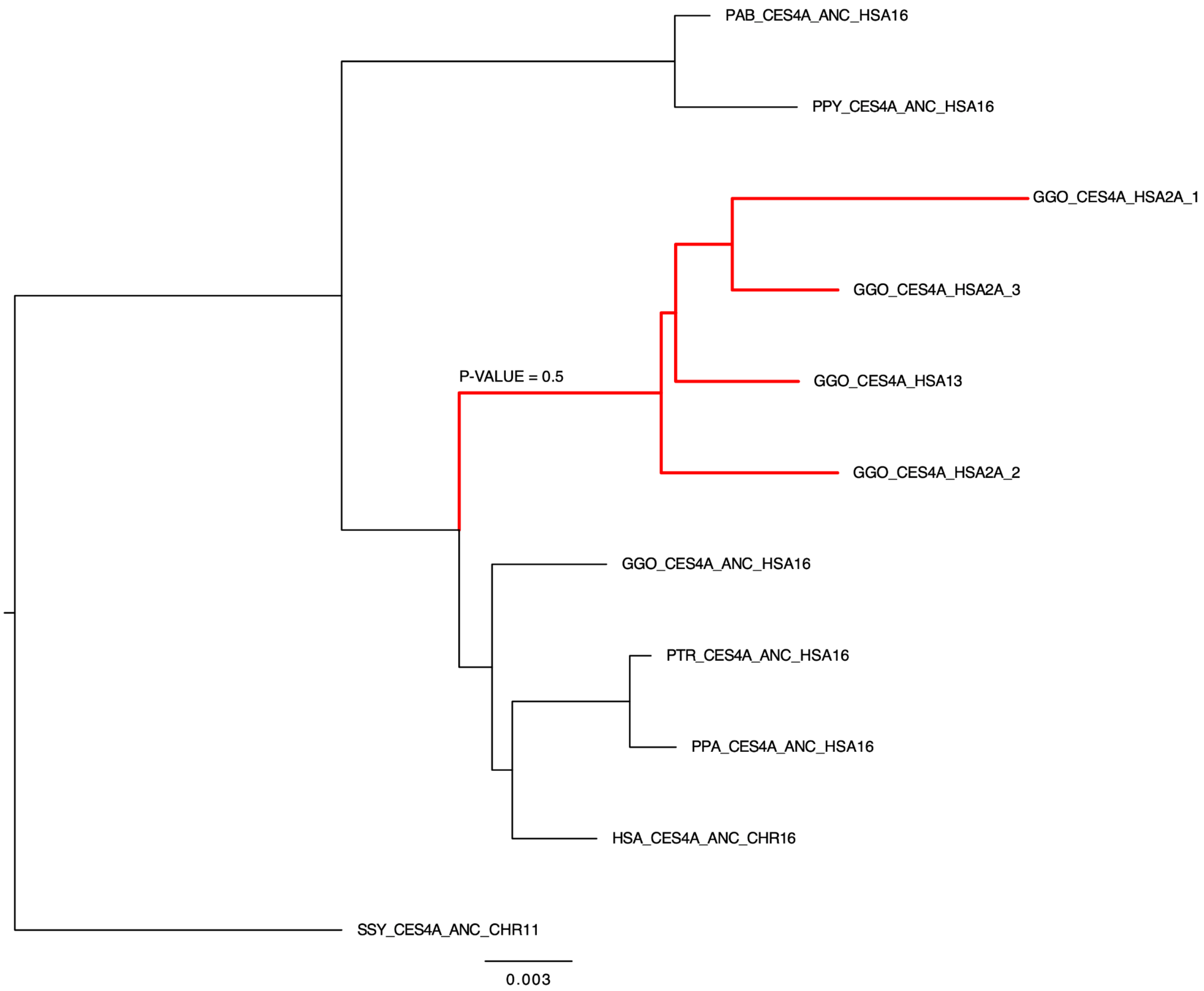

Figure S25. CDS tree for *CES4A*. ML tree built from gap-trimmed alignment. BUSTED positive selection testing was run on the red-highlighted branches as the foreground.

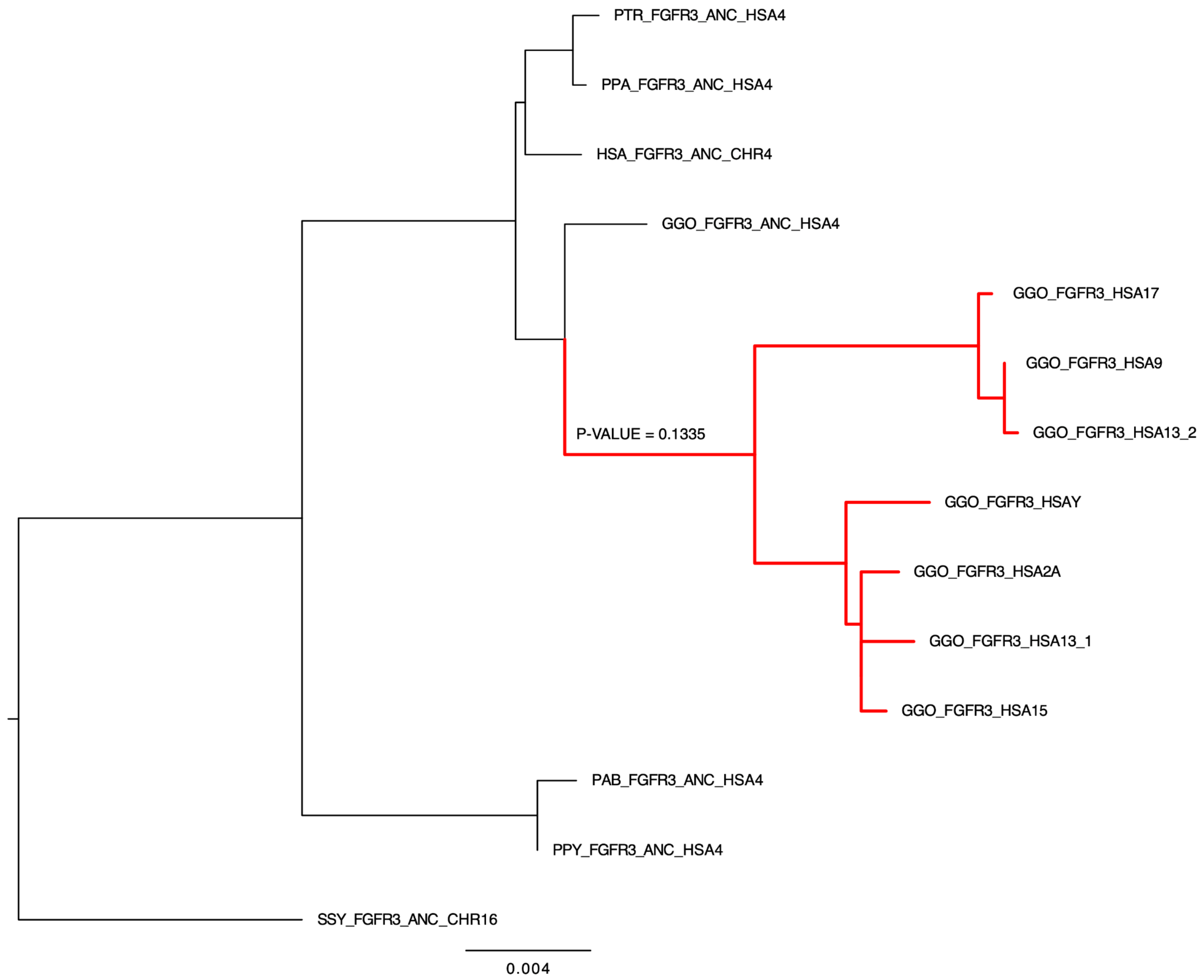

Figure S26. CDS tree for *FGFR3*. ML tree built from gap-trimmed alignment. BUSTED positive selection testing was run on the red-highlighted branches as the foreground.

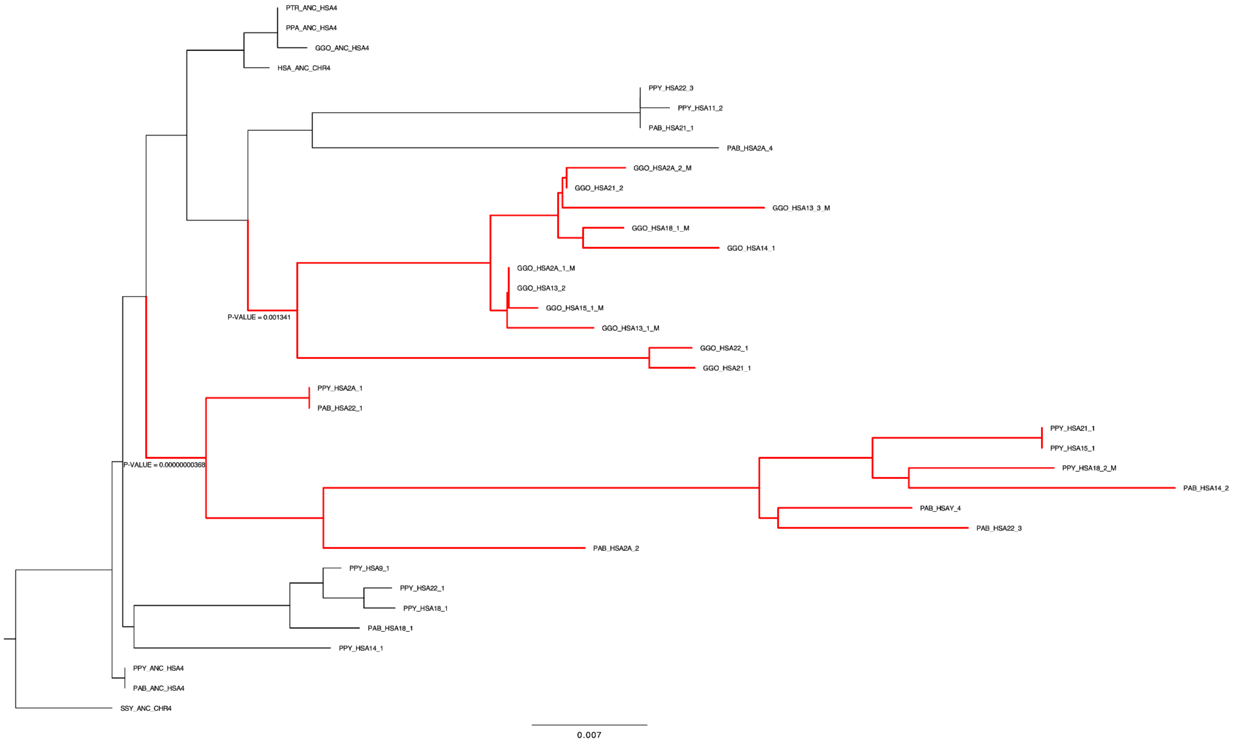

Figure S27. CDS tree for *FRG1*. ML tree built from gap-trimmed alignment. BUSTED positive selection testing was run on the red-highlighted branches as the foreground, separately for the orangutan and gorilla clades.

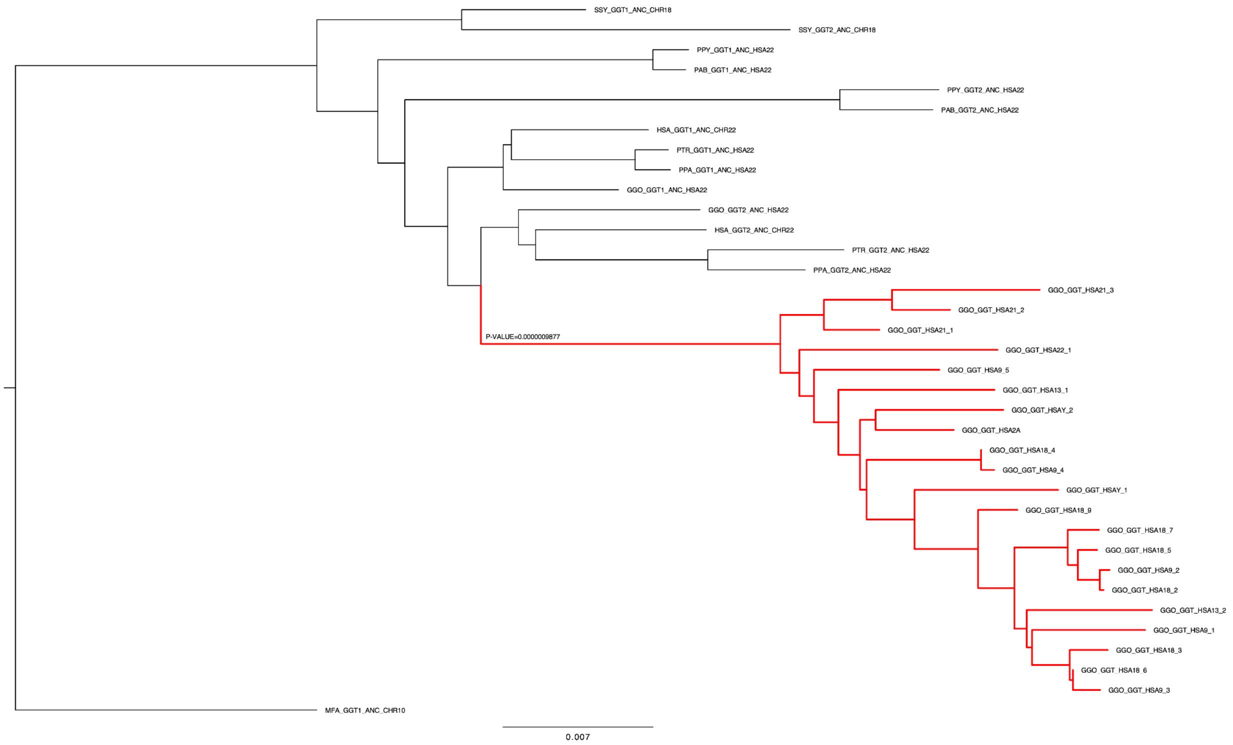

Figure S28. CDS tree for *GGT*. ML tree built from gap-trimmed alignment. BUSTED positive selection testing was run on the red-highlighted branches as the foreground.

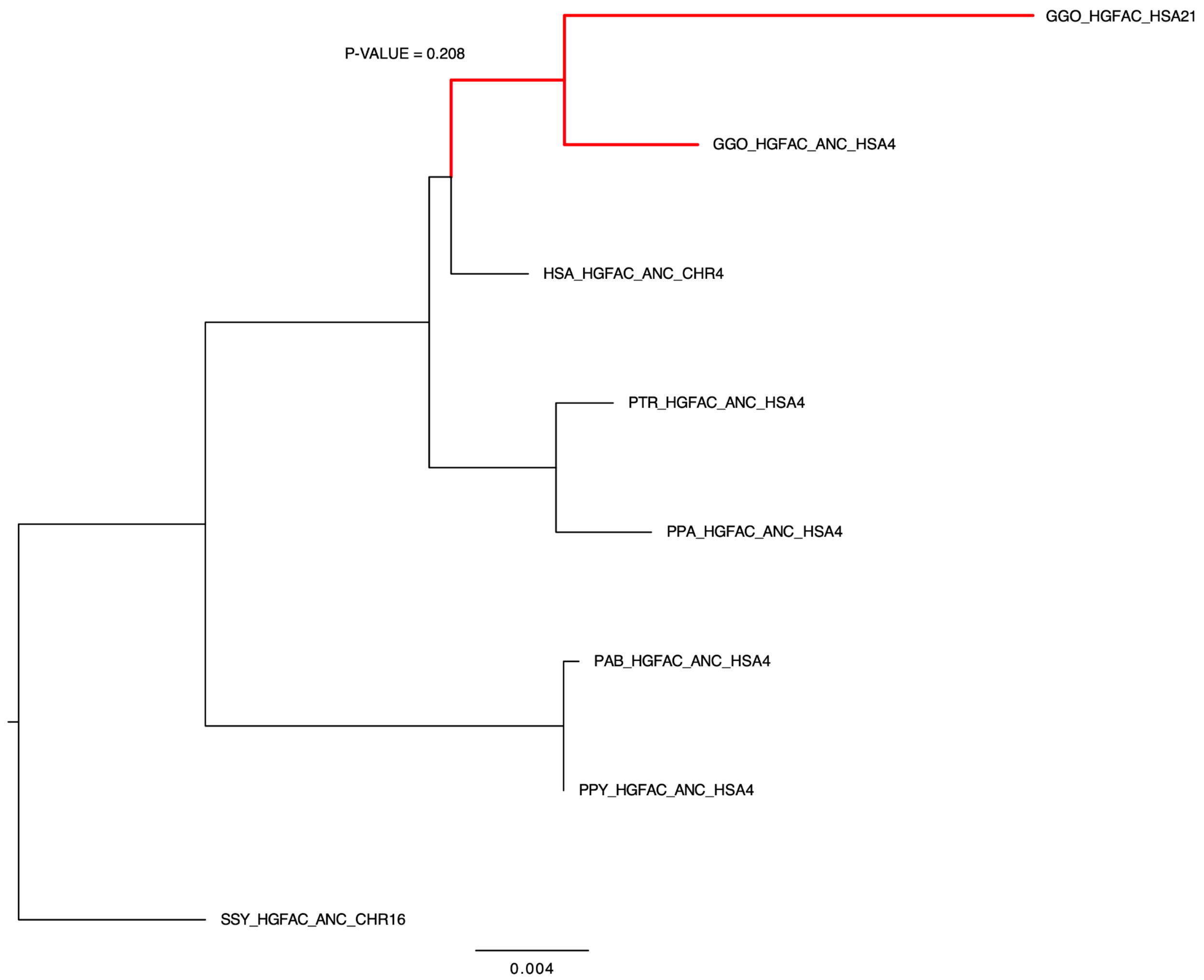

Figure S29. CDS tree for *HGFAC*. ML tree built from gap-trimmed alignment. BUSTED positive selection testing was run on the red-highlighted branches as the foreground.

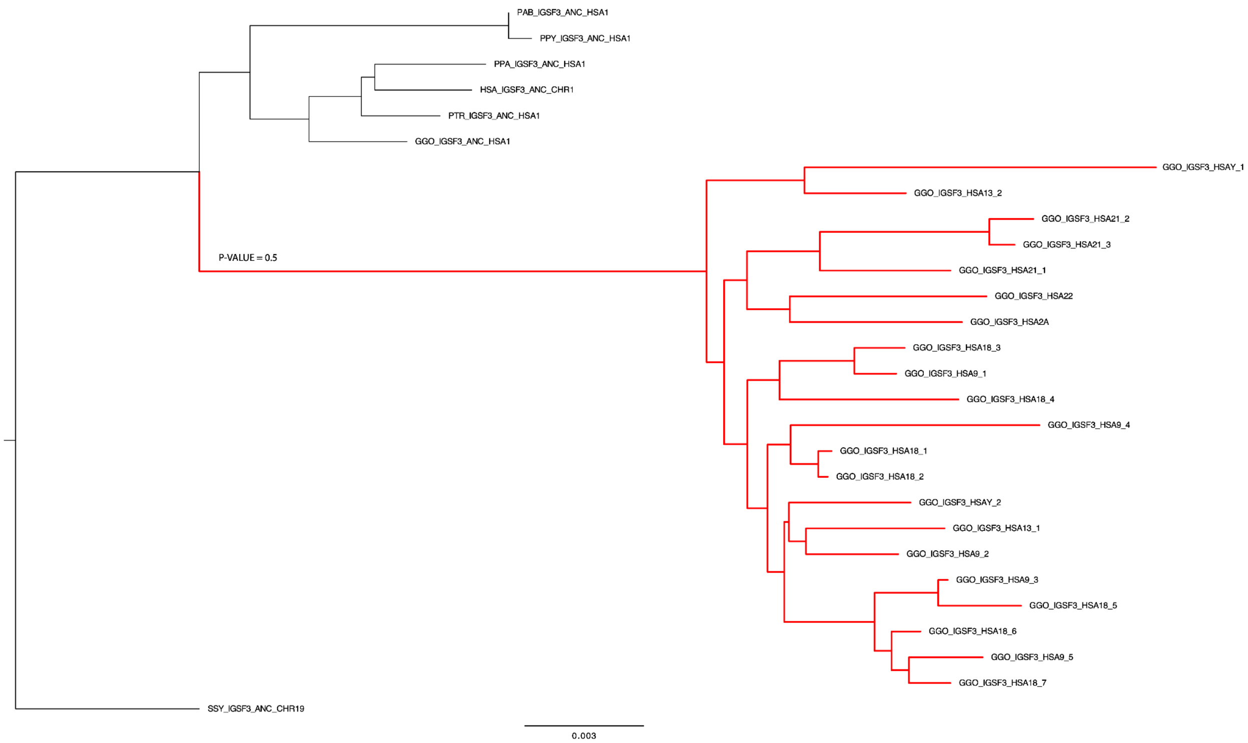

Figure S30. CDS tree for *IGSF3*. ML tree built from gap-trimmed alignment. BUSTED positive selection testing was run on the red-highlighted branches as the foreground.

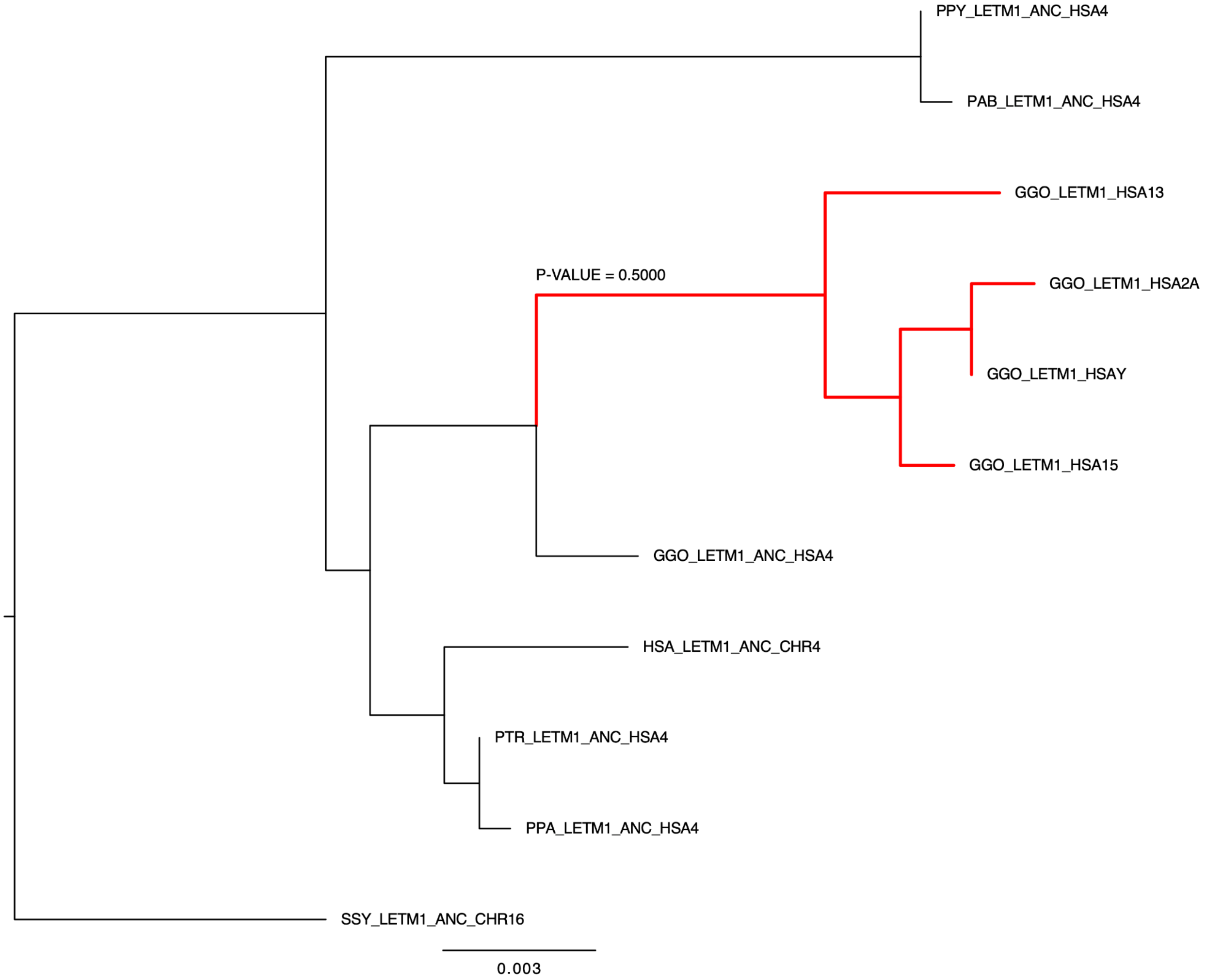

Figure S31. CDS tree for *LETM1*. ML tree built from gap-trimmed alignment. BUSTED positive selection testing was run on the red-highlighted branches as the foreground.

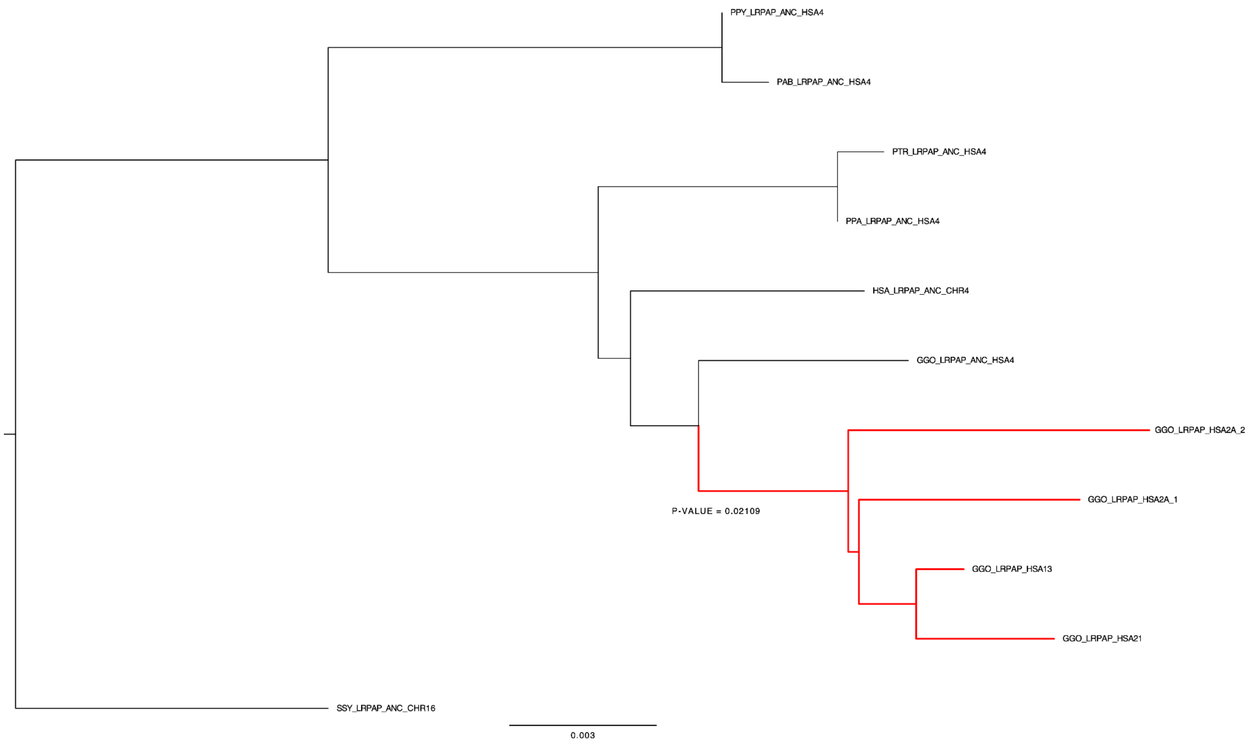

Figure S32. CDS tree for *LRPAP1*. ML tree built from gap-trimmed alignment. BUSTED positive selection testing was run on the red-highlighted branches as the foreground.

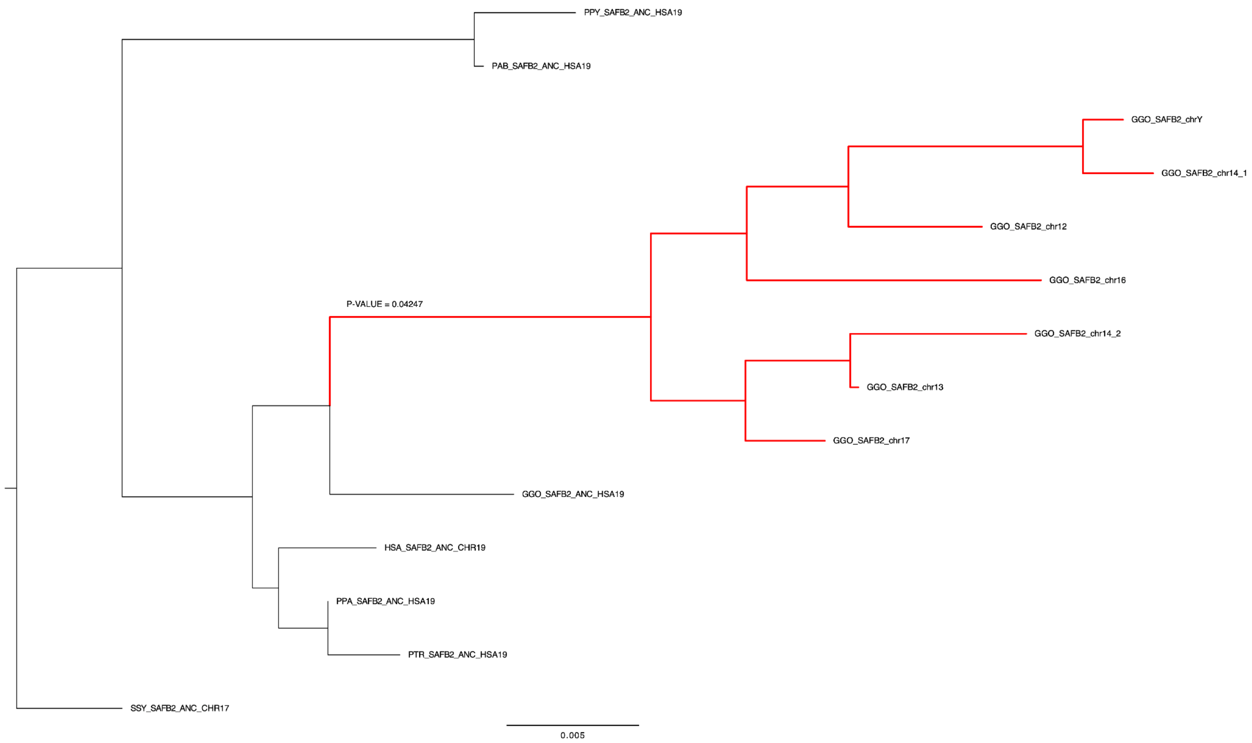

Figure S33. CDS tree for *SAFB2*. ML tree built from gap-trimmed alignment. BUSTED positive selection testing was run on the red-highlighted branches as the foreground.

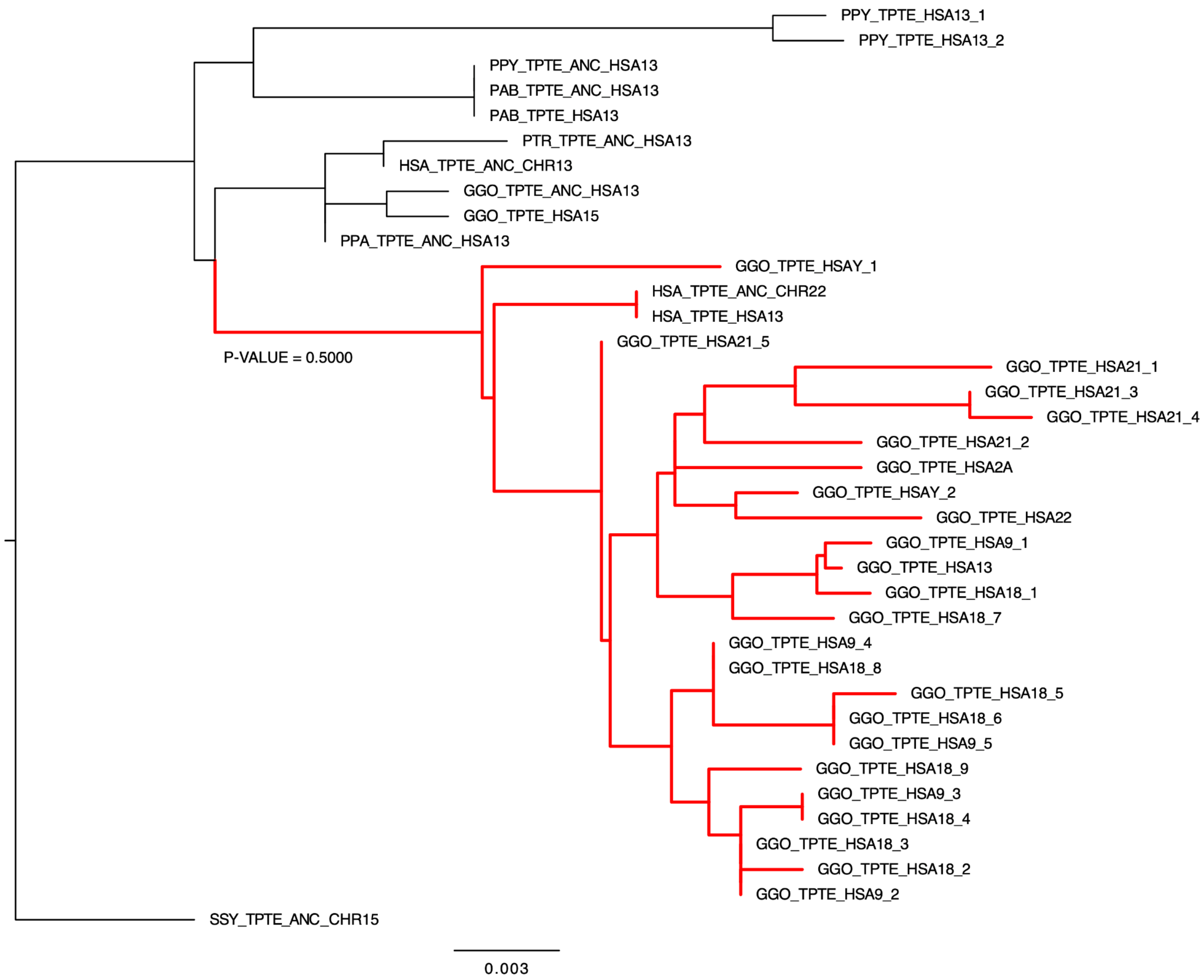

Figure S34. CDS tree for *TPTE*. ML tree built from gap-trimmed alignment. BUSTED positive selection testing was run on the red-highlighted branches as the foreground.

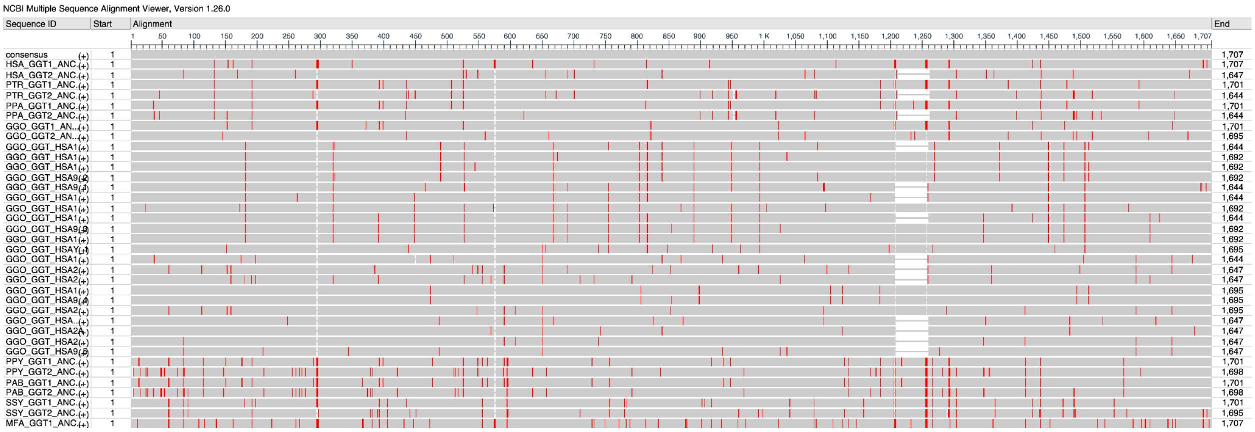

Figure S35. CDS multiple sequence alignment visualization for *GGT*. Full MSA of the *GGT* CDS with consensus mismatches highlighted in red and gaps in white. All gaps were trimmed before selection analyses.

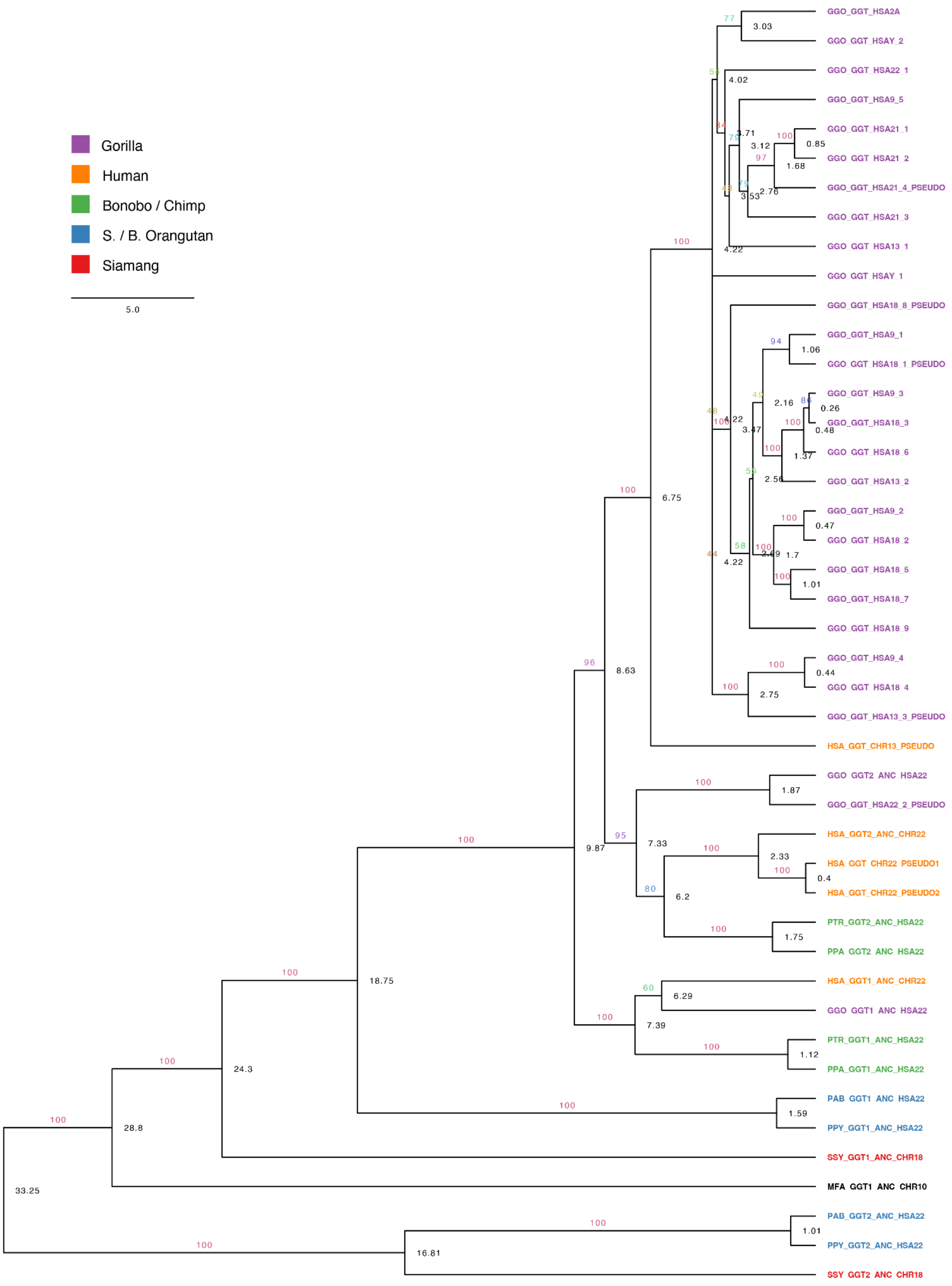

Figure S36. Time-measured intron phylogeny for *GGT*. Tree was rooted with marmoset *GGT1* (branch not shown) and estimated divergence times of 28.8 Ma and 43 Ma were used for the human/macaque and human/marmoset splits, respectively. Tips are colored by species, branches by bootstrap, and nodes by estimated time in Ma.

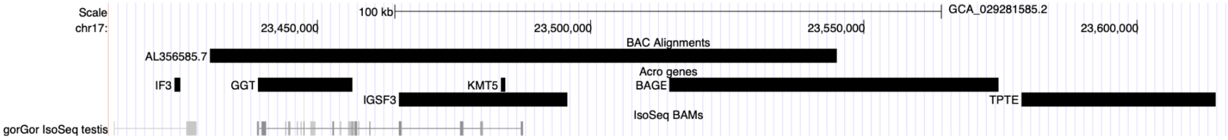

Figure S37. Alignment of BAC RP11-341D18 to the gorilla genome. Human BAC RP11-341D18 (AL356585.7) is shown aligned to gorilla Chr17 (HSA18) overlapping the amplified gorilla genes: *IGSF3-GGT* (fusion) and *BAGE2*. Overlaid transcript evidence from gorilla testes is shown at bottom.

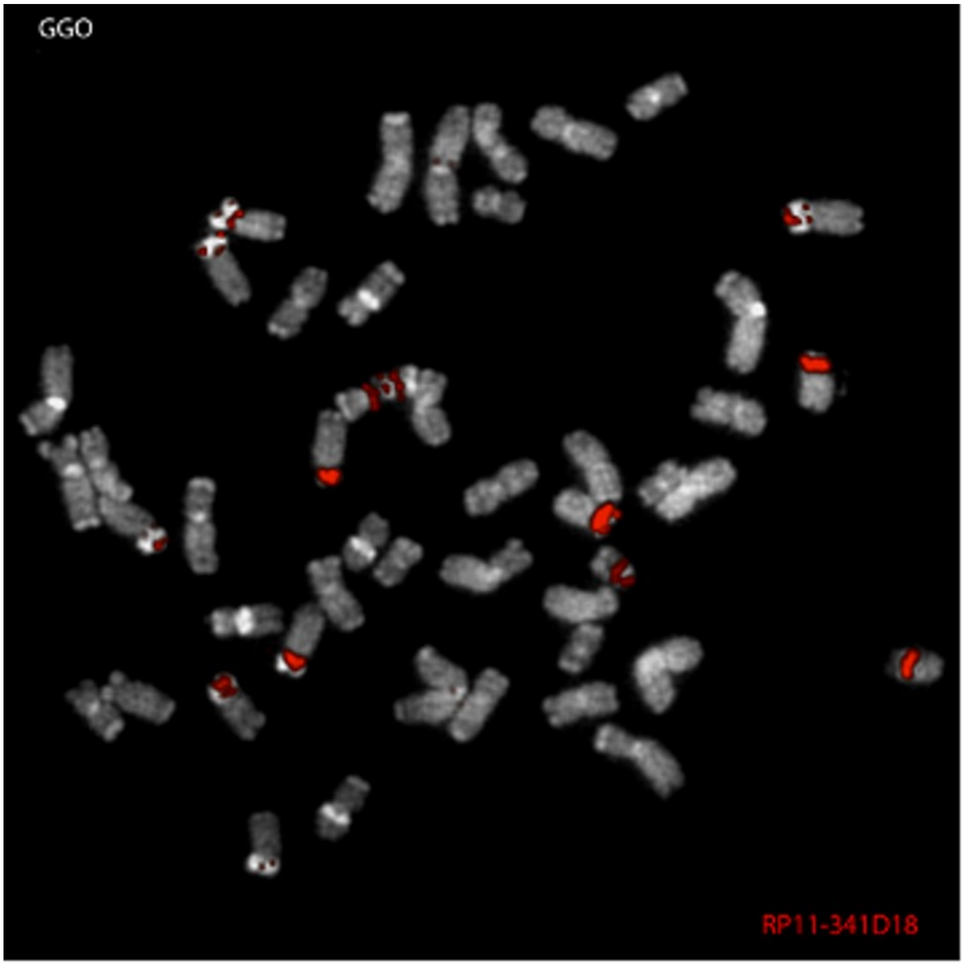

Figure S38. Metaphase FISH of the *GGT* amplification in gorilla. FISH analysis using BAC probe RP11-341D18 (AL356585.7) mapping to the amplified *IGSF3-GGT* locus on metaphase chromosomes from *Gorilla gorilla gorilla* (individual "GGO"), confirming amplification across the NOR– acrocentrics. Consistent hybridization patterns were observed across six different gorilla individuals as summarized in Supplementary Table S8.

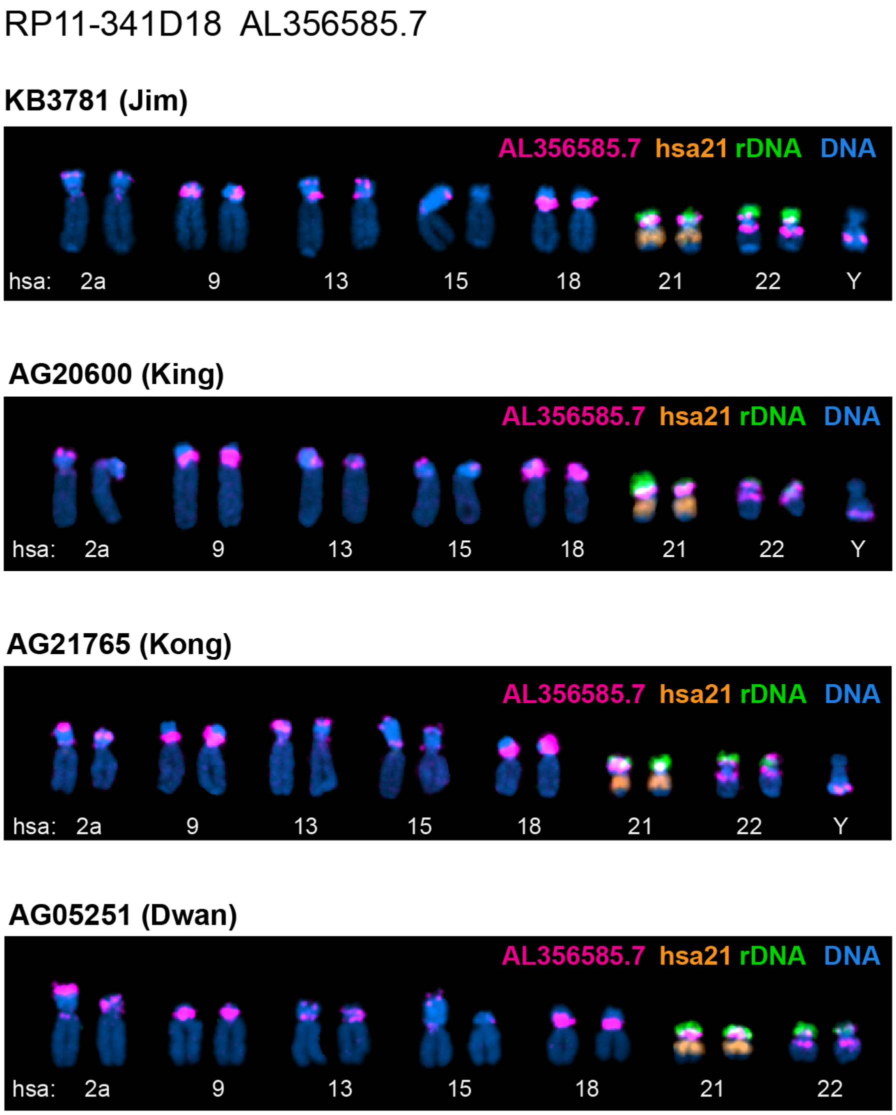

Figure S39. Chromosome-labeled FISH of the *GGT* amplification in gorilla. Representative acrocentric karyograms from primary fibroblast cell lines of four different gorilla individuals (3 male, 1 female) labeled by FISH using BAC probe RP11-341D18 (AL356585.7, magenta). rDNA was labeled with BAC RP11-450E20 (green) and HSA21 was labeled with human Chromosome 21 paint (orange). Chromosomes were counterstained with DAPI. The *IGSF3-GGT* locus is consistently amplified across HSA9/18/21, with scattered distal copies on the other short arms, consistent with the T2T genome annotation. The ancestral location of *GGT1/2* on the long arm of HSA22 is also shown. Note that “Jim” is the same animal used for the T2T reference genome assembly.

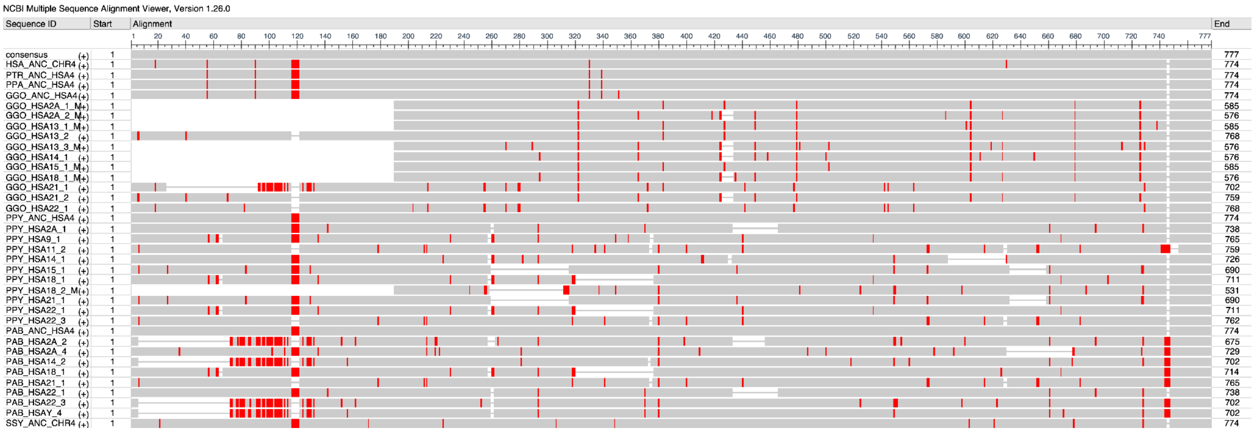

Figure S40. CDS multiple sequence alignment visualization for *FRG1*. Full MSA of the *FRG1* CDS with consensus mismatches highlighted in red and gaps in white. Modified *FRG1* gene models from gorilla begin around position 190 bp. All gaps (including those caused by the modified starts) were trimmed before selection analyses.

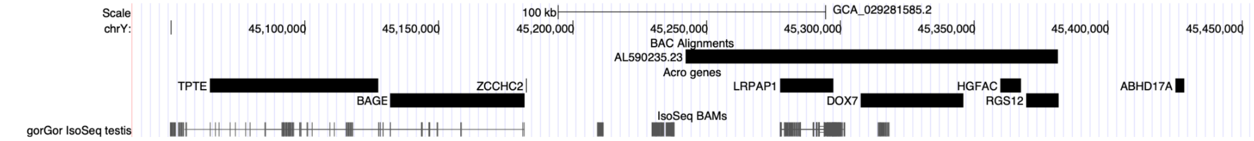

Figure S41. Alignment of BAC RP11-529E10 to the gorilla genome. Human BAC RP11-529E10 (AL590235.23) is shown aligned to gorilla ChrY overlapping the amplified gorilla gene *LRPAP1*. Overlaid transcript evidence from gorilla testes is shown at bottom.

RP11-529E10 AL590235.23

KB3781 (Jim)

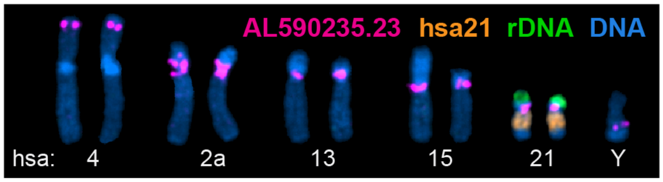

AG20600 (King)

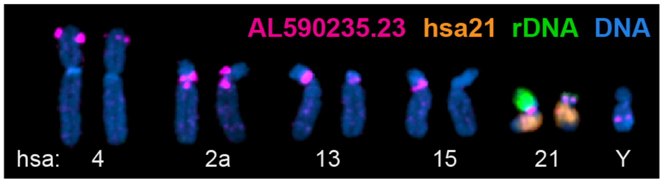

AG21765 (Kong)

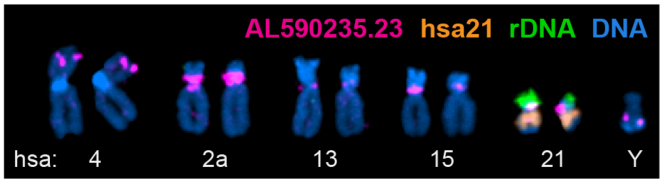

AG05251 (Dwan)

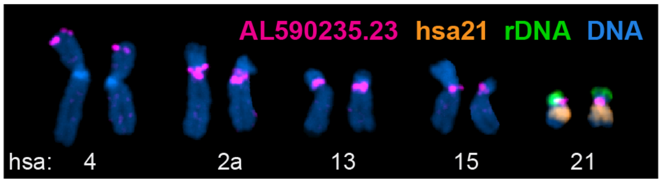

Figure S42. Chromosome-labeled FISH of the *LRPAP1* (*FRG1*) amplification in gorilla. Representative acrocentric karyograms from primary fibroblast cell lines of four different gorilla individuals (3 male, 1 female) labeled by FISH using BAC probe RP11-529E10 (AL590235.23, magenta) mapping to the *LRPAP1* locus. rDNA was labeled with BAC RP11-450E20 (green), HSA21 was labeled with human Chromosome 21 paint (orange). Chromosomes were counterstained with DAPI. Based on the gorilla genome annotation, *LRPAP1* genes are typically found in the same amplified cluster as *FRG1*. Amplification of this locus across the pericentromeres of HSA2A/13/15 and HSA21 is visible, consistent with the T2T genome annotation. The ancestral location of *LRPAP1* on the short arm of HSA4 is also shown. Note that "Jim" is the same animal used for the T2T reference genome assembly.

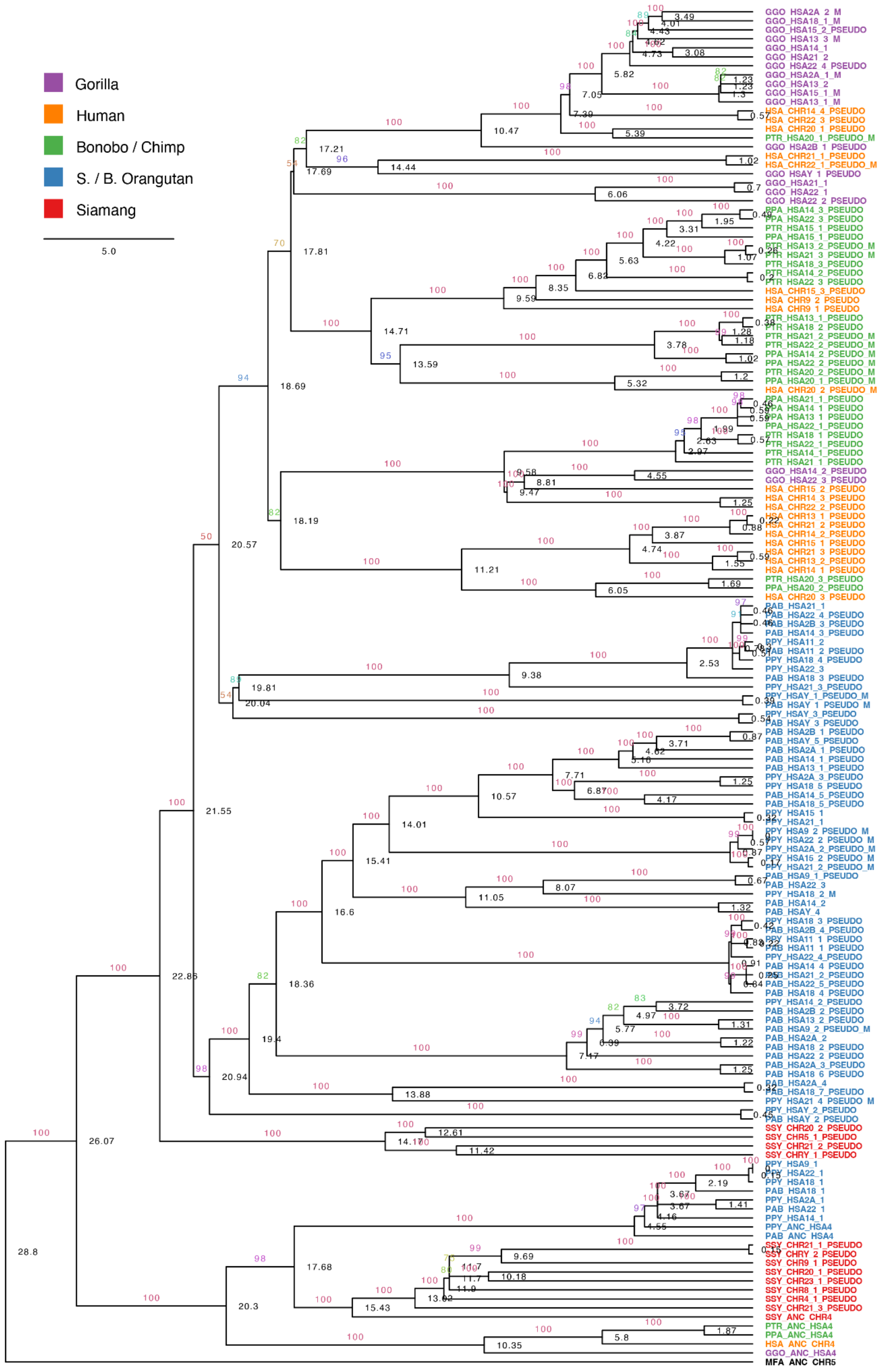

Figure S43. Time-measured intron phylogeny for *FRG1*. Tree was rooted with marmoset *FRG1* (branch not shown) and estimated divergence times of 28.8 Ma and 43 Ma were used for the human/macaque and human/marmoset splits, respectively. Tips are colored by species, branches by bootstrap, and nodes by estimated time in Ma.

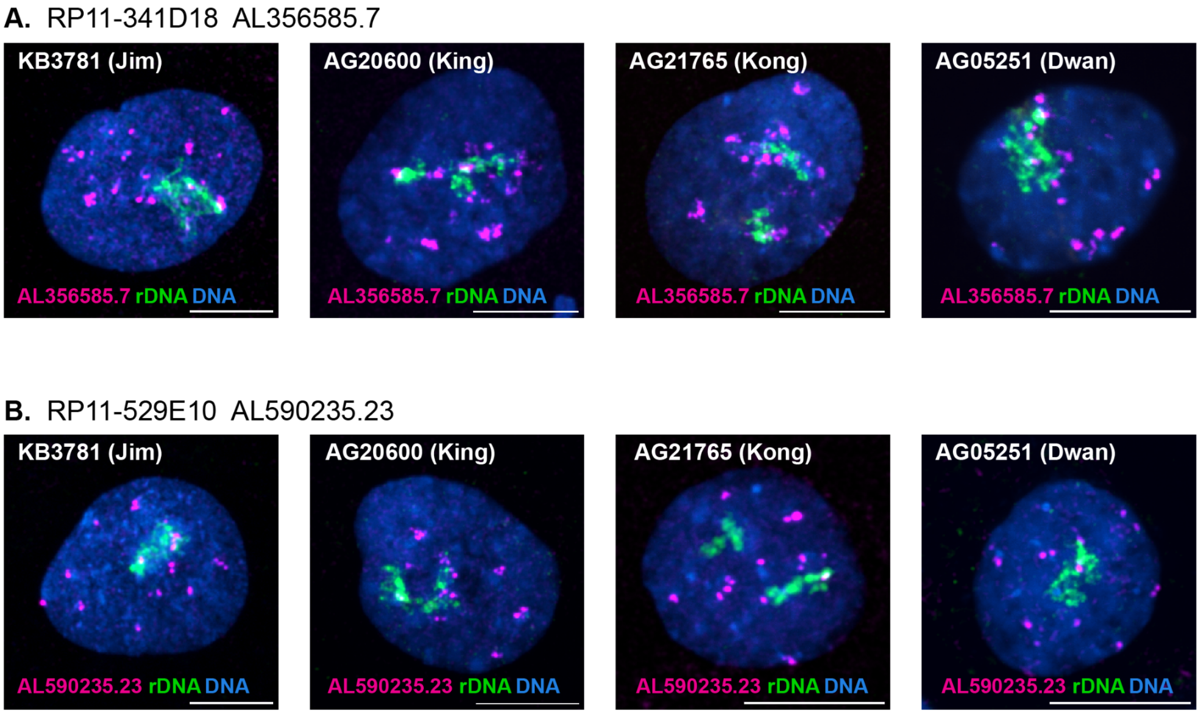

Figure S44. Localization of amplified gene regions and rDNA in representative interphase fibroblast nuclei of four different gorillas. (A) Most *GGT*-associated signals from BAC RP11-341D18 (AL356585.7, magenta) were not associated with rDNA (BAC RP11-450E20, green). (B) Nor were the *LRPAP1*-associated signals from BAC RP11-529E10 (AL590235.23). This suggests the gorilla NOR– chromosomes do not organize with the NOR+ chromosomes and may not participate in the formation of nucleoli. Nuclei were counter-stained with DAPI. Bar, 10μm.
